# Insights on *Pseudomonas aeruginosa* Carbohydrate Binding from Profiles of Cystic Fibrosis Isolates Using Multivalent Fluorescent Glycopolymers Bearing Pendant Monosaccharides

**DOI:** 10.3390/microorganisms12040801

**Published:** 2024-04-16

**Authors:** Deborah L. Chance, Wei Wang, James K. Waters, Thomas P. Mawhinney

**Affiliations:** 1 Department of Molecular Microbiology & Immunology, University of Missouri School of Medicine, Columbia, MO 65212, USA; 2 Department of Pediatrics, University of Missouri School of Medicine, Columbia, MO 65212, USA; mawhinneyt@missouri.edu; 3 Department of Biochemistry, University of Missouri, Columbia, MO 65211, USA; w_w@fudan.edu.cn; 4 Experiment Station Chemical Laboratories, University of Missouri, Columbia, MO 65211, USA; watersj@missouri.edu

**Keywords:** *Pseudomonas aeruginosa*, cystic fibrosis, carbohydrate, monosaccharides, binding profiles, binding assays, fluorescent glycopolymers, phenotypic heterogeneity, microscopy, spectrofluorometry

## Abstract

*Pseudomonas aeruginosa* contributes to frequent, persistent, and, often, polymicrobial respiratory tract infections for individuals with cystic fibrosis (CF). Chronic CF infections lead to bronchiectasis and a shortened lifespan. *P. aeruginosa* expresses numerous adhesins, including lectins known to bind the epithelial cell and mucin glycoconjugates. Blocking carbohydrate-mediated host–pathogen and intra-biofilm interactions critical to the initiation and perpetuation of colonization offer promise as anti-infective treatment strategies. To inform anti-adhesion therapies, we profiled the monosaccharide binding of *P. aeruginosa* from CF and non-CF sources, and assessed whether specific bacterial phenotypic characteristics affected carbohydrate-binding patterns. Focusing at the cellular level, microscopic and spectrofluorometric tools permitted the solution-phase analysis of *P. aeruginosa* binding to a panel of fluorescent glycopolymers possessing distinct pendant monosaccharides. All *P. aeruginosa* demonstrated significant binding to glycopolymers specific for α-D-galactose, β-D-*N*-acetylgalactosamine, and β-D-galactose-3-sulfate. In each culture, a small subpopulation accounted for the binding. The carbohydrate anomeric configuration and sulfate ester presence markedly influenced binding. While this opportunistic pathogen from CF hosts presented with various colony morphologies and physiological activities, no phenotypic, physiological, or structural feature predicted enhanced or diminished monosaccharide binding. Important to anti-adhesive therapeutic strategies, these findings suggest that, regardless of phenotype or clinical source, *P. aeruginosa* maintain a small subpopulation that may readily associate with specific configurations of specific monosaccharides. This report provides insights into whole-cell *P. aeruginosa* carbohydrate-binding profiles and into the context within which successful anti-adhesive and/or anti-virulence anti-infective agents for CF must contend.

## 1. Introduction

Recurrent and chronic respiratory tract infections and airway obstruction are constant challenges for individuals with the genetic disorder cystic fibrosis (CF) [1,2,3,4,5,6,7,8]. In CF, ineffective mucociliary clearance, repeated and cyclic exacerbations in infection and inflammation, shifts in microbial populations, and increasing antibiotic pressures all contribute to airway obstruction and lung damage, eventually creating life-threatening scenarios [1,6,7,8,9,10].

While CF infections are often polymicrobial [8,11], the opportunistic pathogen *Pseudomonas aeruginosa* is frequently present and a major player in the complex CF airway dynamics [1,6,12]. *P. aeruginosa* readily colonizes the compromised CF airway, adapts to the host environment, and develops a decreased sensitivity to antibiotics [10,13,14,15,16,17,18,19,20]. *Pseudomonas* growth and persistence in the biofilm mode in the CF respiratory tract, as seen in wounds as well, contribute to the therapeutic challenge, affecting both antibiotic efficacy and host inflammatory immune responses [6,7,17,20,21,22,23,24,25,26,27]. Phenotypic fluctuations of *P. aeruginosa* include changes in motility, exopolysaccharide production (such as type and amount of alginate, Pel, and/or Psl), and antibiotic susceptibility [6,10,11,13,14,16,19,28,29,30,31]. Within individuals with CF, the regional microevolution of subpopulations further increases the diversity of *P. aeruginosa* for which treatment may be sought [10,15,16,17,19,32,33,34]. Once chronic colonization is established, *P. aeruginosa* is rarely completely eradicated [6,8,10].

As multi-drug resistance and conditions of antibiotic tolerance continue to increase globally among *P. aeruginosa* and other major pathogens responsible for drug-resistant nosocomial infections, new approaches and tools to complement the existing repertoire of anti-microbial agents are urgently needed [8,12,27,35,36,37,38,39,40]. Successful adjunctive therapies for CF airway infection, ideally, will accommodate the heterogeneity of the biological features and activities of *P. aeruginosa* in the CF population and within the individual host over time, and the variability of the host pulmonary status [1,6,16,17,18,19,20,28,30,33,34,41,42,43,44,45]. Tactics and adjunctive agents developed to counter CF *Pseudomonas* respiratory infections are anticipated to also benefit other problematic host infections [12,23,26,35,37,40,46,47], and aid in attempts at antibiotic conservation. The current study aims to contribute basic science observations to the ongoing advancement of new strategies for therapeutic intervention in CF airway infection related to the carbohydrate-binding patterns of clinical isolates of *P. aeruginosa*.

The management of airway obstruction and infection in CF is currently multi-faceted and is discussed elsewhere in numerous publications; a few examples are given here [3,4,5,6]. Briefly, treatments commonly include regular chest physiotherapy, bronchodilators, mucus disruption and clearance with recombinant DNase and mucolytics, and antimicrobial regimens with a variety of oral, i.v., and inhaled antibiotics [5,6,39,48,49,50]. Inhaled antibiotics, such as tobramycin and aztreonam, are tolerated by many patients, and provide higher drug concentrations at the site of infection, and less host toxicity, than administration via other routes [3,4,6,39,48]. Other individuals, however, cannot tolerate the currently available agents, or are colonized by multiple-drug resistant (MDR) *P. aeruginosa*, thus emphasizing the need for new agents, and/or combinatorial approaches to restore antibiotic potencies without increasing toxicity [3,48]. The therapeutic arsenal of the future is anticipated to include new anti-inflammatory agents [4,5,39], complimentary agents to improve mucus viscoelasticity and clearance [2,51], additional aerosolized antibiotics [3,4,6,39], new pairings of antibiotics, and combinations of antibiotics with other agents to enhance antimicrobial effectiveness (adjuvants) [3,5,6,27,36,37,39,40,48,50,52,53,54]. 

Attractive goals for novel adjunctive agents include reducing CF *Pseudomonas* virulence, aggregation, adhesion, biofilm development, and biofilm matrix integrity [6,7,12,21,24,27,35,38,42,44,52,55,56,57]. Anti-infectives intended to limit virulence, for example, may interfere with the quorum-sensing systems of bacteria, and alter the virulence factor expression during host adaptation and the establishment of protective biofilms [7,8,12,26,38,56,58,59]. Anti-adhesives designed to interfere with the functions of bacterial carbohydrate-binding proteins (lectins) may limit and/or disrupt bacterial lectin–host interactions, bacterial aggregation, and lectin–biofilm matrix associations [12,22,27,35,52,55,57,59,60,61]. The specific targeting of antimicrobial agents to bacterial biofilms, as suggested by recent investigations, may also be possible [22,54,59,62,63,64], and not only aid in achieving appropriate therapeutic dosages where needed in the CF airway, but also benefit and guide efforts toward antibiotic stewardship.

A significant body of knowledge exists about the basics of in vitro *Pseudomonas* adhesion to inform current anti-adhesion therapeutics development, some details of which are included below [9,36,47,65,66,67,68,69,70,71,72,73,74,75,76,77,78,79,80,81,82,83,84]. Key observations and inferences highlighted here come from foundational experiments, often gained using strains of *P. aeruginosa* such as PAO1, PAK, PA14, and their derivatives [68,69,70,71,72]. Early laboratory studies indicated that *Pseudomonas* adhesins, such as pili, flagella, and lectins, assisted in the colonization and maintenance of infection of the airway epithelia and its mucus component [60,69,73,74,75,76]. In the laboratory, *P. aeruginosa* recognized a number of carbohydrate determinants [65,66,73,74,77,78], including asialoganglioside GM1 (aGM1) on CF cells in culture [78]. *Pseudomonas* pili contributed to the binding of the apical airway cell surface glycosphingolipids, asialoganglio-*N*-tetraosylceramides asialo-GM1, and asialo-GM2 [36,65,68,71,79], possibly through the terminal sugars β-D-galactose (βGal) and β-D-*N*-acetylgalactosamine (βGalNAc) of asialo-GM1 and asialo-GM2, respectively. Flagella bound heparan sulfate proteoglycans on both apical and basolateral epithelial cell surfaces [36,68]. The presence of such complex glycoconjugates in damaged and immature epithelia, i.e., where there is prior lung damage, suggests that such interactions via pili and flagella would favor *Pseudomonas* colonization [36,68,73]. The flagellar cap protein FliD and flagellin were found to be important for bacterial mucin adherence [69,75,80] and, in some cases, were found to bind specific Lewis (Le) antigen glycotopes (carbohydrate epitopes) [9,66,67,75]. Using fluorescent glycoconjugates representing a variety of peripheral oligosaccharides of mucous glycoproteins, early investigations with several laboratory and CF clinical isolates of *P. aeruginosa* found strain-specific binding to one or more of the neoglycoconjugates for Le^a^, Le^x^, Le^y^, sialyl-Le^x^, 3′-sulfo-Le^x^, 6-sulfo-sialyl-Le^x^, or Gal(α1-2)Gal [65,81]. These findings were particularly important in light of evidence of the increased prevalence of mucin oligosaccharide sialylation and sulfation in situations of increased inflammation, as in chronic bronchitis and cystic fibrosis [65,67,82,83,84]. Such increased expression of sialyl- and sulfo-Lewis antigens in the respiratory tree was suggested to contribute to the propensity of *P. aeruginosa* to colonize the CF host [36,49,65,67,81,85]. 

*Pseudomonas* lectins are currently thought to contribute to CF airway colonization in several ways and are considered potential targets for interventional agents [9,22,30,61,62,86,87,88,89]. Lectin studies focused first on their roles in bacterial agglutination, attachment, and virulence, and, then, on the regulation and inhibition of binding [47,58,70,76,88,90,91,92]. LecA and LecB (lectins previously referred to as PA-IL and PA-IIL) were, respectively, found to preferentially bind α-D-galactose (and, with a lesser affinity, β-N-acetyl-galactosamine) and α-L-fucose (and, to a lesser degree, mannose) [47,60,93,94,95,96]. Purified LecB demonstrated an affinity for the prominent α-L-fucose of the Lewis and blood group antigens, suggesting its involvement in the adherence in vivo to mucins and epithelial cell glycosphingolipids [60,91,97]. Virulence appeared to be associated with the extracellular presence of LecA and LecB, either as soluble proteins or as membrane-associated structures at the outer membrane or on outer membrane vesicles (OMVs) [47,70,88,92,98]. Extracellular LecA and LecB were reported to diminish the activity of cilia [76,86,87,99] and to contribute to lung injury [70]. Early on, a therapeutic advantage was observed with the inhalation of the simple sugars galactose and fucose, possibly by interfering with the lectin–cilia binding [100,101]. Data suggested that inhaled sugars might compete with the detrimental host–lectin associations to assist in the resolution of the bacterial infection [46,87,100,101]. Additional laboratory findings indicated that the mixture of mannose, galactose, and fucose could synergize with antibiotics, potentially serving as an adjunctive therapy to reduce colonization and airway damage [60]. Large clinical trials, however, have not followed.

These lectins are now known to also be involved in the formation and maintenance of the protective *Pseudomonas* biofilm [22,47,59,61,62,88,89,90,92,98,102], making them especially significant to CF *P. aeruginosa* airway infections and their treatment. For example, LecA and LecB were found to associate with two bacterial-derived exopolysaccharides (EPS) Pel and Psl to help build and maintain the biofilm integrity [22,89]. Renewed investment in anti-lectin therapeutics came, following several key avenues of investigations. Studies targeting LecB activities demonstrated that C-fucosyl peptide dendrimers could inhibit LecB [103], and that multivalent fucosyl-peptide dendrimers could both inhibit *Pseudomonas* biofilm formation and disrupt existing biofilms [104,105,106]. Multivalent galactosylated glycopeptide dendrimers aimed at LecA also interfered with biofilm establishment and stability [106,107]. Laboratory models showed that the administration of *N*-acetylgalactosamine, and methyl glycosides of galactose and fucose [70], or multivalent glycoclusters functionalized with galactosides or fucosides [98], respectively, reduced lung injury due to *Pseudomonas* infection [70,98]. Investigations with nasal mucosa explants possessing actively beating ciliary cells demonstrated that select monosaccharides and glycomimetic peptides were also able to modulate the *Pseudomonas* lectin-induced reduction in the ciliary beat frequency (CBF) [76,87,99]. 

To target *P. aeruginosa* cellular and extracellular carbohydrate interactions, many types of glycomimetics and multivalent glycoconjugates have been suggested as potential anti-infectives and are in various stages of investigation [44,52,54,55,57,59,61,105,106,108,109,110,111]. Emphasizing the inhibition of the binding of the *Pseudomonas* lectins, examples include small molecule glycomimetics, glycoclusters, heteroglycoclusters, heteromultivalent gold nanorods, glycodendrimers, glycopolymers, aromatic thioglycosides, glyconanoparticles, and nanotherapeutics with glycomimetic shells [25,52,55,57,61,63,97,103,104,105,106,107,109,110,111,112,113]. Bioavailable, monovalent, low-molecular-weight glycomimetic inhibitors of LecB were shown to inhibit biofilm formation, illustrating the potential for this strategy as a new class of antimicrobial drugs [114]. As recently reviewed, a wide variety of glycomimetic lectin inhibitors with favorable in vivo characteristics are actively being evaluated [111].

As with the development of any novel therapeutic strategy [56], there are a number of concerns regarding the design of agents to inhibit lectins or other adhesins. The two most significant in CF surround the possibility of drug-induced bacterial aggregation, and the heterogeneity with which *P. aeruginosa* may express the potential drug targets. With multivalent anti-adhesive agents as adjuvants to conventional antibiotic regimens, there would be an added risk of bacterial aggregation facilitated via the multivalent drug interactions [98,110,112]. As aggregates are known to be more antibiotic- and DNase-tolerant, adjuvant-induced aggregation may contribute to a reduced sensitivity to routine anti-*Pseudomonal* therapeutics [14,24]. The elaboration of lectins and other adhesive extracellular features are known to vary between *P. aeruginosa* strains and with environmental growth conditions [10,29,36,56,58,64,70,73,88,90,93,114,115]. LecA and LecB proteins, in vitro, for example, appeared to be the most abundant during the stationary phase [90,93]. Compared with the laboratory reference strains PAO1 and PAK, *P. aeruginosa* were shown to make different types of flagellar Cap protein FliD (types A and B), and these types affected interactions with mucin carbohydrate epitopes [75]. Niche-specific adhesin expression was also suggested by studies of *P. aeruginosa* from ocular and urinary tract infections [116]. Differences among the isolates were found in tear glycoprotein binding and in its inhibition by simple sugars and N-glycans from tears and other sources [116]. Genomic alterations in pilin genes and *lecB* were also discovered through a survey of *P. aeruginosa* strains, [73,117,118]. If such distinctions between *Pseudomonas* in vivo exist and affect binding affinities, they may also be expected to impact the range of effectiveness of lectin-targeted inhibitory agents across patient populations and/or over time for a given individual. 

The aim of the current study, toward informing the ongoing development of adjunctive therapeutics for the treatment of CF airway infection, is to address two questions related to the carbohydrate binding of clinical isolates of *P. aeruginosa* from CF and non-CF sources. The first, in whole-cell solution-phase binding assays, is as follows: which simple sugars appear to interact with the living cells of *P. aeruginosa*? The second is as follows: does this binding vary with the source or other characteristic(s) of the bacterial specimens? With the variable nature of *P. aeruginosa* in CF within an individual, between acute and chronic infections, and across CF populations [10,19,28,30,32], this secondary question is of particular importance for future therapeutic interventions intended to target the widest variety of CF *P. aeruginosa* infections. Here, we report on our survey of the carbohydrate-binding profiles of a panel of eighteen *P. aeruginosa* with nine fluorescent glycopolymers, with pendant monosaccharides representing common terminal structures on airway mucins and epithelia [49].

## 2. Material and Methods

### 2.1. Materials

Polyacrylamide (PAA)-based multivalent fluoresceinated glycopolymers used in this study possessing specific pendant monosaccharide side chains are listed in Table 1. The glycopolymers possessing the general structure scheme depicted in Table 1 and the negative control polymer HOCH_2_(HOCH)_4_CH_2_NH-PAA-Fluor (referred to as aminoglucitol-PAA-Fluor) were purchased from GlycoTech (Gaithersburg, MD, USA). The α-L-Fucose-PAA-Fluor was both purchased from GlycoTech and synthesized in house, as briefly described below and in more detail with analysis in the Supplemental File S1. Prepared microbiological agar plates were purchased from Remel (Lenexa, KS, USA). M9 minimal media salts, trypticase soy broth, bovine serum albumin (BSA), sodium dodecyl sulfate (SDS), L-fucose, melibiose, 4-nitrophenyl α-D-fucopyranoside, D-mannose-coated agarose beads, and *Pseudomonas aeruginosa* lectin LecA (PA-IL) were obtained from Sigma-Aldrich (St. Louis, MO, USA). *P. aeruginosa* lectin LecB (PA-IIL) was purchased from Elicityl SA (Crolles, France). D-Galactose-coated agarose beads were from Pierce Biotechnology (Rockford, IL, USA). Anti-FITC gold-conjugated antibody was purchased from Electron Microscopy Sciences (Hatfield, PA, USA). Spin-X centrifuge tube filter units with 0.22 μm-pore-size nylon membranes made by Corning (Corning, NY, USA), GelCode blue stain reagent, fluorescent stains, fixatives, and routine and mass spectrometry grade solvents were purchased through Fisher Scientific (Division of Thermo Fisher Scientific, St. Louis, MO, USA). Precast polyacrylamide gels and gel running supplies were from BioRad (Bio-Rad Laboratories, Hercules, CA, USA). Agarose beads coated with *Griffonia simplicifolia* lectin I, *Erythrina cristagalli* lectin, soybean agglutinin, wheat germ agglutinin, and *Aleuria aurantia* lectin were purchased from Vector Laboratories (Burlingame, CA, USA), and *Limulus polyphemus* lectin beads were from EY Laboratories (San Mateo, CA, USA).

### 2.2. Synthesis α-L-Fucose Fluoresceinated Glycopolymer

α-L-Fuc-PAA-Fluor was prepared using a controlled copolymerization strategy [119]. The synthetic steps are detailed in the Supplemental File S1. In brief, α-L-fucopyranosyl-(1→6)-D-glucose was prepared according to a published method with some modifications [120,121]. The disaccharide was then oxidized to lactone, and reacted with *N*-(2-aminoethyl) methacrylamide hydrochloride to form the glycomonomer 6-*O*-α-L-fucopyranosyl-D-gluconamidoethyl methacrylamide. Then, using a reversible addition–fragmentation chain-transfer (RAFT)-based copolymerization, the tri-component linear statistical glycopolymer was prepared. The glycopolymer was characterized by gel permeation chromatography (GPC), and then nuclear magnetic resonance (NMR) confirmed its structure (NMR data reported in Supplemental File S1, Figure S1). The glycopolymer was fluoresceinated through reaction with carboxyfluorescein succinimidyl ester. The binding specificity, as described below, was confirmed with *Aleuria aurantia* lectin-coated agarose beads. 

### 2.3. Glycopolymer Specificity Verification

The carbohydrate-binding specificities of the polyacrylamide-based fluorescent glycopolymers used in this study were verified prior to use via a previously published method employing lectin-coated agarose beads and fluorescence microscopy [119]. Lectin beads used and the specificities are as follows: *Griffonia simplicifolia* lectin I for α-Gal; *Erythrina cristagalli* lectin for β-Gal; soybean agglutinin for GalNAc; *Limulus polyphemus* lectin for α-Neu5Ac; wheat germ agglutinin for β-GlcNAc; and *Aleuria aurantia* lectin for α-L-Fuc. The protocol followed this example: a slurry of soybean agglutinin-coated agarose beads (100 µL) was pre-washed with phosphate-buffered saline (PBS) (3 × 1 mL) and resuspended in 0.5 mL PBS-Ca (PBS with 1 mM CaCl_2_), prior to the addition of 20 µL of sodium phosphate buffer (0.3 M, pH = 7.4) containing 10 µg of α-GalNAc-PAA-Fluor. Incubation proceeded at 35 °C for 1 h with gentle mixing, and was followed by gentle centrifugation (2000× *g* at 4 °C, for 3 min) and two rinses with PBS (1 mL PBS). The rinsed beads were resuspended in 200 µL of PBS and observed by fluorescence microscopy for the presence of bound fluorescent PAA-glycopolymer.

### 2.4. Bacteria

#### 2.4.1. Clinical Isolates and Laboratory Strains Sources, Characteristics, and Cultures

As detailed in the Institutional Review Board Statement at the end of this article, de-identified human specimens and data generated in this IRB-exempt human research investigation were gathered and used in accordance with institutional and national guidelines.

The *Pseudomonas aeruginosa* evaluated in this study are listed in Table 2. Clinical isolates were obtained from cystic fibrosis (CF) and non-CF patient specimens analyzed at the Diagnostic Laboratories of the University of Missouri Health Care Hospital and Clinics, Columbia, MO. Laboratory strains were acquired through the American Type Culture Collection (ATCC, Manassas, VA, USA). Verifications of *P. aeruginosa* identities were performed and metabolic activity profiles were generated with the automated identification system VITEK^®^ 2 (BioMérieux, Durham, NC, USA). Isolate/strain phenotypic traits, including colony morphology, were determined following culture on solid agar media at 35 °C with 5% CO_2_, including tryptic soy, MacConkey, *Pseudomonas* P, *Pseudomonas* F, and blood agars. Functional pili twitching assay was performed with Luria–Bertani agar (LB) using 1.5% agar [122]. Stocks of clinical isolates and laboratory strains were stored at −80 °C in trypticase soy broth (TSB) with 40% glycerol.

#### 2.4.2. *P. aeruginosa* Structural Features Assessment by Transmission Electron Microscopy

Characteristic structural features of each *P. aeruginosa* strain, such as presence or absence of detectable flagella and/or pili, were assessed by transmission electron microscopy (TEM). Aqueous suspensions of bacteria were applied to carbon-coated copper grids, briefly tungsten-stained on-grid (nano-W, Nanoprobes, Yaphank, NY, USA), and air-dried, as previously described [128]. Additional method detail is provided in Supplemental File S2. Images were acquired on a JEOL JEM-1400 Transmission Electron Microscope with DigitalMicroscope software v. 3.0.1 (Gatan, Pleasanton, CA, USA). TEM micrographs were adjusted for appropriate brightness and contrast using levels in Adobe Photoshop 2020 software (Adobe, San Jose, CA, USA).

#### 2.4.3. Preparation of Bacteria for Binding Experiments

*P. aeruginosa* for glycopolymer-binding experiments were first grown on tryptic soy agar at 35 °C with 5% CO_2_ and the colony morphology typical of the strain verified. Bacteria were then liquid-cultured at 35 °C, 150 rpm, either in M9 minimum media with glucose [127] with incubation routinely for 35 h, or in TSB for 16 h (depending on whether isolate was culturable in minimum media; exceptions are noted in Table 2). Cultures were centrifuged at 9000× *g* at 4 °C for 15 min, and the pellets resuspended in filter-sterilized binding solution (155 mM NaCl, 1 mM CaCl_2_, 0.5% BSA, and 0.05% SDS in distilled H_2_O) and diluted to attain bacterial suspensions with an optical density (OD) of 1 at 600 nm, averaging ~4 × 10^9^ CFU input bacteria per mL. CFUs/mL characteristic of each strain were determined by serial dilutions and plate counts.

### 2.5. P. aeruginosa Carbohydrate-Binding Assays Using Fluorescent Glycopolymers

#### 2.5.1. Solution-Phase Binding Reactions

*P. aeruginosa* carbohydrate binding was assessed via solution-phase binding assays with the pendant monosaccharide-possessing multivalent fluorescent PAA-based glycopolymers detailed in Table 1. Typically for each bacterial binding test, 18 µg of glycopolymer dissolved in 42 µL of sodium phosphate buffer (0.3 M, pH = 7.4) was added to 1.0 mL of bacterial suspension. Negative controls for binding reactions included bacterial suspensions to which no glycopolymer was added, and to which fluoresceinated PAA-polymers possessing only terminal aminoglucitol was added. Binding reaction mixtures were then incubated at 35 °C in the dark for 2 h with gentle shaking, followed by two repeated sets of centrifugation (9000× *g*, 4 °C, 15 min) and saline rinses (1.2 mL of 155 mM NaCl containing 1 mM CaCl_2_). The resulting bacterial pellets were resuspended in 100 µL of PBS (pH = 8). Bacterial binding experiments were performed in triplicate. Assay variables evaluated included length of culture prior to harvest for binding assays (i.e., 16 h vs. 35 h in M9), increased amount of glycopolymer in binding reactions, and decreased washing steps following binding.

#### 2.5.2. Detection of Bacterial-Glycopolymer Binding

Bacterial-glycopolymer binding was routinely detected via spectrofluorometry and fluorescence microscopy. Fluorescence intensity (arbitrary units, AU; λex/λem = 490/520 nm, slit width = 10 nm) and optical density (OD_600_ nm) of rinsed glycopolymer-bound bacterial suspensions were measured in a multi-mode microplate spectrophotometer (Synergy MX, BioTek, Winooski, VT, USA). Fluorescence signals were normalized to sample optical densities. To permit comparisons across glycopolymers and strains, after subtracting the values from the parallel samples without glycoconjugates, the sample data were then normalized to adjust for the fluorescence indices of the respective PAA-Fluor glycopolymers. Fluorescence indices of polymers with pendant monosaccharides, normalized to the aminoglucitol control polymer, ranged from 0.61 to 1.63, and are as follows: α-Gal, 0.61; β-Gal, 0.88; α-GalNAc, 1.24; β-GalNAc, 1.63; β-GlcNAc, 1.1; β-Gal3S, 0.69; β-GalNAc3S, 0.86; α-Neu5Ac, 0.98; and α-Fuc, 1. Data were reported as fluorescence intensities for triplicate bacterial binding experiments and plotted with standard deviations (STDEV) using Microsoft Excel 2016 software.

For survey and quantitation of bacterial-glycopolymer binding with fluorescence microscopy, glycopolymer-bound bacterial suspensions were first applied to multi-welled immunofluorescence microscope slides (Polysciences), observed, and photographed on a fluorescence microscope (Olympus BX43, 10X objective, FITC filters, with cellSens Dimension software v. 1.7.1, Olympus, Waltham, MA, USA). Fluorescent glycopolymer-bound *P. aeruginosa* were then enumerated with the software-assisted fluorescent cell count feature. As above, cell count data reflect averages of multiple experiments and include CFU index normalization. Fluorescent glycopolymer-bound bacteria were also differentiated from non-fluorescing bacteria at higher magnification (100X objective) via overlays of fluorescence images with either brightfield images of unstained specimens, or images obtained for 4′,6-diamidine-2′-phenylindole (DAPI)- and propidium iodide (PI)-stained specimens with the appropriate fluorescence filters. This approach and preliminary data were reported in a published conference abstract [129].

### 2.6. Localization and Population Distribution of Bacterial-Bound Glycopolymer with TEM and Flow Cytometry

TEM and flow cytometry were employed to further evaluate the localization of glycopolymer binding and its distribution on individual bacterial cells within representative experiments. 

TEM imaging of glycopolymer on bacteria was performed after immunolabeling of the bacterial-bound glycopolymer with gold-conjugated anti-fluorescein antibody, as follows: Binding experiment bacterial suspensions were applied to grids (by floating carbon-coated nickel grids on the suspensions for 10 min, then wicking off excess solution and air-drying), then immunofixed (4% paraformaldehyde in PBS, 30 min, room temperature), rinsed (PBS, 2 × 5 min), and blocked with PBS with 0.05% Tween-20 (*v*/*v*) and 1% BSA (PBS-Tween-BSA). Specimen grids were then floated on droplets of gold-conjugated anti-FITC antibody at 1:20 dilution in PBS-Tween-BSA for 16 h at 4 °C, and washed with PBS-Tween-BSA (6 × 5 min) and PBS (3 × 5 min). Following post-fixation with 2% glutaraldehyde in PBS (*v*/*v*) for 5 min, and washing with PBS (2 × 5 min) and distilled H_2_O (5 × 2 min), the grids were air-dried and examined without additional staining on a JEOL JEM-1400 Transmission Electron Microscope. TEM images were collected with DigitalMicrograph Software v. 3.01 (Gatan, Pleasanton, CA, USA).

Flow cytometry population distribution analysis of fluorescent glycopolymer-bound bacteria within representative binding experiments was performed on a Beckman Coulter MoFlo XDP Cell Sorter, using Summit Software v5.2 for data acquisition and analysis (Beckman Coulter, Indianapolis, IN, USA). For each bacterial specimen, ~15,000 events were collected (debris and doublets excluded). Fluorescent bacteria were also collected for culture and follow-up flow cytometry analysis.

### 2.7. Isolation and Identification of Soluble Lectins LecA and LecB from P. aeruginosa

#### 2.7.1. Preparations of Presumptive LecA and LecB Fractions from *P. aeruginosa* Cultures

To ascertain whether *P. aeruginosa* lectins LecA (PA-IL) and LecB (PA-IIL) were elaborated by bacteria employed in these binding studies, agarose bead-bound monosaccharides were used to capture the soluble lectins from prepared specimens of representative CF sputum isolate CF-S 8314-1. Previously published *Pseudomonas* lectin isolation methods [93] guided our methods’ development. *P. aeruginosa* were cultured in M9 minimum media to stationary phase and cells collected by centrifugation (9000× *g*, 15 min). Cell pellets (~150 mg each) were disrupted by sonication (Heat Systems Ultrasonics W-385) in 1 mL of phosphate-buffered saline containing 1 mM CaCl_2_ (PBS-Ca). Alternatively, this isolate and other strains were harvested from nutrient agar plate culture and suspended in PBS-Ca prior to disruption. Sonicated specimens were then centrifuged (14,000× *g*, 10 min), and the supernatant containing soluble materials was collected. 

For harvesting of the galactose-binding protein LecA, 200 µL of α-D-galactose beads, pre-washed with PBS-Ca (1.5 mL × 3), was added to the prepared bacterial supernate. Following 1 h room temperature (RT) incubation with mixing, the beads were placed in a Spin-X centrifuge tube filter unit and gently centrifuged (500× *g*, 30 s). The beads and “first filtrate” were then processed separately as follows. The protein-bound α-D-galactose beads on the filter unit were rinsed with PBS-Ca (1.5 mL, 3 times). The LecA candidate protein was dissociated from the rinsed α-Gal beads by 1h RT incubation in a 25 mM melibiose (disaccharide D-galactose-α(1→6)-D-glucose) in PBS-Ca solution (with equivalent mass of the beads), then collected by centrifugation through the filter (14,000× *g*, 1 min).

For harvesting the mannose/fucose-binding protein LecB, 200 µL of pre-washed α-D-mannose beads were added to the “first filtrate” (obtained from the galactose bead extraction of the sonicated bacterial preparations). Following incubation as above, the solution of “first filtrate”-exposed beads was applied to a fresh filter unit, centrifuged, and rinsed with PBS-Ca. The LecB candidate protein was dissociated from the rinsed α-Man beads with 1 h at RT exposure to a saturated 4-nitrophenyl α-L-fucopyranoside solution in PBS-Ca, and collected by centrifugation as above. Presumptive lectin samples were compared with purchased *P. aeruginosa* lectin standards employing SDS-PAGE. Electrophoresis was performed on 4–12% Bis-Tris precast gels (Criterion XT, Bio-Rad Laboratories, Hercules, CA, USA) using a 2-(*N*-morpholino) ethanesulfonic acid running buffer (pH = 6.4) at a constant voltage of 200 V, and gels were stained with Coomassie blue. 

#### 2.7.2. Identification of LecA and LecB Proteins with Mass Spectrometry

Mass spectrometry was utilized to identify the *Pseudomonas* proteins eluted from the galactose- and mannose-agarose beads, with comparison to commercially available lectins. Following desalting and prior to analysis, samples were maintained in a protein solution of 10 μg/mL lysozyme and 10 μg/mL BSA in 0.1% formic acid, as employing these carrier proteins minimized the loss of hydrophobic lectins to sample vessels and instrument components. Positive-ion mass spectra of samples and standards were acquired with direct infusion into an LTQ Orbitrap XL Mass Spectrometer equipped with electrospray ionization (ESI) and Xcalibur software v2.1.0 (Thermo NA, Thermo Fisher Scientific, Waltham, MA, USA). Accurate mass spectra of multiply-charged species were deconvoluted using the Xtract feature of the software to determine molecular weights of the bead-extracted proteins and the authentic standards. Additional methodological details can be found in Supplemental File S4.

## 3. Results

### 3.1. P. aeruginosa Collection Heterogeneity in Source, Physiology, and Phenotypes

#### 3.1.1. *P. aeruginosa* Collection Character Overview

Toward considering whether an anti-infective therapy based on carbohydrate binding would be feasible for the spectrum of bacterial phenotypes observed from acute and chronic *P. aeruginosa* infections, specimens for this binding study were obtained from various CF and non-CF (NCF) sources. Assorted CF isolates were anticipated to reflect the range of characteristics expected of this opportunistic pathogen with varied and prolonged exposures to the human host and to therapeutic interventions. Strains from non-CF sources were included to more closely represent wild-type *P. aeruginosa*.

To begin this survey of the carbohydrate-binding profiles of CF isolates of *P. aeruginosa*, it was first important to assess the actual character of the collection of CF and non-CF specimens. This included detailing the specific characteristics of the individual clinical isolates and laboratory strains, particularly those that might affect glycopolymer binding. Several biological and biochemical tools were used to examine the collection diversity and the individual strain traits. The detailed data and methodologies are presented in Supplemental File S2. Highlights of the results are provided below.

#### 3.1.2. Physiological Diversity in This Collection of CF and Non-CF *P. aeruginosa*

All organisms in this collection were identified as *Pseudomonas aeruginosa* by automated microbial identification instrumentation with standardized proprietary algorithms. As presented in Supplemental File S2, Table S2.1, a review of the individual physiological assays revealed the flexibility of *P. aeruginosa* as a species, and illustrated the diversity within this collection. Of the 48 assays in the Gram-negative panel, 12 assays showed >10% of the cultures presenting with a positive or negative biochemical activity contrary to the norm for the species. CF isolates were positive less frequently for the majority of these features than NCF specimens. Of the CF isolates, mucoid cultures typically demonstrated these 12 activities less frequently than either nonmucoid CF or NCF specimens. A notable exception was lipase activity, for which 75% of CF mucoid *P. aeruginosa* (3 of 4) were positive for this activity, whereas only 22% of CF nonmucoid strains and 40% of NCF specimens were positive. Two CF isolates (one nonmucoid and one mucoid) were positive for trehalose utilization, while no NCF strains showed this activity. These data illustrate the physiological heterogeneity of the *P. aeruginosa* collection to be used in this survey of whole-cell carbohydrate binding.

#### 3.1.3. Phenotypic Diversity of This *P. aeruginosa* Collection

The phenotypic diversity among the specimens in this collection was also considerable, and is documented in Supplemental File S2. A greater variety was exhibited in plate cultures than can be captured by the standard phenotypic descriptions of motile (50%, *n* = 9), nonmotile nonmucoid (22%, *n* = 4), and mucoid (28%, *n* = 5). Photographic examples are presented in Supplemental File S2, Figure S2.1. In addition to colony morphology, the pigment and fluorescent compound elaboration on various media also varied within phenotypic groups, and between bacteria from NCF and CF sources, as detailed in Supplemental File S2, Table S2.2. For example, not all mucoid, or all nonmotile nonmucoid, or even all motile *Pseudomonas* were positive for pigment on *Pseudomonas* P Agar (media designed to enhance the production of *Pseudomonas aeruginosa*-specific pyocyanin).

The presence of the physical features of flagella and/or pili, which may be involved in carbohydrate binding, were also varied traits among this collection of bacteria. These data were determined via TEM imaging and are presented along with the specimens’ phenotypic data in Supplemental File S2, Table S2.2. Flagella were present for most cultures (89% F+), while pili were less frequently identified with only 78% pili-positive (P+). Twitching assays confirmed the presence of functional pili for the motile and nonmotile specimens noted as P+ by TEM. Twelve isolates (67%) displayed flagella and at least occasional pili by TEM. 

TEM analysis revealed that plate culture observations of *P. aeruginosa* motility, or the lack of motility, were not adequate to predict the presence or absence of flagella and/or pili. This is illustrated through comparing the isolate descriptors with the images of Supplemental File S2, Figure S2.2: (a) motile ATCC BAA47—flagella are apparent; (b) nonmotile mucoid ATCC 33468—flagella are absent; (c) nonmotile nonmucoid small colony variant (SCV) CF-S 3443—flagella are detected; and (d) with higher magnification of nonmotile nonmucoid CF-S 8314-1, both flagella and pili are observed. In other examples, the *Pseudomonas* isolate CF-T 3435 was motile on agarose plates and was flagella-positive (F+) by TEM (as illustrated previously [128]), while specimen NCF-S 3391 was motile yet flagella-negative (F−), presumably by the action of the numerous pili observed [128]. Of the nine nonmotile or mucoid isolates, all but one (mucoid ATCC 33468) were clearly F+.

As a collection, these *P. aeruginosa* present with the phenotypic, structural, and physiological diversity reflecting the heterogeneity typical of this opportunistic pathogen, and, as such, these data suggest that this panel, though small in number, is appropriate for use in the surveying of *P. aeruginosa* carbohydrate-binding trends.

### 3.2. PAA-Fluor Glycopolymers Were Amenable to Solution-Phase Bacterial Binding Studies

**Figure 1:**
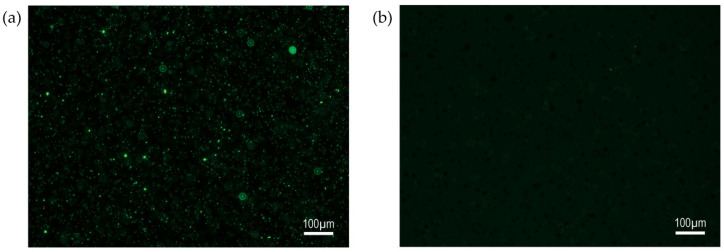
Use of polyacrylamide (PAA)-based multivalent fluorescent glycopolymers bearing pendant monosaccharides in bacterial binding assay provided the means to microscopically visualize and compare *Pseudomonas aeruginosa*-binding specificities and revealed anomeric configuration effects. Fluorescence micrographs of glycopolymer-binding results for *P. aeruginosa* cystic fibrosis sputum isolate CF-S 8314-1 with pendant monosaccharides β-GalNAc (**a**) and α-GalNAc (**b**) demonstrated preferential association with polymer possessing β anomer of *N*-acetylgalactosamine.

The investigation of the carbohydrate-binding patterns of this *P. aeruginosa* collection was performed using polyacrylamide (PAA)-based multivalent fluorescent glycopolymers, with the general scheme shown in Table 1, featuring the pendant monosaccharides depicted in Supplemental File S2, Figure S2.3. The use of water-soluble multivalent fluorescent PAA-based glycopolymers in solution-phase binding studies readily permitted the evaluation of carbohydrate-binding trends. Using fluorescence microscopy, glycopolymer-bound live whole-cell *P. aeruginosa* appeared as fluorescent objects with preferred and non-preferred sugar binding visually distinct, as illustrated in the micrographs of CF sputum isolate CF-S 8314-1 (Figure 1a and Figure 1b, respectively). The analysis of *P. aeruginosa* solution-phase binding reactions of β-GalNAc (Figure 1a) and α-GalNAc (Figure 1b) PAA-fluor glycopolymers in parallel revealed, for this isolate and others, that the organism associated with much greater prevalence to the glycopolymer with pendant monosaccharide β-GalNAc than the corresponding α-anomer α-GalNAc glycopolymer.

Bacterial-PAA-Fluor glycopolymer-binding reactions were also amenable to fluorescence intensity analyses in microtiter wells or with flow cytometry (described below).

### 3.3. P. aeruginosa Carbohydrate-Binding Profiles

**Figure 2:**
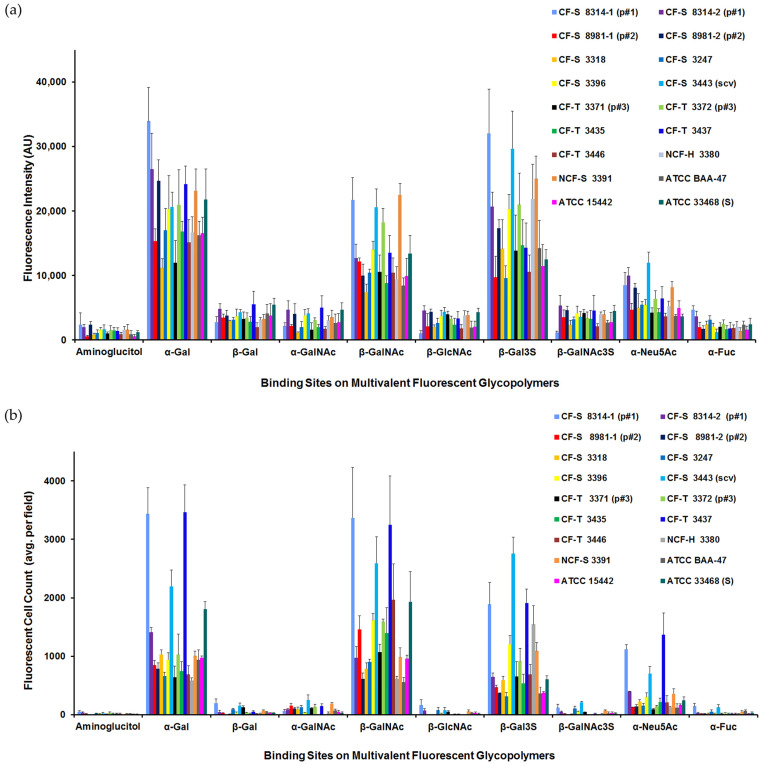
Carbohydrate-binding profiles of heterogeneous panel of clinical isolates and laboratory-adapted strains of *P. aeruginosa* reveal trends toward preferential adherence to multivalent glycopolymers possessing pendant monosaccharides α-Gal, β-GalNAc, and β-Gal3S, and, to a lesser extent, α-Neu5Ac. (**a**) Fluorescent PAA-based glycopolymer binding detected spectrofluorometrically for specified pendant monosaccharide-bound *P. aeruginosa* in solution, expressed as fluorescence intensities (AU, arbitrary units of fluorescence intensity). (**b**) Fluorescent glycopolymer-bound *P. aeruginosa* was evaluated microscopically, and expressed as average number of fluorescent cells per microscopic field obtained via automated object detection. Abbreviations for sources of specimens: CF, cystic fibrosis; CF-S, CF sputum culture, CF-T, CF throat culture; NCF-H, non-CF hip culture; NCF-S, non-CF sputum culture; ATCC, American Type Culture Collection; p#, pair #, where two distinct isolates collected from same specimen are considered a pair. Abbreviations for pendant monosaccharide on respective multivalent fluoresceinated glycopolymer: Gal, D-Galactose; GalNAc, D-*N*-Acetylgalactosamine; GlcNAc, D-*N*-Acetylglucosamine; Gal3S, D-Galactose-3-sulfate; GalNAc3S, D-*N*-Acetylgalactosamine-3-sulfate; Neu5Ac, D-*N*-Acetylneuraminic acid; Fuc, L-Fucose.

The quantitation of solution-phase PAA-Fluor glycopolymer-bound *P. aeruginosa* was performed via both multimode spectrometry and fluorescence microscopy. Quantitation permitted the normalization of experiments and relative comparisons between glycopolymers and among clinical and laboratory *P. aeruginosa* strains. The carbohydrate-binding profiles of these *P. aeruginosa* are depicted in Figure 2. The spectrofluorometric data are expressed as the fluorescence intensity (Arbitrary units, AU) of glycopolymer-bound bacterial suspensions in Figure 2a, and the microscopic data are plotted as average fluorescent glycopolymer-bound bacterial cell counts/field in Figure 2b. A select portion of these data is also included in a published conference abstract [129]. 

All eighteen *P. aeruginosa* showed significant binding to the glycopolymers specific for monosaccharides α-D-galactose (α-Gal), β-D-N-acetylgalactosamine (β-GalNAc), and β-D-galactose-3-sulfate (β-Gal3S). Several strains bound to α-D-N-acetylneuraminic acid (α-Neu5Ac) modestly, as evaluated by fluorescence intensity (Figure 2a), and three cultures showed significant binding to this glycopolymer by fluorescent cell counts (Figure 2b). The binding data for each strain of *P. aeruginosa* (as demonstrated in Figure 1 for CF-S 8314-1) showed that the anomeric configuration of the pendant monosaccharide was clearly determinative; i.e., α-Gal rather than β-Gal was preferred, and acetylated amino sugar β-GalNAc rather than α-GalNAc. With 3-O-sulfation, β-Gal3S was markedly preferred over β-Gal. In contrast, β-GalNAc3S-PAA-Fluor bound dramatically less than the non-sulfated galactosamine glycopolymer. Notably, there was little binding detected to the α-L-Fucose (α-Fuc) glycopolymer by any of the eighteen strains of *P. aeruginosa*, whether employing the commercially available α-Fuc-PAA-Fluor or the comparable glycopolymer synthesized and verified in-house.

In comparing the nine fluorescent glycopolymers and the control polymer, across the strains in Figure 2a, the PAA-Fluor glycopolymers were grouped into four *P. aeruginosa*-binding categories. Based on approximate ranges of the fluorescence intensity (as AU): Group I, with a range of 500–2400 AU, included only the negative control aminoglucitol; group II with a range of 1000–5600 AU, included β-Gal, α-GalNAc, β-GlcNAc, β-GalNAc3S, and α-Fuc; group III, 3600–12,000 AU, included only α-Neu5Ac; and group IV, 7400–34,000 AU, included α-Gal, β-GalNAc, and β-Gal3S. The ranges observed within each binding category illustrated the heterogeneity of the binding intensity between strains for individual PAA-Fluor glycopolymers, as well as within the *P. aeruginosa* PAA-Fluor glycopolymer-binding groups (I, II, III, and IV). 

*P. aeruginosa* carbohydrate-binding patterns quantitated with fluorescence microscopy (Figure 2b) paralleled the spectrofluorometric findings (Figure 2a). Very little fluorescence was seen to the aminoglucitol-PAA-Fluor control, with a range of only 5–54 fluorescent bacterial cells/field. The preferred glycopolymer bacterial binding, grouped as above, ranged from an average of ~100 to over 3000 fluorescent cells/field. As with the fluorescence intensity data, the heterogeneity of the binding was observed among the strains with regard to the positive binding of specific glycopolymers. This variability is illustrated in Supplemental File Figure S2.4 with an enlarged plot of the fluorescence microscopy data for α-gal-PAA-Fluor binding (as in Figure 2b). All *P. aeruginosa* specimens were clearly positive for α-gal-PAA binding by average fluorescent cell counts, with the binding ranging from ~600–3500 fluorescent cells/field.

To facilitate the investigations (presented below) of binding trends among CF- and non-CF-derived *P. aeruginosa*, the preferred multivalent fluorescent glycopolymers of groups III and IV above, those possessing α-Gal, β-GalNAc, β-Gal3S, or α-Neu5Ac were given the classification of “higher binding” for an individual isolate or strain when the binding data fluorescence intensity was >6000 AU or the average # fluorescent cells/field was >1000. 

### 3.4. Fluorescence Microscopy, TEM, and Flow Cytometry Reveal Subset of Population Was Responsible for the Positive Binding Characteristic

#### 3.4.1. Glycopolymer-Bound Populations Investigated via Microscopy

**Figure 3:**
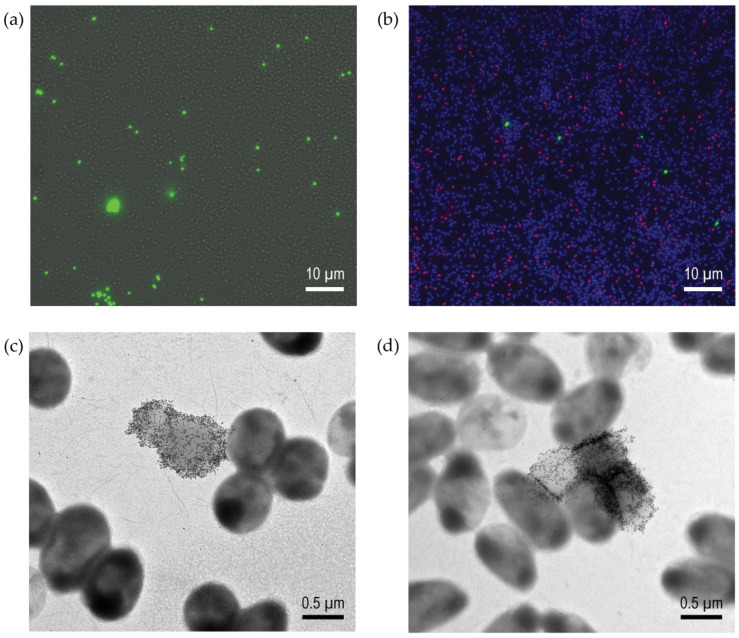
Fluorescence and transmission electron microscopy of positive fluorescent glycopolymer-*P. aeruginosa*-binding reactions indicate that, for each culture preparation, a small subpopulation was associated with the glycopolymer. (**a**) Overlay of brightfield and fluorescence images of *P. aeruginosa* isolate CF-S 8314-1 reaction with β-GalNAc glycopolymer. (**b**) Fluorescence image of ATCC BAA47 binding fluorescent β-GalNAc-glycopolymer, with supplemental staining by DAPI and PI. (**c**,**d**) Transmission electron micrographs of α-Gal-PAA-Fluor association with *P. aeruginosa* CF-S 8314-1 (**c**) and ATCC BAA47 (**d**), in specimens labeled post-binding assay with gold-conjugated anti-fluorescein antibody.

To further microscopically investigate the carbohydrate binding of bacterial cultures as populations, specimens from PAA-Fluor glycopolymer-binding experiments were viewed at a higher magnification, either by collecting and overlaying the fluorescence and brightfield images, or with additional staining by nuclear and dead cell stains. Both situations provided insight into the population binding characteristics, as shown in Figure 3a and Figure 3b, respectively. The analyses of fluorescence of the β-GalNAc-PAA-Fluor-bound preparations of both the CF sputum isolate CF-S 8314-1 overlaid with the brightfield image (Figure 3a), and of the ATCC laboratory strain BAA47 stained with DAPI and propidium iodide (Figure 3b), revealed that only a small percentage of the bacteria in each specific culture possessed bound glycopolymer (typically ~1% or less). This characteristic was observed for each glycopolymer test where the specific pendant monosaccharide–bacteria reaction demonstrated relatively high binding intensities via fluorescence spectrofluorometry. When the rinsing step to remove the unbound glycopolymer was reduced to a minimum, fluorescent glycopolymer-bound bacteria still represented only ~2% of the population. This is illustrated in Supplemental File S3 Figure S3.1 with the DAPI-stained image of the α-Gal-PAA-Fluor binding reaction with strain ATCC BAA47. 

A higher-resolution investigation of the localization of the bacteria-glycopolymer interactions was possible with TEM following the immunogold labeling of the glycopolymer-bound bacteria through the fluorescein moiety of the polymer. The TEM imaging of the α-gal-PAA-Fluor-bound strains CF-S 8314-1 (Figure 3c) and ATCC BAA47 (Figure 3d) showed intense immunogold labeling across the cell surface. Notably, this immunogold-labeled glycopolymer cell binding was limited to only a few cells within the positive binding preparations. With bacterial-polymer-binding reactions deemed negative by the fluorescence intensity and fluorescent cells counts, only occasional gold particles were observed when the comparable immuno-labeling for the FITC component of the PAA-Fluor was performed (as in Supplemental File S3, Figure S3.2). The TEM data are consistent with the fluorescence microscopy observations showing a limited number of bacteria providing the positive glycopolymer-binding result for the culture.

#### 3.4.2. Glycopolymer-Bound Populations Evaluated by Flow Cytometry

**Figure 4:**
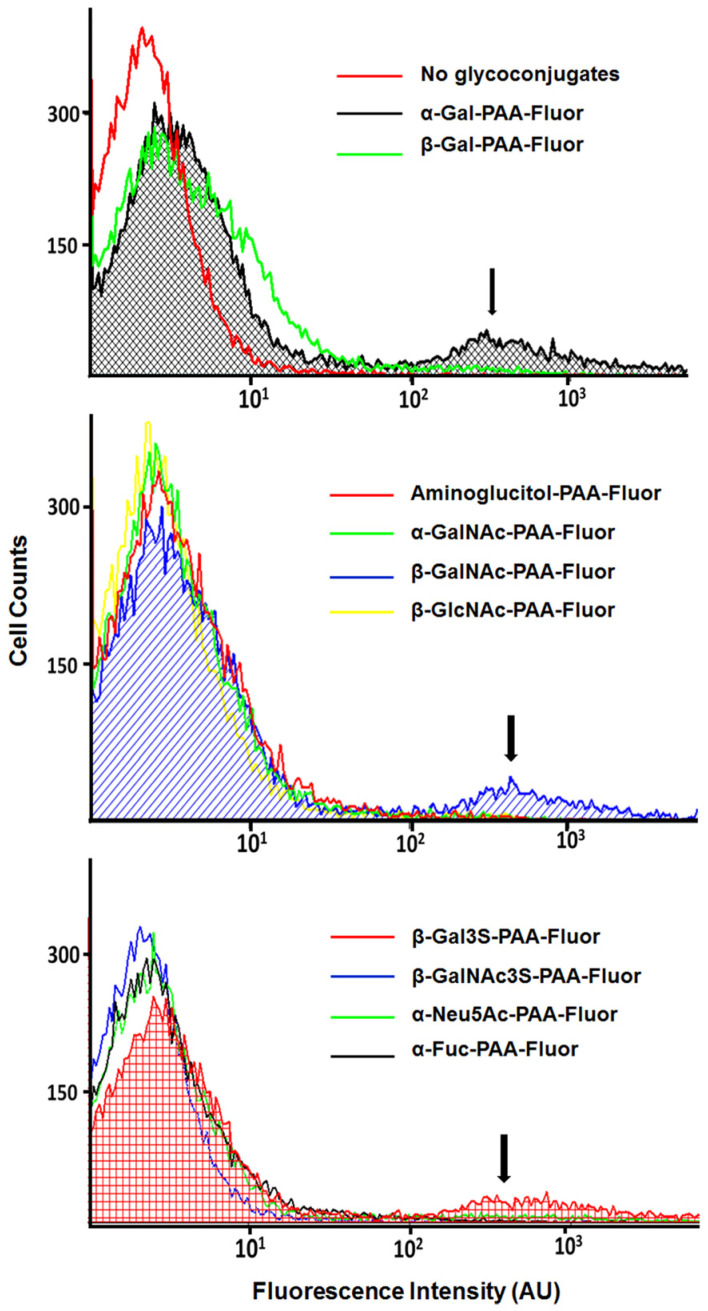
Flow cytometry population analysis depicting carbohydrate-binding profile of CF throat culture isolate *P. aeruginosa* CF-T 3435 illustrates that the preferred pendant carbohydrates are α-Gal, β-GalNAc, and β-Gal3S, and that only small subpopulations of those positive cultures possess high fluorescence intensity.

The flow cytometry analysis of representative bacterial-fluorescent glycopolymer preparations also provided evidence of pendant monosaccharide-binding profiles consistent with the data obtained with the multi-mode microplate reader, the fluorescence microscope, and the TEM. As seen in the flow cytometry data in Figure 4 for CF throat isolate CF-T 3435, high-intensity fluorescence indicative of binding was observed for a small percentage of the cells in binding reactions with PAA-Fluor glycopolymers possessing α-Gal-, β-GalNAc-, or β-Gal3S. Glycopolymers with pendant β-Gal, α-GalNAc, β-GlcNAc, β-GalNAc3S, α-Neu5Ac, or α-Fuc provided few to no cells with high-intensity fluorescence. These distribution data confirm the preferred glycopolymer profile seen by other methods for this isolate and support the observation that the binding signature of a culture population was provided by less than 2% of the population. When fluorescing glycopolymer-bound cells were collected and cultured, and the binding assays repeated with the newly grown cultures, again, only a small portion of the culture population showed significant fluorescent glycopolymer binding.

### 3.5. Varied Assay Conditions Did Not Change the P. aeruginosa Preferred Pendant Monosaccharide PAA-Fluor Glycopolymer-Binding Profile

**Figure 5:**
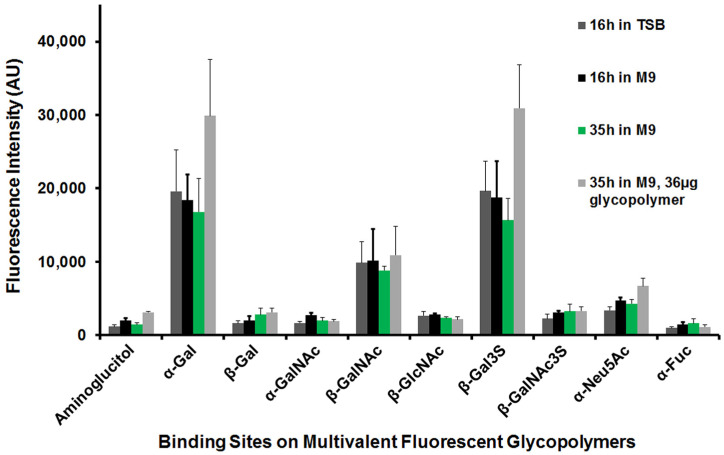
Carbohydrate-binding profiles of cystic fibrosis isolate CF-T 3435 *P. aeruginosa* evaluated with varied growth and assay conditions indicate consistent preferential adherence to specific multivalent glycopolymers possessing pendant monosaccharides α-Gal, β-GalNAc, and β-Gal3S. PAA-Fluor glycopolymer binding of CF-T 3435 was determined spectrofluorometrically, and expressed as fluorescence intensities, for four test conditions: 35 h in M9 (green bars), the routine assay as depicted in Figure 2a, with liquid culture for 35 h in minimal media (M9) supplemented with glucose, followed by rinse and exposure to 18 μg glycopolymer; 16 h in trypticase soy broth (TSB) or M9 prior to assays; and 35 h in M9 prior to reaction with 36 μg glycopolymer.

Using CF throat culture isolate CF-T 3435 as an example, the effects of variations in growth conditions on glycopolymer binding were investigated. The spectrofluorometric data are plotted in Figure 5. Culture incubation times of 16 h and 35 h for binding profiles from exponential and stationary phase cultures were compared, as well as the type of culture media, i.e., minimal media (M9 with glucose) and rich media (TSB). Additionally tested was the effect of doubling the amount of glycopolymer employed relative to that of the standard binding assay conditions (i.e., 36 μg of each respective PAA-fluor glycopolymer rather than the standard 18 μg in the M9 35 h reactions). The media type and incubation time did not alter the binding profiles significantly compared to the routine assay conditions (plotted in green) for this isolate. The preferred pendant monosaccharides were α-Gal, β-GalNAc, and β-Gal3S regardless of growth condition. Doubling the glycopolymer concentration in the binding reactions did increase the fluorescence intensities greater than 50% for glycopolymers with α-gal, β-gal3S, and the polymer control aminoglucitol.

Comparable increases were not observed for the other pendant monosaccharide-possessing polymers. After subtracting the increased background signal, the fluorescence intensities of the α-Gal and β-Gal3S polymer bacterial binding reactions still reflected a greater than 50% increase with the 2X polymer. This suggests two possibilities: that, for these two sugars, the binding sites may not be fully saturated with the 1X polymer reactions; or that the character of these specific glycopolymers may enhance aggregation (or association), resulting in higher fluorescence intensities in binding reaction mixtures when more glycopolymer is present. Notably, no significant binding of CF-T 3435 to α-L-fucose-PAA-Fluor was observed for any of these growth or assay conditions. Laboratory strain BAA-47 challenged with additional fucose glycopolymer also did not yield significant increases in detectible glycopolymer-bacteria binding.

### 3.6. Lectins LecA (PA-IL) and LecB (PA-IIL) Were Present in Binding Assay Cell Preparations of CF Sputum-Derived P. aeruginosa

The *P. aeruginosa* lectins LecA and LecB have long been known to have an affinity for galactose and fucose, respectively [47,93], as introduced earlier in this text. In the current report of solution-phase whole-cell *P. aeruginosa* carbohydrate-binding profiles, the α-Gal-PAA Fluor demonstrated significant binding for all strains, as predicted for the surface-expressed LecA, whereas the α-Fuc glycopolymer did not. To discern whether the lack of α-L-fucose-PAA-Fluor binding detected for this panel of bacteria might reflect a lack of production of the fucose-binding lectin LecB under the growth conditions used in these studies, prototypical CF isolate CF-S 8314-1 and laboratory-standard ATCC BAA47 were evaluated for the presence of both common soluble *Pseudomonas* lectins LecA and LecB. The SDS-PAGE of proteins isolated from sonicated bacterial cell culture pellets of both strains affinity-purified with agarose-immobilized galactose and mannose (as detailed in Supplemental File S4) weakly revealed Coomassie blue staining bands with electrophoretic migrations comparable to one another and to commercial standards for *P. aeruginosa* lectins LecA and LecB. Similar results were obtained with affinity-purified protein preparations from additional culture sources, including CF-S 8314-1 in M9 broth and on nutrient agar, BAA47 in M9 broth, and CF-S 3318 in trypticase soy broth.

Mass spectrometry confirmed the lectin identities [95] of the affinity-purified proteins. High-resolution positive-ion electrospray mass spectrometry of the desalted purified proteins and authentic standards yielded abundant +8 multiply-charged species. The deconvoluted spectra verified that the molecular masses of the CF isolate-derived proteins were the same as those of the respective authentic *P. aeruginosa* lectin standards LecA 12,721 Da and LecB 11,732 Da. The mass spectral data confirming the presence of lectins LecA and LecB in representative CF-S 8314-1 prepared from the M9 culture under the binding experiment growth and harvest conditions are presented in Supplemental File S4, Figure S4 and Table S4.

### 3.7. P. aeruginosa Source, Phenotype, and Structural Features Did Not Clearly Predict Enhanced Glycopolymer-Binding Profiles 

#### 3.7.1. Overview of *P. aeruginosa* “Higher Binding” Glycopolymers Status 

To review, as seen above in Figure 2a with fluorescence spectrometry, all *P. aeruginosa* in this study shared the same general carbohydrate-binding profile, showing significant interactions with PAA-Fluor polymers possessing pendant monosaccharides α-Gal, β-GalNAc, or β-Gal3S, with intensities of >6000 AU. The fluorescence intensities among the strains, however, did vary. Clinical isolates #1, #8, #10, and #15, for example, relative to the remaining specimens, had markedly higher-intensity readings of >15,000 AU for each of these three glycopolymers. The individual strain differences in the glycopolymer-bound were even more evident when evaluated microscopically, as illustrated in Figure 2b. By cell counts, *Pseudomonas* cultures were classified as “high binders” of a specific glycopolymer when an average of >1000 fluorescent cells/field were observed. 

The three strains that showed the greatest binding of glycopolymers via fluorescence microscopy were CF isolates CF-S 8314-1, CF-S 3443, and CF-T 3437 (plot IDs #1, #8, and #12, respectively). Plate culture traits and structural features varied among these *P. aeruginosa*. By plot IDs: #1 was nonmucoid nonmotile with very blue-green colonies; #8 presented as small colony variant colonies with no discernable motility; and #12 was very motile. An analysis via the negative-staining TEM of each of these specimens revealed one polar flagellum per cell and occasional distinctive pili within the population. These data suggested that the relationships of the colony morphologies and the F+P+ phenotype to glycopolymer binding merited further investigation.

#### 3.7.2. Considerations of Source, Phenotype, and Structural Features of *P. aeruginosa* for Contribution to Glycopolymer “High Binding” Status

As characteristics of the individual strains may contribute to enhanced carbohydrate binding, Table 3 includes the source, colony phenotype, and structural feature(s) observed, along with the preferred glycopolymer microscopic and spectrofluorometric findings. To aid in identifying correlations based on the source, glycopolymer higher binding, and/or phenotype, specimen data sorted by and presented in these categories are included in Supplemental File S5.

Considering the traits of *P. aeruginosa* related to their originating sources, Supplemental File S5, Table S5.1 provides a convenient overview. Fifteen of the eighteen strains were of respiratory origin, with eight from CF sputa, five from CF throat cultures, and two from non-CF (NCF) sputa. The plate culture phenotypes of these 15 were as follows: 4 nonmucoid nonmotile, 5 mucoid, and 6 motile. Flagella were common to respiratory specimens, with the presentations of flagella and/or pili described as F+P+ (10), F+P− (3), and F−P+ (2, both from NCF sputa). The three non-respiratory *P. aeruginosa* were motile and either F+P+ (2) or F+P− (1).

Sorted by the preferred glycopolymer binding, Supplemental File S5, Table S5.2 describes the frequencies the glycopolymers were preferred by the strains in the collection, relative to the *P. aeruginosa* source, phenotype, and structural feature(s). The percentages of the strains reported as “high binders” of specific glycopolymers via cell counts (>1000 fluorescent cells/field) were as follows: β-GalNAc, 56% of the collection (10, all respiratory); α-Gal, 44% (8, all respiratory); β-Gal3S, 33% (5 of 6 respiratory, 1 NCF-hip); and α-Neu5Ac 11% (2, both CF sputa). Notably, the NCF hip specimen NCF-H 3380 with β-Gal3S as the preferred pendant monosaccharide was the only non-respiratory specimen to show high binding via cell counts to any glycopolymer. Two CF sputa also lacked a high-binding glycopolymer status by fluorescence microscopy.Focusing on the CF respiratory cultures, 100% of CF throat specimens showed high β-GalNAc-PAA-Fluor binding via fluorescence microscopy, whereas only 50% of CF sputa isolates shared this attribute. This collection included three CF culture pairs of a nonmotile nonmucoid isolate and a mucoid isolate obtained from the same respiratory specimen (pair and plot #IDs: pair 1, #1 and #2; pair 2, #3 and #4; and pair 3, #9 and #10). No preferred glycopolymer-binding profile trend was observed among paired isolates or through a comparison of the three nonmucoid versus the three mucoid specimens in these pairs.

Regarding the structural features, the F+ cultures were clearly positive by TEM with many cells in the population displaying a single polar flagellum. Pili were more difficult to detect by TEM and were observed less frequently than flagella on the cells in P+ cultures. While >60% of the high-binding strains were F+P+, specific glycopolymer binding did not correlate with the flagella presence or absence. Six of the sixteen F+ isolates showed high binding with α-Gal-PAA-Fluor, and as did the two F− strains (#15, #18). Structural combinations of F+P− and F−P+ were less frequent among high-binding strains (i.e., 10–25% depending on glycopolymer). Pili presence did not appear to predict the frequency of high binding to any or all of the four specific “high binder” glycopolymers (to include the glycopolymer with pendant sialic acid). The number of higher-binding glycopolymers observed per P+ strain varied. The number of P+ strains (of 14) with each glycopolymer high-binding profile is as follows: no “high binding (3), any 1 of the 4 glycopolymers (4), various combinations of 2 of the 4 (4), 3 of the 4 (1), or all 4 (2). For the four F+P− strains (#2, #3, #9, and #16), higher binding occurred once for α-Gal and twice for β-GalNAc. “High binding” to the α-Neu5Ac glycopolymer was only seen for two CF isolates (as noted above) and both were F+P+.

Probing the data for phenotype-glycopolymer-binding correlations, Supplemental File S5, Table S5.3 presents the 18 *P. aeruginosa* strains and their high-binding glycopolymer profiles organized by colony phenotypes. Notably, 100% of nonmucoid isolates (four, all CF) bound β-GalNAc at >1000 cells per field, i.e., enhanced, “high binding”. Only 60% of mucoid (three, two CF), and 33% of motile specimens (three, all CF) bound β-GalNAc at >1000 cells/field. By phenotype, nonmucoid, mucoid, and motile specimens showed enhanced binding to PAA-Fluors with the specific pendent monosaccharides as follows: nonmucoid to α-Gal (50%), β-GalNAc (100%), β-Gal3S (25%), and α-Neu5Ac (25%); mucoid to α-Gal (60%), β-GalNAc (60%), β-Gal3S (20%), and α-Neu5Ac (0%); and motile to α-Gal (33%), β-GalNAc (33%), β-Gal3S (33%), and α Neu5Ac (11%). The enhanced binding to one glycopolymer did not parallel or predict the comparable binding for another glycopolymer. Flagella and/or pili status also did not trend with a specific colony phenotype.

#### 3.7.3. Summary of *P. aeruginosa* Attributes and Enhanced Carbohydrate-Binding Profiles

The highlights of the findings in Table 3 are summarized as follows: (1) glycopolymers with pendant monosaccharides α-Gal, β-GalNAc, and β-Gal3S were bound by all strains tested; (2) collectively, respiratory specimens showed greater glycopolymer “high binding” patterns by fluorescent cell counts than non-respiratory strains; (3) β-GalNAc-PAA-Fluor appeared most frequently in the highly bound category, with all CF throat specimens and all nonmotile nonmucoid CF sputum isolates showing enhanced binding to β-GalNAc-PAA-Fluor; and (4) the presence of flagella, pili, or both did not guarantee plate motility or predict enhanced binding trends toward any specific multivalent glycopolymer.

The *P. aeruginosa* collection expressed much heterogeneity in features that might contribute to enhanced glycopolymer binding. To review strain attributes with binding patterns by glycopolymer: high binders with β-GalNAc (10), 7 F+P+, 2 F+P−, 1 F−P+, of mixed phenotypes motile (3), nonmucoid (4), mucoid (3); α-Gal (8), 5 F+P+, 1 F+P−, 2 F−P+, of mixed phenotypes of motile (3), nonmucoid (2), mucoid (3); and β-Gal3S (6), 5 F+P+, 1 F−P+, also with mixed phenotypes of motile (3), nonmucoid (2) and mucoid (1). Overall, the higher binding of one glycopolymer did not parallel the higher binding to another.

## 4. Discussion

### 4.1. Importance of Investigating the Characteristics Diversity of P. aeruginosa in Collection for Representative Data to Inform Clinically Relevant Carbohydrate-Binding-Based Therapeutic Strategies

This study provides background to inform potential anti-adhesion anti-infective treatment strategies aiming to use small carbohydrates or other monosaccharide-based derivatives (glycomimics/glycomimetics) for the interference of *P. aeruginosa*–carbohydrate interactions within the CF host airway. Such approaches in the context of the complex CF milieu are likely to address host–pathogen interactions, bacterial aggregation, biofilm matrix development and disruption, and the novel directed-targeting of antibiotics to the CF airway *P. aeruginosa*. It is well-established that *P. aeruginosa* readily adapt to their environment and express a wide variety of phenotypes, not only in the CF population, but also over time and location in the respiratory tract of an individual, as presented in the introduction and discussed in more detail below [6,10,11,13,15,16,17,19,20,21,28,29,31,33,41,44,117,118,130]. The heterogeneity of feature characteristics was, therefore, desired within the collection of *P. aeruginosa* employed so that the binding data would reflect the wide range of *Pseudomonas* presentations that treatment strategies would hope to address.

To attempt to capture this diversity in the current carbohydrate-binding patterns survey, we used reference strains, isolates from CF sputum and throat cultures, and specimens from non-CF respiratory and wound cultures. This work complements the many adhesion-related studies that employ reference strains and their derivatives to identify specific interactions. We evaluated the characteristics displayed by each *P. aeruginosa* isolate/strain. We looked for distinctions in presenting phenotypes and sources that might predict enhanced binding with specific glycopolymers, and thereby affect success of therapeutics mimicking the specific monosaccharides.

*P. aeruginosa* phenotypes from agar plates included the variety expected from CF airway specimens including the variability in pigments, fluorescence, motility, mature colony morphologies, and pearlescent extracellular matrix [6,13,14,16,18,28,29,44,131]. The collection included strains with wild-type-like motility and isolates showing host-adapted characteristics such as nonmotile nonmucoid colonies, mucoid colonies, and one small colony variant. Mucoid *P. aeruginosa* were primarily isolated from CF sputa, though one mucoid isolate came from a CF throat specimen and one ATCC bank isolate was from the sputum of an elder not tested for CF [124]. As laboratory culture conditions removed from the in vivo environment may detract from an accurate representation of the pathogen’s features when the specimen was taken [33,64,132,133], care was taken not to passage the clinical specimens in the laboratory extensively, and to verify the phenotypes prior to the binding assays. We found, as others have [131], that agar plate morphotypes did not accurately predict the presence or absence of the potential carbohydrate adhesins of flagella and pili. A TEM examination revealed that the “lack of motility” phenotype on solid media did not mean a lack of flagella or pili, as all but two strains were flagella-positive. Pili, as noted earlier, were more difficult to image. The twitching assays did confirm the presence of pili for those strains determined to be pili-positive + or weakly (infrequently) pili-positive (+) by TEM. As the frequency and number of pili on cells within a population were difficult to assess at the EM level, care is advised when evaluating pili data for the relative contribution or lack of contribution of pili to carbohydrate binding.

The metabolic flexibility characteristic of *P. aeruginosa* [6,14,20,21,31,42,44,64,126,134] was also observed in this collection. Via an automated panel of physiological assays for Gram-negative bacteria, mucoid *Pseudomonas* tended to qualitatively exhibit fewer substrate-positive enzymatic activities than did nonmucoid CF or non-CF strains. The quantitation of enzymatic activities was outside the scope of this carbohydrate-binding survey. This apparent loss of metabolic- and virulence-related activities of mucoid *P. aeruginosa* has been observed by others as well [11,134]. It has been suggested that such loss of function mutations may be indicative of chronic infection and more severe lung disease [13,19,28,34,131,135]. The current study was blind to whether the respiratory specimens arose from acute, chronic, and/or polymicrobial infections.

The diversity of phenotypic and metabolic characteristics among these *P. aeruginosa* strains suggested that this collection was suitable for the survey of whole-cell monosaccharide-binding profiles toward discerning binding trends anticipated among *P. aeruginosa* clinically.

### 4.2. Additional Factors Potentially Influencing P. aeruginosa Carbohydrate Binding In Vivo

It should also be respected in therapeutics design that, as discussed above, the varied permutations of the condition of the host, as well as the adaptations of the colonizing microbes and the treatments being received, are likely to keep the infectious landscape ever-changing [8,20,43,133]. The *P. aeruginosa* variability and fluidity in the expression or lack of expression of virulence features and structures are expected to complicate the development of targeted agents [10,11,15,20,24,26,29,36,58,63,88,90,116,117,131,136]. The distinctions and differences in complex mucosal glycoconjugates are anticipated in CF respiratory tracts and are mechanistically important to bacterial binding and/or clearance [9,60,65,67,78,79,81,82]. Sub-inhibitory concentrations of antibiotics may also elicit changes in *P. aeruginosa* that affect mucin binding [137,138] and the production of virulence factors, such as the cytotoxic phenazine pyocyanin [139]. Pyocyanin at non-lethal levels may increase the airway epithelial cell production of specific types of sialyl-Lewis antigens, such as sialyl-LeX, shown to be important to bacterial binding [67]. The increased cytotoxicity observed in vitro for the CF therapeutic agent colistimethate [48] when coincident with pyocyanin [140] suggests that inadvertent damage to the CF epithelium during antimicrobial treatment may also set the remodeling stage and provide even more sites for binding, aggregation, and biofilm establishment.

Additional details and discussion on characteristics of the CF airway and *P. aeruginosa* which may affect *P. aeruginosa* survival and the success of adjunctive anti-adhesive therapeutics can be found in Supplemental File S6.

### 4.3. Fluorescent Polyacrylamide-Based Glycopolymers with Pendant Monosaccharides as Convenient Tools for Surveying Whole-Cell P. aeruginosa Carbohydrate Binding

In this study, water-soluble fluorescent polyacrylamide-based (PAA-Fluor) glycopolymers presenting monosaccharides mimicking those typically found at the termini of human airway glycoconjugates [49] were used successfully to both visualize and quantitatively compare carbohydrate-binding patterns of a variety of *P. aeruginosa*. As noted earlier, select data using this approach were presented and published as a cystic fibrosis conference abstract [129]. As water-soluble compounds, PAA-Fluor glycopolymers have been readily employed in solution-based assays as demonstrated earlier with flow cytometry studies of bacterial binding [65,75,81]. When synthesized in controlled reversible addition–fragmentation chain-transfer (RAFT) polymerization reactions, PAA-glycopolymers provide well-defined, predictably uniform glycopolymers with pendant monosaccharides of anomeric specificity important to and illustrated by bacterial-lectin-binding investigations [22,54,119].

In the current study, with the anomeric configuration (i.e., α- or β-) of each test monosaccharide locked in place on short spacer molecules and ~20 residues of the monosaccharide per fluorescent-tagged polymer molecule, these reagents provided sufficient and reproducible binding and fluorescence for the desired comparisons. Fluorescence microscopy and fluorescence spectrofluorometry allowed for the quantitation of whole bacterial cell-glycopolymer binding in aqueous solutions across binding conditions, strains, and carbohydrates. As solution-phase assays, the binding was not dependent on, or affected by, the character or composition of a solid binding surface. Consequently, the assays also did not address the biofilm lifestyle directly. A visualization of rinsed PAA-Fluor bound bacteria with light, fluorescence, and electron microscopy, combined with flow cytometry, provided insights into the population-related binding characteristics. The microwell formats of the microscopy slides and the microtiter plates permitted convenient direct visual comparisons of the PAA-Fluor glycopolymers of the different carbohydrate configurations from the same bacterial culture preparations, with quantitation both microscopically via software-automated fluorescent cell counts and spectrofluorometrically as the population fluorescence intensities.

### 4.4. Insights Gained from Imaging and Quantitation of P. aeruginosa Carbohydrate-Binding Profiles via Multiple Modalities

The carbohydrate-binding profiles obtained for this panel of *P. aeruginosa* were relatively uniform with the preferential adherence of each strain to multivalent glycopolymers possessing pendant monosaccharides α-Gal, ß-GalNAc, or ß-Gal3S. The anomeric configuration was determinative. With the α- and ß- anomeric configurations of the test pendant monosaccharides locked in place, the data revealed that whole-cell *P. aeruginosa* binding was very specific, preferring α-Gal and not ß-Gal unless it was sulfated as ß-Gal3S; and ß-GalNAc and not the α-GalNAc or the sulfated ß-GalNAc3S. Portions of these observations were reported in a recent cystic fibrosis conference abstract [141].

The bacteria-glycopolymer-binding profiles were consistent between investigational modalities. Strain data obtained via fluorescence microscopy and fluorescent spectrofluorometry were paralleled, with positive binding reactions clearly distinct from negative reactions. A microscopic evaluation showed additional binding attributes that emphasized the value of using multiple modes of analysis. In each case, a well-dispersed small portion of the bacterial cells was responsible for the positive fluorescence signature of a culture. Flow cytometry confirmed the subset positivity. The fluorescence observed microscopically as cell-bound glycopolymers also suggested significantly greater numbers of receptors per positive cell than would be expected if the associations were via structural features like flagella and/or pili. The TEM imaging of two representative *P. aeruginosa* strains (one nonmotile nonmucoid CF isolate, one wild-type ATCC reference strain), following the immunogold labeling of the fluorescein moieties of α-Gal glycopolymer-positive reactions, showed the intense labeling of the cell surface of a few bacteria within the fields. This agreed with observations by fluorescence microscopy indicating that the α-Gal-PAA-Fluor glycopolymer, for a subset of the population, was covering the cell surface and not lighting up the flagella or matrix. The concept of subpopulations providing the desired function for the whole population is a familiar theme for micro-organisms [1,64,132,142,143], and undoubtedly contributes to species survival and the chronic colonization of the vulnerable host [1,8,11,21,42,64,132,142,143,144]. Pili were not readily seen with TEM after the fluorescein-immunogold labeling reactions. As such, alternate methods specific for pili preservation would be required for adequately assessing whole *P. aeruginosa* cells for pili–glycopolymer interactions. Regarding the notable lack of immunogold labeling of the matrix between the cells, this study was not designed to create a traditional biofilm, so no data about carbohydrate binding within a biofilm could be extrapolated from these findings.

The α-galactose moiety binding observed for a subpopulation of the cells in the cultures is consistent with the natural ligand-binding affinity [88,94,112] anticipated for cells presenting surface-associated LecA. Whole *Pseudomonas* cells lacked binding with the ß-Gal-PAA-Fluor, representing the lesser preferred LecA ligand ß-Gal [112]. This finding may be reflective of previous observations that, within the LecA tetramer, three of the four monomers have a high affinity for the α-anomer, while only one monomer binds the ß-Gal configuration [96]. Early reports of *Pseudomonas*-binding glycoconjugates possessing terminal ß-galactose may imply that, when this anomeric configuration is presented in the context of larger oligosaccharides [65,72,78,145], it may override the natural LecA ligand preference. As explained for other lectins, the LecA grove may have sites available for making contacts with the other carbohydrates in the chain or branch to improve the apparent affinity for glycoconjugates with terminal ß-galactose [91,108,111,145]. LecA activities were known, from hemagglutination assays and other studies, to be inhibited by α-Gal and, to a lesser degree, by ß-GalNAc [47,60,70,88]. This suggests that, in the current study, LecA might have contributed to the surface binding of both the α-Gal and the ß-GalNAc glycopolymers.

Unanticipated in the current study was the notable lack of binding of the fucose glycopolymer to any of the whole-cell CF or non-CF *Pseudomonas* isolates. Fucose glycopolymer-binding would be predicted through LecB [88,90,146]. Soluble LecB has a monosaccharide affinity specific for fucose and mannose and was recently shown to bind the α-mannose side chains of the Psl exopolysaccharide [89]. *P. aeruginosa* LecB has been reported as present in the extracellular matrix [89,90,102] and in other situations localized at the cell surface through the association with an outer membrane porin OprF [47,89,92,102]. For the culture conditions employed for this carbohydrate-binding survey, the whole-cell presence of both LecA and LecB proteins were confirmed, yet it was not determined if the lectins were located intracellularly and/or were outer-membrane-bound. If conditions did not permit LecB transport to the cell surface (i.e., lack of N-glycosylation) [92,115], the lectin-glycopolymer-binding would not be expected. While the liquid cultures in the current study were permitted to grow to the stationary phase (favorable for lectin production), the conditions were not designed to specifically generate biofilm networks. This is important to consider, in that much of the functional role of LecB is thought to be related to biofilm development and structural integrity [89,90,92,104]. As such, LecB in this *P. aeruginosa* survey may have remained cytosolic as seen by others for planktonic cells [146], or have been produced and released from the cells (free or in extracellular vesicles) prior to the exposure to glycopolymer. With no trapping matrix established, the extracellular LecB may have been removed with the cell rinses before the binding analyses were performed. Either way, any LecB produced that could have bound to the fluorescent fucose glycopolymer would not have been detected, leading to the presumption that the α-Fuc-PAA-Fluor glycopolymer did not bind *Pseudomonas* cells. Alternatively, if LecB proteins were extracellularly membrane-bound prior to these assays, the nature of the experiments may have contributed to the lack of detection of positive *Pseudomonas* binding to the α-Fuc-PAA-Fluor. This hypothesis is based on previous evidence that fucose-presenting glycopeptide dendrimers could dissociate LecB-based biofilm interactions [104], and that fucose itself could release outer-membrane-bound LecB [90] from its association with OprF (OMP F) [92]. We surmise that, upon binding, the fucose glycopolymer whole-cell LecB may have been liberated from the bacterial outer membrane and/or any extracellular matrix which existed and, subsequently, prior to detection, been removed with the rinses to remove the unbound glycopolymer. Additionally, as we suggest above for ß-Gal glycopolymers and as was seen in early studies of LecB inhibition with various blood group active substances [91], the whole-cell *P. aeruginosa* binding of fucose via LecB and calcium may also be more favored and more detectable when fucose is part of a more complex carbohydrate or carbohydrate–amino acid/peptide structural arrangement [9,91,103,111] than when presented as a monosaccharide in this binding survey.

Sialic acid, as represented here by the α-Neu5Ac-PAA-Fluor, associated with only a few *P. aeruginosa* isolates and with no discernable feature(s) correlating with the binding. Like ß-galactose and fucose, presentations of sialic acids in vivo within larger epitopes may provide more sites to interact with adhesins of whole-cell *Pseudomonas*, especially when glycoforms also possess fucose, as suggested by reports of *P. aeruginosa* binding sialyl-Lewis antigens on mucins and blood group active glycosphingolipids [9,65,66,81,91,147]. In contrast and, alternatively, depending on in vivo conditions, the in vitro work suggests that the removal of sialic acids from larger structures like the gangliosides GM1 and GM2, or the undersialylation of such carbohydrates on the apical membranes of CF cells, may enhance *Pseudomonas* binding, presumably by exposing the ß-galactose and *N*-acetylated ß-galactosamine epitopes [9,78,79]. Evidence of increased amounts of asialoGM1 on regenerating CF respiratory epithelia in culture that also show greater *P. aeruginosa* adherence than non-CF epithelial cells [148] supports the premise that sialyl residues at cell surfaces may block potential carbohydrate-binding sites for at least some of the *Pseudomonas* in the respiratory tract which make it past the mucociliary clearance components.

Interestingly, the sulfate ester possessing ß-Gal3S-PAA-Fluor associated with all the *Pseudomonas* tested. Earlier studies with neoglycolipids presenting 3-sulfo- or 6-sulfo-sialyl-Le^X^ antigens, likewise, saw *Pseudomonas* binding [81]. In the case of the galactose-3-sulfate, binding may reflect favorable calcium co-ordination with the sulfate ester in the *Pseudomonas* lectin-binding groves. In contrast, while the ß-GalNAc glycopolymer bound whole cells well, much less association was observed with the sulfate ester possessing ß-GalNAc3S, suggesting a lesser preferred docking when sulfate is present on the GalNAc. As the binding of both LecA and LecB to the respective preferred carbohydrates are known to involve calcium [47,96,97,146,147], calcium was included in all binding solutions in this study. Extracellular calcium in vivo, however, may be expected to both contribute to pathogen lectin interactions with host airway glycans and reduce bacterial clearance by increasing the viscosity of the mucus through cross-links of charged mucins, alginate, and DNA [2]. With the 3-*O*-sulfation of galactose being common to CF and non-CF acidic tracheobronchial mucin oligosaccharides [49,83,84,149], the current binding data suggest that such sulfation may provide yet another adhesive opportunity that favors *Pseudomonas* adherence and colonization in the CF airway.

ß-GalNAc glycopolymer binding was common to whole-cell *P. aeruginosa* in this study and may reflect the interaction of this pendant monosaccharide with type IV pili as pili are suggested in ganglioside studies as a common binding element [71,73,77] and/or with the cell surface CdrA thought to play a role in biofilm integrity [22,23,136]. Pili were difficult to detect by TEM in this binding study and few per cell were identified. With the significant level of binding observed for the ß-GalNAc glycopolymer at the exterior of *P. aeruginosa*, the association was more likely via an outer membrane protein. The suggestion of CdrA as a potential ß-GalNAc-binding partner is based on observations from recent studies [24,136]. CdrA was found to be associated with biofilm matrix materials Psl and Pel [24,136] and was detected in a variety of clinical and environmental *P. aeruginosa* specimens [136]. CdrA appeared both released from the cells and tethered to the outer membrane via the outer membrane pore CdrB [89,136]. More specifically, CdrA was shown to bind both the neutral mannose-rich exopolysaccharide Psl (like LecB) and the cationic pellicle exopolysaccharide Pel [112,132]. The Pel matrix in CF was reported as composed primarily of GlcNAc and GalNAc and to vary in degree of deacetylation and, thereby, charge [24,136]. If the ß-GalNAc-glycopolymer binding observed in the current study was via cell surface CdrA, this would be consistent with the suggestion that in vivo extended fimbrial structures may interact with GalNAc in the biofilm exopolysaccharide Pel to provide a stabilizing point for bacterial cells in the matrix. As both the Psl and the Pel biofilm exopolysaccharides have been detected in CF sputa [24], this potential CdrA-GalNAc association could be of clinical significance. From the whole-cell carbohydrate-binding profile findings of the current study, additional tests are recommended to test the hypothesis that multiple LecA α-gal and CdrA β-GalNAc interactions at the cell surface of intact *P. aeruginosa* may act as bridges to the webs of Psl and Pel exopolysaccharides and extracellular lectins important to the biofilm community integrity.

The quantitation of microscopically fluorescent glycopolymer-bound cells also permitted surveying for trends in enhanced *P. aeruginosa* binding, and revealed what was classified as “higher binding” for some bacterial strain-carbohydrate pairs. The ß-GalNAc glycopolymer was considered “higher binding” for 56% of the collection, notably, all of which were of respiratory origin. The α-gal was “higher binding” for 44% of the *Pseudomonas*, also all from respiratory sources. For a given strain, the high binding level of one glycopolymer did not necessarily correlate with the high binding of the other. An important trend to note is that 100% of the nonmucoid nonmotile CF sputum isolates and 100% of CF throat isolates showed this enhanced binding to the ß-GalNAc-PAA-Fluor. This finding is consistent with early laboratory observations of the binding of numerous pulmonary pathogens to ß-GalNAc presented in glycolipids as internal or terminal GalNAc(ß1-4)Gal sequences [77,145]. Only one non-respiratory specimen, a non-CF hip isolate, achieved the “higher binding” level and only for the ß-Gal3S glycopolymer. These survey data portray cell-associated carbohydrate-binding adhesins as abundantly expressed by subpopulations of host-adapted *P. aeruginosa* that may be present in chronic CF infections.

### 4.5. Implications of Carbohydrate-Binding Profile Observations across This P. aeruginosa Collection on Potential Carbohydrate-Based Therapeutics Development and Application to Cystic Fibrosis Patients

*Pseudomonas aeruginosa* has many faces and abilities to adapt and persist in CF despite current antibiotic and airway clearance strategies [1,6,10,20,24,25,26,144]. Through environmental regulation, pathoadaptive mutations, and many other means, this organism is able to tolerate antibiotics and avoid the host immune system [1,6,20,26,44,144]. As such, a one-strategy-fits-all novel add-on therapy for respiratory infections in CF seems unlikely. To prevent chronic colonization, the initial stages of bacterial aggregation, attachment to the host airway, and formation of the biofilm all represent logical first targets for adjunctive therapies to address and complement currently available anti-microbial agents.

To inform this need for aids to traditional anti-infectives, this study surveyed the monosaccharide-binding profiles of whole bacterial cells cultured comparably and simply, using clinical and non-clinical *P. aeruginosa* isolates. The emphasis was on discerning which small molecules bound in vitro, and, thus, could potentially interfere with host–pathogen and pathogen–matrix interactions in vivo, and, thereby, serve as candidates for adjuvants in inhalation therapies. α-Gal, β-GalNAc, and β-Gal3S glycopolymers bound very well across all the strains of *P. aeruginosa* studied. The prevalent binding of the organism to the small molecule α-anomer of galactose and the β-anomer of *N*-acetylgalactosamine suggested that molecules displaying these configurations of these monosaccharides could be candidates to compete at the host glycan–pathogen interface. Previously, clinical strains of *P. aeruginosa* cultured in nutrient-limited conditions supplied with α-Gal, β-GalNAc, or β-Gal3S as the sole carbon sources persisted without significant growth [125], suggesting these may be non-nutritive carbohydrates in vivo. While *P. aeruginosa* characteristics are expected to vary within the CF population and within the individual over time and clinical condition, and what appears to be whole-cell binding was observed in this study for only subpopulations of the cultures, each of the CF and non-CF *P. aeruginosa* did display the same carbohydrate-binding profile. The finding of a significant association of this organism with pendant α-Gal, β-GalNAc, and β-Gal3S monosaccharides suggests that some degree of uniformity in the design of small anti-adhesive therapeutics might be possible.

Small-molecule therapeutics with the α-anomer of galactose, for example, and/or the β-anomer of N-acetylgalactosamine may be good candidates to both compete at the host–pathogen interface and to act to destabilize a *Pseudomonas* biofilm. In our hands, the α-Gal and β-GalNAc glycopolymers show positive cell surface binding to the variety of CF and non-CF *P. aeruginosa* isolates in the collection. This suggests that therapeutic preparations incorporating both monosaccharides may provide biofilm-inhibitory and potentially dissociative activities for the wide variety of *P. aeruginosa* phenotypes expected in the CF airway. In the current study, the lack of visualization of *P. aeruginosa* cell-surface fucose glycopolymer binding for any isolates indicates that additional research is needed to discern whether, in addition to the biofilm-disruptive actions predicted by inhibiting LecB interactions, small molecules presenting fucose may also serve to remove the lectin from the intact bacterial cell membrane.

In search of strategies that enhance the efficacy of antibiotics in the CF airway milieu, it is important to remember that new therapeutics targeting the dispersal of pre-existing biofilms will be releasing biofilm-grown, host-adapted *Pseudomonas* into the airway environment, to scout for or randomly seed the next biofilm [8,27,35,40,42,53]. An advantage to small-molecule carbohydrate therapeutics that target both the bacterial outer membrane and the extracellular elements of the biofilm matrix may be the ability to concomitantly block the new cellular attachments (host and bacterial) while disrupting the established interactions. If such small molecules were monovalent, the risk of the formation of bacterial aggregates or inter-biofilm crosslinks would be less likely than is anticipated with multivalent glycomimics [61,110,112]. Such an issue is important in CF especially as, in so many ways, *P. aeruginosa* aggregation serves to limit the effectiveness of antibiotic and immune cell activities directed at these bacterial cells. Furthermore, if such small disrupting carbohydrates were simultaneously administered with traditional antibiotics, the prediction would be increased drug distribution and effectiveness as the biofilm is dispersed [37,46,52,100,101].

Finally, the precision delivery of antibiotics to the *Pseudomonas* cells or biofilm matrices via lectin targeting is emerging as new antimicrobial strategy [22,25,54,59,62,63,113]. Progress toward the potential for prodrugs delivery to and conversion at the sites of infection was seen with fluoroquinolones in vitro via carbohydrates targeting LecA and LecB [22,62,63]. An early example is that of a mannose-conjugated polymer containing a hydrolysable prodrug of ciprofloxacin which localized with CdrA and LecB at the periphery of a PAO1 biofilm [22]. If inhaled antimicrobial agents could, likewise, specifically target bacterial biofilms, the effective doses could be higher, the host cytotoxicity reduced, and the risks lessened of sub-inhibitory antibiotic concentrations inducing bacterial adaptations that render the drugs ineffective. The use of such therapeutics would further contribute to overall antibiotic conservation. The *P. aeruginosa* carbohydrate-binding profiles reported here suggest that therapeutic agents with α-gal and β-GalNAc entities have the potential to target a wide spectrum of *P. aeruginosa* phenotypes and variants. Even when only a minor subpopulation is specifically bound, localizing prodrug antibiotics, via numerous receptors in type and number, to the portion of the bacterial populations that possess ligands for these monosaccharides may deliver effective antibiotic doses to the entire biofilm communities.

Drawing on experiences of our own and those of many others regarding cystic fibrosis and *P. aeruginosa*, combined with the insights gained in this whole-cell *P. aeruginosa* monosaccharide-binding profiles survey, we offer the following perspectives. Important to the design of an appropriate carbohydrate-based anti-infective strategy for CF infections is the awareness that there are limited carbohydrates and configurations to which whole live *P. aeruginosa* cells appear to bind and limited numbers of bacteria within binding populations that present the adhesive characteristics. Furthermore, and no less important, the challenged and healing host is dynamic in its extracellular macromolecular carbohydrate structure presentation. An aerosolized carbohydrate-based small-molecule anti-adhesive intervention and/or a directed antimicrobial that address(es) multiple targets will likely be the most advantageous for the treatment of this highly variable opportunist *P. aeruginosa* in the widely heterogeneous CF population.

## 5. Conclusions

To inform the development of novel anti-infective agents for cystic fibrosis airway infection, this study addressed the questions of what monosaccharides *P. aeruginosa* from CF patients might be expected to bind, and whether this will vary with the specimen characteristics. Though *P. aeruginosa* adapts in many ways to its host, the carbohydrate-binding profiles obtained across the variety of clinical isolates of this study suggest that a broad spectrum of *P. aeruginosa* will associate with α-galactose and β-N-acetylgalactosamine, and, to a lesser extent, β-galactosamine-3-sulfate. Small-molecule therapeutics presenting these specific anomeric configurations may be anticipated to associate with a small subset of each population of whole bacterial cells present, and this binding would not be expected to be dependent on the *Pseudomonas* source, motility, or mucoidy, or the presence of pili or flagella. The common pendant monosaccharide-PAA-Fluor glycopolymer-binding profile observed across this collection of *P. aeruginosa* suggests that the incorporation of α-galactose and/or β-N-acetylgalactosamine binding elements to add-on inhaled therapeutic interventions intended to target whole bacterial cells may benefit the treatment of the variable natures of CF and non-CF *P. aeruginosa* respiratory infections.

## Figures and Tables

**Table 1:** Glycopolymers and their pendant monosaccharides used in this study ^1,2^.

General Scheme ^1^	Glycopolymer Abbreviation	Pendant Monosaccharide
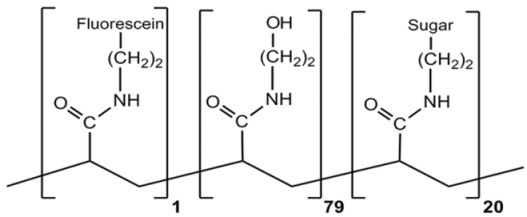	α-Gal-PAA-Fluor	α-D-Galactose
	β-Gal-PAA-Fluor	β-D-Galactose
	α-GalNAc-PAA-Fluor	α-D-*N*-Acetylgalactosamine
	β-GalNAc-PAA-Fluor	β-D-*N*-Acetylgalactosamine
	β-GlcNAc-PAA-Fluor	β-D-*N*-Acetylglucosamine
	β-Gal3S-PAA-Fluor	β-D-Galactose-3-sulfate
	β-GalNAc3S-PAA-Fluor	β-D-*N*-Acetylgalactosamine-3-sulfate
	α-Neu5Ac-PAA-Fluor	α-D-*N*-Acetylneuraminic acid
	α-L-Fuc-PAA-Fluor	α-L-Fucose

^1^ Commercially available multivalent fluoresceinated polyacrylamide (PAA)-based glycopolymers used in bacterial binding assays follow this general structure scheme of multiple residues of specific carbohydrate monomers, spacer molecules, and fluorescein label. Aminoglucitol-PAA-Fluor was included as the negative control. ^2^ Both purchased and synthesized-in-house α-L-Fuc-PAA-Fluor glycopolymer were employed. Details of the synthesis of the precursor compounds, the controlled polymerization reaction of the multivalent glycopolymer with pendant fucose, and the post-polymerization fluoresceination are presented in Supplemental File S1.

**Table 2:** *P. aeruginosa* clinical isolates and laboratory strains evaluated in this study and their agar plate phenotypes.

Culture ID	Plot Order	Isolate or Strain Source ^1^ (Sputum, Throat, Other)	Characteristics ^2,3^
CF-S 8314-1	1	CF sputum, pair #1	Nonmotile, nonmucoid, blue-green diffusible pigment
CF-S 8314-2	2	CF sputum, pair #1	Mucoid
CF-S 8981-1	3	CF sputum, pair #2	Nonmotile, nonmucoid; M9 media restricted growth, TSB cultured
CF-S 8981-2	4	CF sputum, pair #2	Mucoid, brown diffusible pigment on TSA
CF-S 3318	5	CF sputum	Motile; M9 media restricted growth, TSB cultured
CF-S 3247	6	CF sputum	Motile
CF-S 3396	7	CF sputum	Mucoid
CF-S 3443	8	CF sputum	Small colony variant (SCV), nonmotile, nonmucoid
CF-T 3371	9	CF throat, pair #3	Nonmotile, nonmucoid; aggregated in M9 media, TSB cultured
CF-T 3372	10	CF throat, pair #3	Mucoid
CF-T 3435	11	CF throat	Motile
CF-T 3437	12	CF throat	Motile; M9 media restricted growth, TSB cultured
CF-T 3446	13	CF throat	Motile
NCF-H 3380	14	non-CF, hip infection	Motile
NCF-S 3391	15	non-CF, sputum	Motile
ATCC BAA-47	16	other—wound	Motile, green diffusible pigment
ATCC 15442	17	animal room water bottle	Motile
ATCC 33468	18	sputum; not CF tested	Mucoid
Examples of *P. aeruginosa* phenotypes on MacConkey Agar (a) and *Pseudomonas* P Agar (b) ^4^. 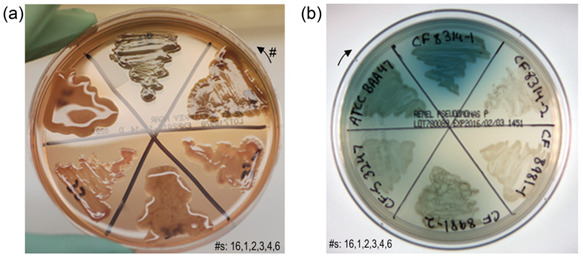			

^1^ Additional characteristics are presented in Supplemental File S2, Figures S2.1 and S2.2, and Tables S2.1 and S2.2, and through source references [123,124,125,126]. CF sputum isolate pairs #1 and #2 are reported on in Chance DL, Mawhinney TP, 2000, *Microbiol.* 146:1717–25 [125]. *P. aeruginosa* strains obtained from the American Type Culture Collection references: ATCC^®^ BAA47™, designated HER-1018[PAO1], deposited by HW Ackemann, originated from BW Holloway, see 1955, *J Gen Microbiol.* 13:572–581 [123] and Klockgether J, 2010, *J Bacteriol.* 192:1113–1121 [126]; ATCC^®^ 15442™, designated PRD-10, deposited by A Beloian; and ATCC^®^ 33468™, human sputum, elder individual not CF tested, Winston-Salem, NC, see Hampton KD, 1979, *J Clin Microbiol.* 9:632–634 [124]. ^2^ Bacterial colony phenotypes of motile, nonmotile, small colony variant (SCV), or mucoid and presence of diffusible pigment reflect growth characteristics on trypticase soy agar (TSA) and MacConkey agar. ^3^ Characteristics of abnormal growth in liquid culture M9 media are noted. For carbohydrate-binding assays, clinical isolates and laboratory strains of *P. aeruginosa* were routinely liquid-cultured in M9 minimal media with glucose [127]; where indicated isolates were cultured in trypticase soy broth (TSB). ^4^ Depicted here and also in Supplemental File S2.2 are strains 1, 2, 3, 4, 6, and 16.

**Table 3:** Comparisons of sources and features of heterogeneous collection of *Pseudomonas aeruginosa* for contribution to or correlation with “higher binding” trends toward specific multivalent fluorescent glycopolymers with pendant monosaccharides α-Gal, β-GalNAc, β-Gal3S, and α-Neu5Ac ^1,2,3,4^.

Source and Culture ID	(#)	Colony Phenotype	Structural Features Confirmed	Preferred Glycopolymer Binding Observed Microscopically Fluorescent Avg # Cells/Field > 1000 Cells	Preferred Glycopolymer Binding Measured Spectroflurometrically Fluorescence Intensity > 6000 AU
CF sputum CF-S 8314-1	1	nonmucoid	flagella, pili	α-Gal, β-GalNAc, β-Gal3S, α-Neu5Ac	α-Gal, β-GalNAc, β-Gal3S, α-Neu5Ac
CF sputum CF-S 8314-2	2	mucoid	flagella	α-Gal	α-Gal, β-GalNAc, β-Gal3S, α-Neu5Ac
CF sputum CF-S 8981-1	3	nonmucoid	flagella	β-GalNAc	α-Gal, β-GalNAc, β-Gal3S
CF sputum CF-S 8981-2	4	mucoid	flagella, pili	**-**	α-Gal, β-GalNAc, β-Gal3S, α-Neu5Ac
CF sputum CF-S 3318	5	motile	flagella, pili	α-Gal	α-Gal, β-GalNAc, β-Gal3S
CF sputum CF-S 3247	6	motile	flagella, pili	**-**	α-Gal, β-GalNAc, β-Gal3S
CF sputum CF-S 3396	7	mucoid	flagella, pili	β-GalNAc, β-Gal3S	α-Gal, β-GalNAc, β-Gal3S
CF sputum CF-S 3443	8	nonmucoid, (SCV)	flagella, pili	α-Gal, β-GalNAc, β-Gal3S	α-Gal, β-GalNAc, β-Gal3S, α-Neu5Ac
CF throat CF-T 3371	9	nonmucoid	flagella	β-GalNAc	α-Gal, β-GalNAc, β-Gal3S
CF throat CF-T 3372	10	mucoid	flagella, pili	α-Gal, β-GalNAc	α-Gal, β-GalNAc, β-Gal3S, α-Neu5Ac
CF throat CF-T 3435	11	motile	flagella, pili	β-GalNAc	α-Gal, β-GalNAc, β-Gal3S
CF throat CF-T 3437	12	motile	flagella, pili	α-Gal, β-GalNAc, β-Gal3S, α-Neu5Ac	α-Gal, β-GalNAc, β-Gal3S, α-Neu5Ac
CF throat CF-T 3446	13	motile	flagella, pili	β-GalNAc	α-Gal, β-GalNAc, β-Gal3S
Non-CF sputum NCF-S 3391	15	motile	pili	α-Gal, β-Gal3S	α-Gal, β-GalNAc, β-Gal3S, α-Neu5Ac
Non-CF sputum ATCC 33468	18	mucoid	pili	α-Gal, β-GalNAc	α-Gal, β-GalNAc, β-Gal3S
Other NCF-H 3380	14	motile	flagella, pili	β-Gal3S	α-Gal, β-GalNAc, β-Gal3S
Other ATCC BAA-47	16	motile	flagella	-	α-Gal, β-GalNAc, β-Gal3S
Other ATCC 15442	17	motile	flagella, pili	-	α-Gal, β-GalNAc, β-Gal3S

^1^ Additional source and phenotypic data are available in Supplemental File S2. ^2^ Multivalent fluorescent glycopolymers with multiple residues of one of nine specific monosaccharides, used to evaluate *Pseudomonas aeruginosa* carbohydrate-binding profiles, include those with pendant α-Gal, β-Gal, α-GalNAc, β-GalNAc, β-GlcNAc, β-Gal3S, β-GalNAc3S, α-Neu5Ac, α-Fuc, and aminoglucitol as the negative control. ^3^ *Pseudomonas* isolates or strains were identified as glycoconjugate “higher binding” in fluorescence microscopy binding assay as plotted in Figure 2b, where an average of greater than 1000 cells per view were enumerated by automated software detection for a specific fluorescent glycopolymer; and as “higher binding” in spectrofluormetric assay, as in Figure 2a, where fluorescence intentisity is >6000 AU. ^4^ Glycopolymer-binding classification was minimal or non-detectable across the strains for PAA-Fluors β-Gal, α-GalNAc, β-GlcNAc, β-GalNAc3S, and α-Fuc.

## Data Availability

Data generated or analyzed during this study are included in this published article and its Supplemental Files.

## Supplementary Materials

The following are available online at https://www.mdpi.com/article/10.3390/microorganisms12040801/s1. Supplemental Material File S1: Fluorescent Glycopolymer Synthesis, Figure S1. Assigned 1H- and 13C-NMR spectra of α-L-Fuc-PAA, and Materials and Methods S1. File S2: *P. aeruginosa* Collection Phenotypic Heterogeneity, Table S2.1. Physiological diversity within the collection of *P. aeruginosa* isolates/Figure S2.1. Phenotypic heterogeneity of selected *P. aeruginosa* specimens on various types of culture media/Figure S2.2. Transmission electron micrographs of negatively stained wet mount specimens, illustrating variable structural features of four *P. aeruginosa* specimens of different colony phenotypes/Table S2.2. Phenotypic heterogeneity in characteristics observed for *P. aeruginosa* isolates and strains/Figure S2.3. Drawings of nine monosaccharides that the bacterial-multivalent fluorescent glycopolymer-binding assays address/Figure S2.4. Example of heterogeneity of binding among *Pseudomonas aeruginosa* strains with regard to same glycopolymer, α-gal-PAA-Fluor, as evaluated by fluorescence microscopy, and Materials and Methods S2. File S3: Additional Binding Results, Figure S3.1. Fluorescence micrograph of minimally washed bacterial-fluorescent glycopolymer suspension in binding assay/Figure S3.2. Transmission electron micrographs of gold-conjugates of fluorescein-immunolabeled β-Gal-PAA-Fluor minimally bound to *P. aeruginosa* clinical isolate CF-S 8314-1 and laboratory strain ATCC BAA47. File S4: Lectin Detection, Figure S4. Electrospray ionization (ESI) positive-ion mode mass spectra of LecA and LecB isolated from *P. aeruginosa* CF-S 8314-1/Table S4. Mass spectral data, theoretical and observed, for lectins isolated from *P. aeruginosa* clinical isolate CF-S 8314-1 and authentic standards of *Pseudomonas* lectins LecA (PA-IL) and LecB (PA-IIL)/Materials and Methods S4. File S5: Probing *P. aeruginosa* Characteristics for Correlations with Enhanced Binding, Table S5.1. Features of *P. aeruginosa* in collection sorted by source/Table S5.2. *P. aeruginosa* “high binder” glycopolymers with features of high-binding strains,/Table S5.3. *P. aeruginosa* high-binding glycopolymers data from Table 3 sorted by phenotype. File S6: Additional Details and Discussions on Characteristics of the CF Airway and *P. aeruginosa* which may Affect *P. aeruginosa* Survival and Success of Adjunctive Anti-Adhesive Therapeutics.
