# Supplementary material for: Insights on Pseudomonas aeruginosa Carbohydrate Binding from Profiles of Cystic Fibrosis Isolates Using Multivalent Fluorescent Glycopolymers Bearing Pendant Monosaccharides
Source: Microorganisms. 2024 Apr 16;12(4):801. doi: 10.3390/microorganisms12040801 (PMC11051836; doi:10.3390/microorganisms12040801)
Supplement: Supplementary file 1 [file microorganisms-12-00801-s001.zip › Supplemental File S1.pdf]

Insights on *Pseudomonas aeruginosa* Carbohydrate Binding from Profiles of Cystic Fibrosis Isolates using Multivalent Fluorescent Glycopolymers Bearing Pendant Monosaccharides

Deborah L. Chance, Wei Wang, James K. Waters, Thomas P. Mawhinney
University of Missouri, Columbia, MO, 65211, USA

Supplementary Material

Supplemental File S1: Fluorescent Glycopolymer Synthesis

Figure S1. Assigned ^{1}H- (a) and ^{13}C-NMR (b) spectra (D_2O) of α-L-Fuc-PAA.
Materials and Methods S1

Supplemental File S2: *P. aeruginosa* Collection Phenotypic Heterogeneity

Table S2.1. Physiological diversity within the collection of *P. aeruginosa* isolates and strains
Figure S2.1. Phenotypic heterogeneity of selected *P. aeruginosa* specimens on various types of culture media.
Figure S2.2. Transmission electron micrographs of negatively stained wet mount specimens, illustrating variable structural features of four *P. aeruginosa* specimens of different colony phenotypes.
Table S2.2. Phenotypic heterogeneity in characteristics observed for *P. aeruginosa* isolates and strains
Figure S2.3. Drawings of nine monosaccharides which the bacterial-multivalent fluorescent glycopolymer binding assays address.
Figure S2.4. Example of heterogeneity of binding among *Pseudomonas aeruginosa* strains with regard to same glycopolymer, α-gal-PAA-Fluor, as evaluated by fluorescence microscopy.
Materials and Methods S2

Supplemental File S3: Additional Binding Results

Figure S3.1. Fluorescence micrograph of minimally washed bacterial-fluorescent glycopolymer suspension in binding assay.
Figure S3.2. Transmission electron micrographs of gold-conjugates of fluorescein-immunolabeled β-Gal-PAA-Fluor minimally bound to *P. aeruginosa* clinical isolate CF-S 8314-1 (a) and laboratory strain ATCC BAA47 (b).

Supplemental File S4: Lectin Detection

Figure S4. Electrospray ionization (ESI) positive ion mode mass spectra of LecA (a) and LecB (b) isolated from *P. aeruginosa* CF-S 8314-1.
Table S4. Mass spectral data, theoretical and observed, for lectins isolated from *P. aeruginosa* clinical isolate CF-S 8314-1 and authentic standards of *Pseudomonas* lectins LecA (PA-IL) and LecB (PA-IIL)
Materials and Methods S4

Supplemental File S5: Probing *P. aeruginosa* Characteristics for Correlations with Enhanced Binding

Table S5.1. Features of *P. aeruginosa* in collection sorted by source
Table S5.2. *P. aeruginosa* "high binder" glycopolymers with features of high binding strains
Table S5.3. *P. aeruginosa* high binding glycopolymers data from Table 3 sorted by phenotype

Supplemental File S6: Additional Details and Discussions on the Characteristics of CF Airway and *P. aeruginosa* which may Affect *P. aeruginosa* Survival and Success of Adjunctive Anti-Adhesive Therapeutics

Supplemental File S1

Fluorescent Glycopolymer Synthesis

Figure S1. Assigned ^{1}H- (a) and ^{13}C-NMR (b) spectra (D_2O) of α-L-Fuc-PAA.

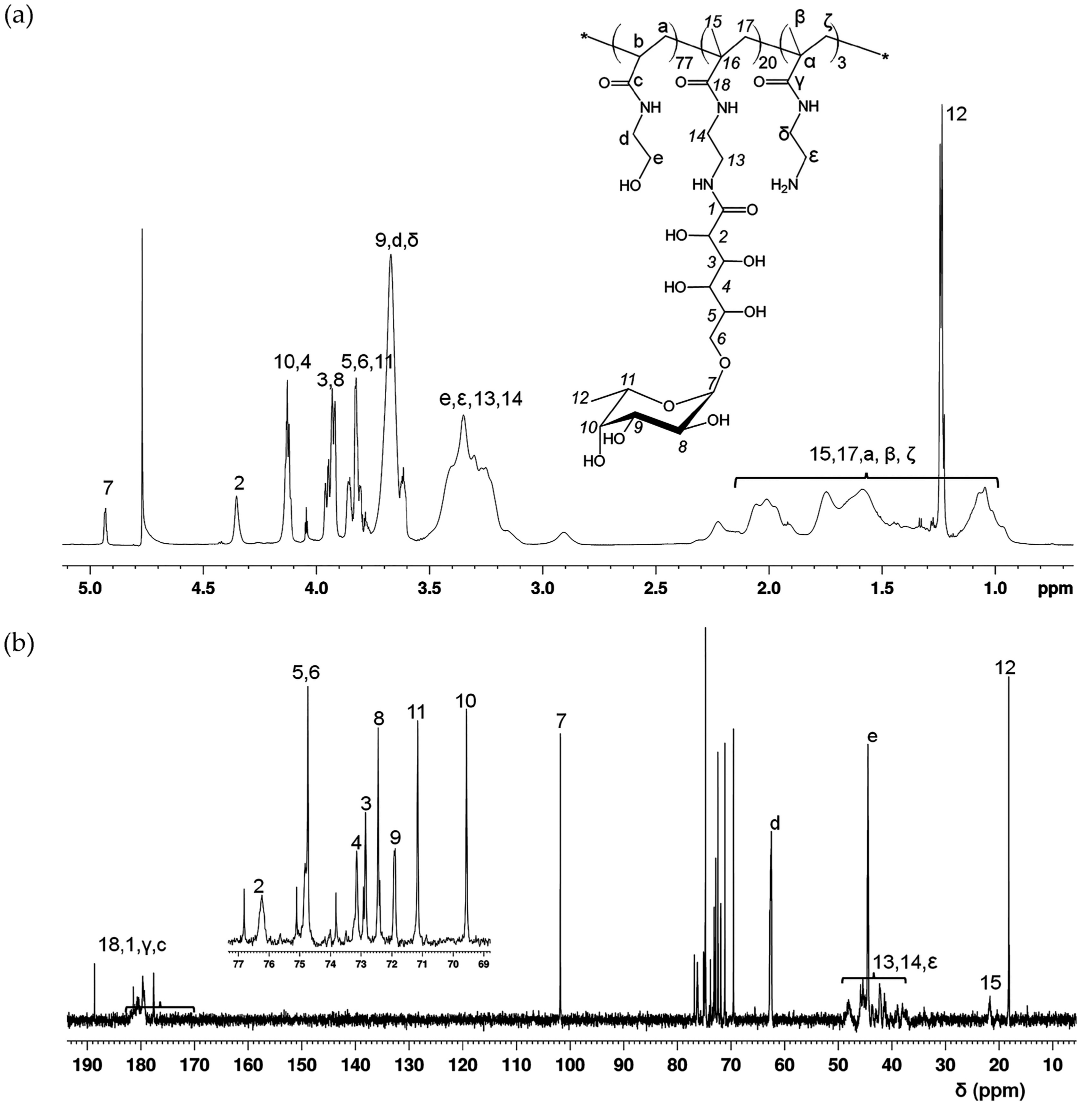

Materials and Methods S1

Preparation of reversible addition–fragmentation chain-transfer (RAFT) polymerization-based fluorescent glycopolymer *α*-L-Fuc-PAA-Fluor

Materials

N-(2-aminoethyl) methacrylamide hydrochloride was obtained from Polysciences (Warrington, PA). The fluorophore 5-(and-6)-carboxyfluorescein succinimidyl ester were purchased from Molecular Probes (Grand Island, NY). *Aleuria aurantia* lectin-coated agarose beads were from Vector Laboratories (Burlingame, CA). Other chemicals, not noted above, were purchased from Sigma-Aldrich Chemicals (St. Louis, MO).

Synthesis of *α*-L-fucopyranosyl-(1→6)-D-glucose

α-L-Fucopyranosyl-(1→6)-D-glucose was prepared according to a published method with some modifications (Uchiyama and Hindsgaul 1996). Briefly, to a stirring solution of L-fucose (2.0 g) and triethylamine (8.8 mL, TEA)) in 60 mL of dry dimethylformamide (DMF), 8 mL of chlorotrimethylsilane (TMS-Cl) were added at 0°C and then the reaction was stirred for 4 h at room temperature. Pentane (100 mL) and ice (40 mL) were then added and mixed well. The organic layer was separated and washed with cold water (40 mL x 3), dried over magnesium sulfate and evaporated to obtain per-*O*-trimethylsilyl-L-fucose (4.4 g, 80% yield).

To a stirring solution of per-*O*-trimethylsilyl-L-fucose (2.7 g) in 20 mL of dry CH_2Cl_2 was added 0.86 mL of iodotrimethylsilane (TMS-I). Following 20 min of stirring at room temperature, the mixture was added to a solution of 2.3 g of 6-hydroxy-tetraacetyl-D-glucose (synthesized as per Reynolds and Evans 1942) and 2,6-di-*tert*-butylpyridine (1.34 mL) in dry methylene chloride (40 mL). After 5 h of stirring at room temperature, 60 mL of MeOH was added to the reaction and stirred for 20 min to remove the trimethylsilyl groups. The mixture was neutralized with ion exchange resin, filtered, and concentrated. The sample was then acetylated by dissolving in 5 mL of pyridine, adding 10 mL of acetic anhydride and stirring at 50 °C for 2 hr. After blowing down the reaction with air, chloroform (80 mL) and water (40 mL) were then added to the sample and mixed well. The organic layer was separated and washed with water (40 mL x 3), dried over magnesium sulfate and evaporated. The product *α*-L-fucopyranosyl-(1→6)-D-glucose heptaacetate was purified with silica gel column chromatography (hexane: ethyl acetate, 3:2), (2.02 g, 54% yield).
ESI-MS (in chloroform: methanol, v: v 1:1): calculated *m/z* for $C_{26}H_{36}O_{17}$+Na^+, 643.18447; observed m/z 643.18684.
^{1}H NMR (600 MHz, $CDCl_3$): δ (ppm) 5.68 (d, $J_{1,2}$=7.8, 1H, H-1), 5.35 (dd, 1H, H-4′), 5.32 (dd, 1H, H-2′), 5.26-5.21 (dd, 1H, H-2), 5.14-5.11 (m, 3H, H-3, H-3′, H-5), 5.00 (d, $J_{1',2'}$=3.6, 1H, H-1′), 4.18-4.15 (m, 1H, H-5′), 3.82-3.78 (m, 1H, H-4), 3.77-3.73 (dd, H, H-6a), 3.55-3.51 (dd, 1H, H-6b), 2.16 (s, 3H, COCH_3), 2.14 (s, 3H, COCH_3), 2.12 (s, 3H, COCH_3), 2.05 (s, 3H, COCH_3), 2.04 (s, 3H, COCH_3), 2.02 (s, 3H, COCH_3), 2.00 (s, 3H, COCH_3), 1.13 (d, $J_{5',6'}$=6.6, 3H, H-6′); ^{13}C NMR (150 MHz, $CDCl_3$): δ (ppm) 170.82, 170.75, 170.16, 170.05, 169.24, 169.20, 169.10 (7 $COCH_3$), 97.21 (C-1′), 91.63 (C-1), 73.73(C-4), 73.12 (C-2), 71.04 (C-2′), 70.17 (C-5), 68.36 (C-3′), 68.02 (C-4′), 67.74 (C-3), 66.17 (C-6), 64.57 (C-5′), 20.89, 20.79, 20.76, 20.70, 20.67, 20.64, 20.57 (7 $COCH_3$), 15.85 (C-6′).

To deacetylate the compound, 1.0 g of the disaccharide heptaacetate was dissolved in 2 mL of CH_2Cl_2, to which was added 50 mL of MeOH and 1.0 mL of sodium methoxide solution (25 wt. % in dry methanol). The mixture was stirred at room temperature for 20 min, neutralized with cation exchange resin (Dowex

50Wx8, H^+ form), filtered to remove the resin, and evaporated *in vacuo* to give the disaccharide α-L-fucopyranosyl-(1→6)-D-glucose with a quantitative yield.
ESI-MS (in methanol: water, v: v 1:1): calculated *m/z* for $C_{12}H_{22}O_{10}$+Na^+, 349.11052; observed m/z 349.11032.
^{1}H NMR (800 MHz, D_2O): δ (ppm) 5.23 (d, $J_{1,2}$=4, 1H, H-1 α-Glc), 4.93 (d, $J_{1',2'}$=4, 1H, H-1'), 4.65 (d, $J_{1,2}$=8, 1H, H-1 β-Glc), 4.13-3.99 (m, 1H, H-5'), 3.90 (dd, 1H, H-2'), 3.61 (m, 1H, H-4 β-Glc), 3.55 (dd, 1H, H-2 α-Glc), 3.49 (dd, 1H, H-3 β-Glc), 3.25 (dd, 1H, H-2 β-Glc), 1.21 (d, 3H, H-6'); ^{13}C NMR (200 MHz, D_2O): δ (ppm) 102.32, 101.98 (C-1'), 98.87 (C-1 β-Glc), 94.95 (C-1 α -Glc), 78.50, 77.85, 76.96, 75.52, 74.69, 74.28, 73.60, 72.67, 72.51, 72.36, 71.06, 70.67, 70.23, 69.60 (C-5'), 17.90 (C-6').

Synthesis of fucose-glucono-1,5-lactone

Synthesis of fucose-glucono-1,5-lactone proceeded similarly to the described synthesis of other disaccharide lactones (Wang *et al.* 2014). To a solution of α-L-fucopyranosyl-(1→6)-D-glucose (0.4 g) dissolved in 1.0 mL of water and 2.0 mL of MeOH, was added an iodine solution in MeOH (0.6 g in 8 mL) at 40°C. A 4% solution of potassium hydroxide in MeOH (15 mL, w/v) was then added dropwise over ~15 min, and the mixture was stirred at 40°C until the iodine color completely disappeared. The mixture was then cooled on ice. The precipitate was filtered and rinsed with small amount of cold MeOH, then dissolved in 0.5 mL of water, and re-precipitated in 25 mL of -20°C methanol. The product in potassium salt form was converted to the free acid via passage as an aqueous solution through a small cation-exchange resin column, and was then freeze dried. Following disolution of the free acid in a small amount of MeOH, absolute EtOH was added just until the solution turned cloudy; then the solvents were removed by rotoevaporation. The lactone was formed by repeating this cycle (5 times), concentrating and drying the acid with evaporation *in vacuo* from the methanol and absolute ethanol solution (330 mg, 85% yield).

Synthesis of glycomonomer 6-*O*-α-L-fucopyranosyl-D-gluconamidoethyl methacrylamide

To a solution of the disaccharide lactone (330 mg) in 2.0 mL of methanol were added 2.0 mL of methanol containing *N*-(2-aminoethyl) methacrylamide hydrochloride (AEMA hydrochloride,197 mg), hydroquinone monomethyl ether (MEHQ, an inhibitor of self-polymerization, 0.5 mg), and 0.3 mL triethylamine. Following 16 h of stirring at room temperature, 10 mL of water were added, and the methanol and triethylamine in the mixture were removed under vacuum. The aqueous solution was then passed through an anion exchange column into a receiving beaker containing 0.5 mg of MEHQ. After the newly released triethylamine was removed *in vacuo*, the solution was passed through a cation exchange resin column and freeze-dried. To remove MEHQ, the product was dissolved in a minimum amount of MeOH, and precipitated in cold CH_2Cl_2 to give the glycomonomer: 6-*O*-α-L-fucopyranosyl-D-gluconamidoethyl methacrylamide (424 mg, 92% yield).
ESI-MS (in methanol: water, v:v 1:1): calculated *m/z* for $C_{18}H_{32}O_{11}N_2$+Na^+, 475.18983; observed m/z 475.19363.
^{1}H NMR (800 MHz, D_2O): δ (ppm) 5.70 (s, 1H, H-17A), 5.46 (s, 1H, H-17B), 4.92 (d, $J_{7,8}$=3.2, 1H, H-7), 4.30 (d, $J_{2,3}$ =3.2, 1H, H-2), 4.11 (m, 1H, H-10), 4.08 (m, 1H, H-4), 3.94 (m, 1H, H-3), 3.90 (dd, 1H, H-8), 3.83 (m, 1H, H-5), 3.82-3.79 (m, 2H, H-6), 3.80-3.75 (m, 2H, H-12), 3.79 (m, 1H, H-11), 3.60 (dd, 1H, H-9), , 3.47-3.40 (m, 4H, H-13, H-14), 1.92 (s, 3H, H-15), 1.22 (d, 3H, H-12). ^{13}C NMR (200 MHz, D_2O): δ (ppm) 177.59 (C-1), 175.02 (C-18), 141.89 (C-16), 124.08 (C-17), 101.82 (C-7), 76.13 (C-2), 74.73 (C-5), 73.24 (C-4), 72.85 (C-3), 73.44 (C-8), 71.87 (C-9), 77.15 (C-11), 69.59 (C-10), 58.86 (C-6), 41.79 (C-13), 41.36 (C-14), 20.52 (C-15), 18.17 (C-12).

Synthesis of RAFT-based polyacrylamide (PAA) glycopolymer α-L-Fuc-PAA

A controlled reversible addition-fragmentation chain-transfer (RAFT)-based copolymerization strategy (Wang *et al.* 2014) was used to synthesize the PAA-based multivalent fucose glycopolymer. To a 1 mL Schlenk tube was added 0.4 mL water containing 31.6 mg of 6-*O*-α-L-fucopyranosyl-D-gluconamidoethyl methacrylamide, 1.7 mg of *N*-(2-aminoethyl) methacrylamide (AEMA hydrochloride) and 27.5 μL of *N*-(2-hydroxyethyl) acrylamide (HEAA). To the mixture were then added sequentially 50 μL of dimethylformamide (DMF) containing 0.53 mg of (4-cyanopentanoic acid)-4-dithiobenzoate (the chain transfer agent, CTA) and another 50 μL of DMF containing 250 μg of 4, 4 -azobis-(4-cyanovaleric acid) (the initiator). The solution was degassed with 3 freeze–evacuate–thaw cycles, and then kept in a water bath at 70°C for 24 h. The solution was then dialyzed against deionized water (6 x 2 L) over a period of 24 h (*MWCO*=3,500) and lyophilized to obtain the α-L-Fuc-PAA (61 mg, 93.8% yield). The glycopolymer dispersity (1.28) and degree of polymerization (95mer) were determined by gel permeation chromatography (GPC) as previously described (Wang *et al.* 2014). ^{1}H and ^{13}C NMR spectra of α-L-Fuc-PAA are shown in Fig. S1.

Post-modification of α-L-Fuc-PAA with fluorophore

This PAA-based glycopolymer was fluoresceinated following protocol previously described for other glycopolymers (Wang *et al.* 2014). Briefly, to a rapidly stirring solution of α-L-Fuc-PAA (5.0 mg dissolved in 0.9 mL of phosphate buffered saline, pH 7.5), was added carboxyfluorescein succinimidyl ester solution (0.6 mg in 100 μL DMF) and the reaction was allowed to proceed, stirring, for 16 h in the dark, at RT. The solution was then dialyzed against distilled water (2L for 6 changes over 24 h, *MWCO*=3,500), then lyophilized, resulting in a flocculent yellow product. The lectin-binding ability of the fluorescent glycopolymer containing α-L-fucoside as the pendant sugar was confirmed by testing with *Aleuria aurantia* lectin-coated agarose beads as described in the main text using a previously published method (Wang *et al.* 2014).

Analysis of the synthetic products

^{1}H and ^{13}C NMR spectra were obtained for samples dissolved in D_2O on a Bruker Avance 800 MHz NMR Spectrometer (Grant supported purchase: NIH/NCRR S10 RR022341-01). ^{1}H and ^{13}C spectra were recorded at 800.14 and 200.19 MHz, respectively. Mass spectra were collected for glycomonomer, and other synthetic products, dissolved in MeOH/water 50:50 (v/v), via direct infusion electrospray ionization (ESI) in the positive ion mode utilizing a Thermo Scientific LTQ Orbitrap XL Mass Spectrometer. Glycopolymer dispersity (*Mw*/*Mn*) by gel permeation chromatography (GPC) (Wang *et al.* 2014) was derived against a calibration curve from polyethylene glycol standards (MW: 200-1,200,000 g/mol) using a Waters Alliance HPLC System equipped with a refractive index detector and TOSOH TSK-GEL G4000 PWxl and TSK-GEL G4000 SW GPC columns, eluting with 0.1M Tris/0.1M sodium chloride buffer (pH=7) at a flow rate of 0.6 mL/min.

Abbreviations

DMF, dimethylformamide; ESI, electrospray ionization; GPC, gel permeation chromatography; *m*/*z*, mass/charge; MEHQ, hydroquinone monomethyl ether; *Mn*, Number-average molar molecular weight; *Mw*, Weight-average molar molecular weight; *MWCO*, molecular weight cut-off; PAA, polyacrylamide; PAA-Fluor, polyacrylamide-based fluorescent polymer; PBS, phosphate buffered saline; RAFT, reversible addition–fragmentation chain-transfer; TEA, triethylamine; AEMA, *N*-(2-aminoethyl) methacrylamide; HEAA, *N*-(2-hydroxyethyl) acrylamide; CTA, chain transfer agent.

References

Reynolds DD, Evans WL. 1942. β-D-Glucose-1,2,3,4-tetraacetate. *Org. Synth*. 22:56-58.

Uchiyama T, Hindsgaul O. 1996. Per-*O*-trimethylsilyl-α-L-fucopyranosyl iodide: a novel glycosylating agent for terminal α-L-fucosylation. *Synlett*. 06:499-501.

Wang W, Chance DL, Mossine VV, Mawhinney TP. 2014. RAFT-based tri-component fluorescent glycopolymers: synthesis, characterization and application in lectin-mediated bacterial binding study. *Glycoconj J*. 31:133-143.
