# Supplementary material for: Insights on Pseudomonas aeruginosa Carbohydrate Binding from Profiles of Cystic Fibrosis Isolates Using Multivalent Fluorescent Glycopolymers Bearing Pendant Monosaccharides
Source: Microorganisms. 2024 Apr 16;12(4):801. doi: 10.3390/microorganisms12040801 (PMC11051836; doi:10.3390/microorganisms12040801)
Supplement: Supplementary file 1 [file microorganisms-12-00801-s001.zip › Supplemental File S2.pdf]

Insights on *Pseudomonas aeruginosa* Carbohydrate Binding from Profiles of Cystic Fibrosis Isolates using Multivalent Fluorescent Glycopolymers Bearing Pendant Monosaccharides

Deborah L. Chance, Wei Wang, James K. Waters, Thomas P. Mawhinney
University of Missouri, Columbia, MO, 65211, USA

Supplemental File S2

***P. aeruginosa* Collection Phenotypic Heterogeneity**

<u>**Contents**</u>

Table S2.1. Physiological diversity within the collection of *P. aeruginosa* isolates and strains

Figure S2.1. Phenotypic heterogeneity of selected *P. aeruginosa* specimens on various types of culture media.

Figure S2.2. Transmission electron micrographs of negatively stained wet mount specimens, illustrating variable structural features of four *P. aeruginosa* specimens of different colony phenotypes.

Table S2.2. Phenotypic heterogeneity in characteristics observed for *P. aeruginosa* isolates and strains

Figure S2.3. Drawings of nine monosaccharides which the bacterial-multivalent fluorescent glycopolymer binding assays address.

Figure S2.4. Example of heterogeneity of binding among *Pseudomonas aeruginosa* strains with regard to same glycopolymer, α-gal-PAA-Fluor, as evaluated by fluorescence microscopy.

Materials and Methods S2

Table S2.1. Physiological diversity within the collection of *P. aeruginosa* isolates and strains [a,b,c]

Activities/Substrate Utilization Tests (Automated System Well ID#)	% Positive (#) (total n=18)	Distribution of NCF & CF % Positive (#) NCF (n=5)	CF nonmucoid (n=9)	CF mucoid (n=4)
Glutamyl arylamidase pNA (GN-12)	39% (7)	40% (2)	44% (4)	25% (1)
D-Mannitol (GN-19)	22% (4)	60% (3)	11% (1)	0% (0)
D-Mannose (GN-20)	89% (16)	100% (5)	89% (8)	75% (3)
Lipase (GN-26)	39% (7)	40% (2)	22% (2)	75% (3)
Tyrosine arylamindase (GN-29)	56% (10)	80% (4)	44% (4)	50% (2)
D-Trehalose (GN-35)	11% (2)	0% (0)	11% (1)	25% (1)
Malonate (GN-37)	72% (13)	100% (5)	89% (8)	0% (0)
L-Histidine assimilation (GN-53)	11% (2)	20% (1)	11% (1)	0% (0)
Coumarate (GN-56)	67% (12)	80% (4)	78% (7)	25% (1)
0/129 Resistance (GN-58)	50% (9)	60% (3)	67% (6)	0% (0)
L-Malate assimilation (GN-61)	72% (13)	100% (5)	89% (8)	0% (0)
L-Lactate assimilation (GN-64)	50% (9)	60% (3)	67% (6)	0% (0)

[a] Clinical isolates and laboratory strains used in this study were obtained through a variety of sources (see Table S2.2). NCF desination refers to non-cystic fibrosis strains obtained from environmental sources or patients not diagnosed with cystic fibrosis; CF designations of nonmucoid or mucoid refer to non-mucoid or mucoid isolates from patients with cystic fibrosis (CF).

[b] Activities, from a panel of 48 tests, for which collection of 18 strains of *Pseudomonas aeruginosa* in this study shows heterogeneity between specimens as evaluated by the automated identification system Vitek2 with Gram negative cards. In this case, activities were characterized as heterogeneous in the collection when >10% of the specimens were either positive for a feature many others were negative for, or negative for an activity many others were positive for.

[c] Physiological & utilization activities for which > 90% of the tested *Pseudomonas aeruginosa* specimens were either positive or negative are listed below.

> 90% Tested Isolates & Strains Positive for (7 activities): D-Glucose (GN-13); gamma-Glutamyl-transferase (GN-14); β-Alanine arylamidase pNA (GN-22); L-Proline arylamidase (GN-23); Citrate (Sodium) (GN-36); L-Lactate alkalinization (GN-40); Succinate alkalinization (GN-42).

>90% Tested Isolates & Strains Negative for (28 activities): Ala-Phe-Pro-arylamidase (GN-2); Adonitol (GN-3);L-Pyrrolydonyl-arylamidase (GN-4); L-Arabitol (GN-5); D-Cellobiose (GN-7); β-Galactosidase (GN-9); H2S Production (GN-10); β-N-acetyl-Glucosaminidase (GN-11); Fermentation/ Glucose (GN-15); β-Glucosidase (GN-17); D-Maltose (GN-18); β-Xylosidase (GN-21); Palatinose (GN-27); Urease (GN-31); D-Sorbitol (GN-32); Saccharose/sucrose (GN-33); D-Tagatose (GN-34); 5-keto-D-Gluconate (GN-39); α-Glucosidase (GN-41); β-N-acetyl-Galactosaminidase (GN-43); α-Galactosidase (GN-44); Phosphatase (GN-45); Glycine arylamidase (GN-46); Ornithine decarboxylase (GN-47); Lysine decarboxylase (GN-48); β-glucuronidase (GN-57); Glu-Gly-Arg-arylamidase (GN-59); Ellman (GN-62).

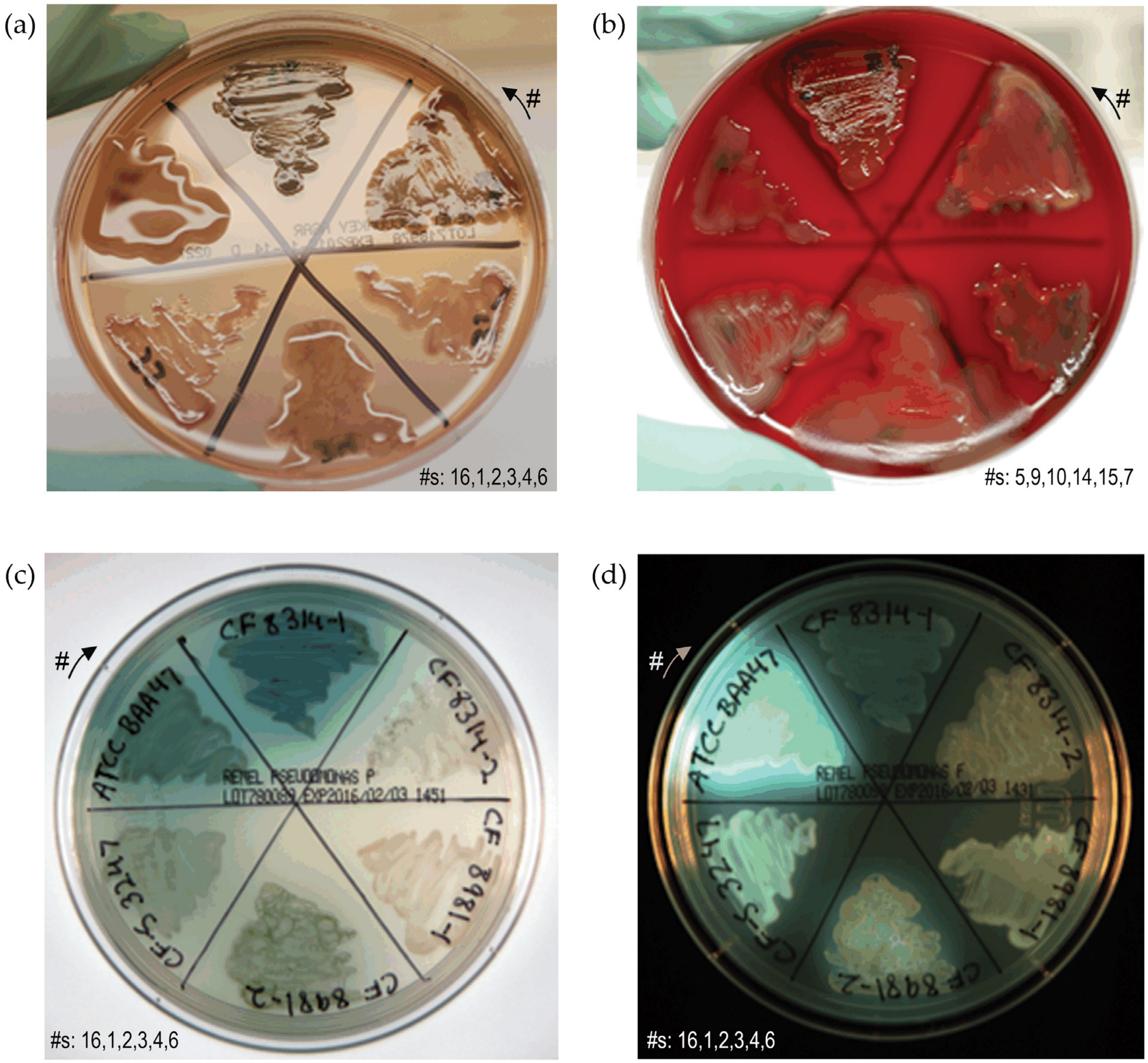

Figure S2.1. Phenotypic heterogeneity of selected *P. aeruginosa* specimens on various types of culture media. (a) MacConkey agar (plot order #s 16,1,2,3,4,6), highlighting motile, nonmotile nonmucoid, and mucoid morphotypes; (b) Blood agar (plot order #s 5,9,10,14,15,7), showing varied degrees of hemolysis and motility; (c) *Pseudomonas* P agar (#s 16,1,2,3,4,6), illustrating variable amount and color of pigments elaborated, specifically green and blue-green pyocyanin; (d) 366nm illumination of *Pseudomonas* F agar (#s 16,1,2,3,4,6), demonstrating varied fluorescent pigment production.

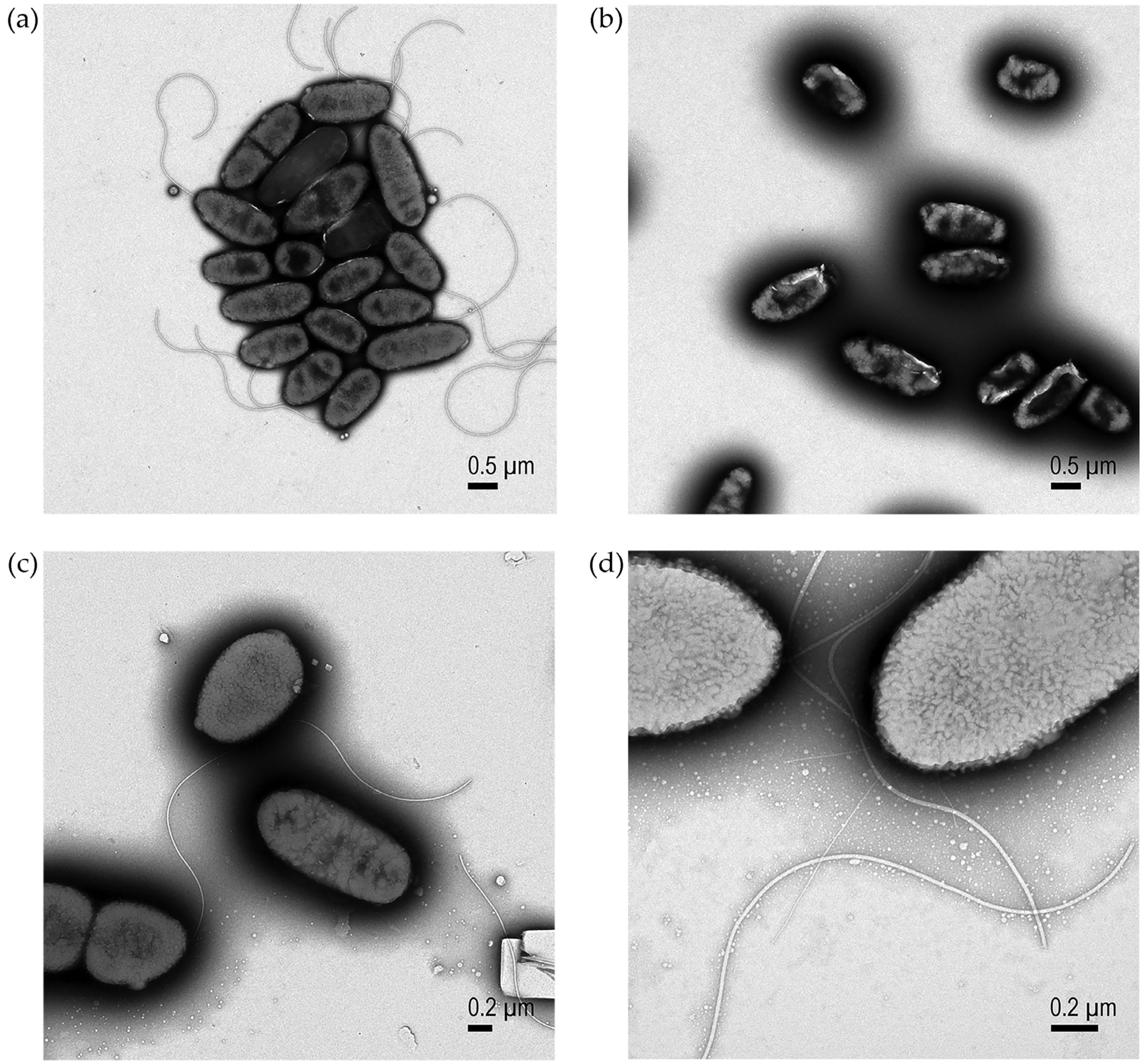

Figure S2.2. Transmission electron micrographs of negatively stained wet mount specimens, illustrating variable structural features of four *P. aeruginosa* specimens of different colony phenotypes. (a) ATCC BAA47 [PAO1] (plot order #16), motile, with flagella; (b) ATCC 33468 (#18), mucoid, non-motile, no flagella; (c) CF-S 3443 (#8), SCV (small colony variant), non-motile with flagella; (d) CF-S 8314-1 (#1), non-mucoid, non-motile, with flagella and pili. Original magnifications: (a) 2.5K; (b) 2.5K; (c) 5K; (d) 10K.

Table S2.2. Phenotypic heterogeneity in characteristics observed for *P. aeruginosa* isolates and strains

				Colony Morphotype			Isolate or Strain Source [a,b]		Pigments [c]		Structural Features [d]		Relative CFU [e]	
Culture ID	Plot order	Media	Lab ID	motile (mot)	motile, non-mucoid	mucoid (muc)	pair	culture source (sputum, throat, other)	pigment on PPA	fluor-escence on PFA	flagella	pili	CFU/ml of 1.0 OD_{600} nm	CFU Index
CF-S 8314-1	1		# 31		nm		CF pair #1	CF sputum	++	(+)	+	+	2.0E+09	0.56
CF-S 8314-2	2		# 32			muc	CF pair #1	CF sputum	-	(+)	+	-	2.8E+09	0.78
CF-S 8981-1	3	TSB	# 33		nm		CF pair #2	CF sputum	-	+	+	-	3.4E+09	0.94
CF-S 8981-2	4		# 34			muc	CF pair #2	CF sputum	+	+	+	+	5.7E+09	1.58
CF-S 3318	5	TSB	# 37	mot				CF sputum	-	++	+	(+)	3.0E+09	0.83
CF-S 3247	6		# 35	mot				CF sputum	+	+	+	(+)	4.9E+09	1.36
CF-S 3396	7		# 42			muc		CF sputum	+	(+)	+	+	4.4E+09	1.22
CF-S 3443	8		# 45		nm (SCV)			CF sputum	-	(+)	+	(+)	2.7E+09	0.75
CF-T 3371	9	TSB	# 38		nm		CF pair #3	CF throat	-	-	+	-	3.6E+09	1.00
CF-T 3372	10		# 39			muc	CF pair #3	CF throat	(+)	(+)	+	(+)	4.9E+09	1.36
CF-T 3435	11		# 43	mot				CF throat	+	+	+	+	3.4E+09	0.94
CF-T 3437	12	TSB	# 44	mot				CF throat	+	+	+	+	1.9E+09	0.53
CF-T 3446	13		# 46	mot				CF throat	+	++	+	+	2.8E+09	0.78
NCF-H 3380	14		# 40	mot				non-CF, hip	-	(+)	+	(+)	4.8E+09	1.33
NCF-S 3391	15		# 41	mot				non-CF, sputum	-	++	-	+	5.5E+09	1.53
ATCC BAA-47	16		# 30	mot				other - wound, Australia, HER-1018[PA01]	++	+++	+	-	5.3E+09	1.47
ATCC 15442	17		# 48	mot				other - animal room water bottle, PRD-10	-	++	+	(+)	5.5E+09	1.53
ATCC 33468	18		# 47			muc		other - sputum; not tested for CF	-	(+)	-	(+)	2.9E+09	0.81
% of strains positive for feature				50%	22%	28%			50%	94%	89%	78%		

[a] *Pseudomonas aeruginosa* clinical isolates with "pair" indication were obtained as two distinct phenotypes from same patient specimen.

[b] *Pseudomonas aeruginosa* strains obtained from the American Type Culture Collection references: ATCC® BAA47™, deposited by HW Ackemann, originated from BW Holloway, see *J. Gen. Microbiol.* 13:572-581, 1955, J Klockgether *J. Bacteriol.* 192:1113-1121, 2010; ATCC® 15442™, deposited by A Beloian; ATCC® 33468™, human sputum, elder individual not tested for CF, Winston-Salem, NC, see KD Hampton *J. Clin. Microbiol.* 9:632-634, 1979.

[c] Pigments and intensities observed on specialized media *Pseudomonas* P Agar (PPA, for enhancement of pyocyanin production) or *Pseudomonas* F Agar (PFA, for enhancement of fluorescein detection) are classified as present in abundance +, weakly (+), or not detectible or discernable -. Positive pigment production may reflect production of additional or alternate pigments or siderophores to those specifically enhanced by these media types. Additionally, the presence of visible pigments may quench and thereby reduce fluorescent compound detection.

[d] Structural features flagella and pili were observed by transmission electron microscopy (TEM) following staining with nano-W (Nano-W® , methylamine tungstate, negative stain; Nanoprobes, Inc., Yaphank, NY), as recently described (Chance and Mawhinney 2017). Evaluation of each isolate or strain feature was scored as + (positive) when readily observed for each specimen examined, (+) when infrequently observed, and - (negative) as not detected. The presence of functional pili was evaluated through a twitching assay (as stabs into fresh Luria-Bertani media with 1.5% agar in petri plates), and observation of irregular leading edge growth patterns at the agar-plastic interface confirmed the presence of functional pili for each positive + or weakly positive (+) strain evaluated. Pili negative strains by TEM likewise did not clearly demonstrate irregular growth edges in functional pili/twitching assay.

[e] While the average CFU per 1ml of 1.0 OD600 nm suspension for this collection was 3.9E+09, the actual CFU per ml at this OD varies between strains reflecting heterogeneity of optical density characteristics of the different isolates or strains. Mucoid strains, for example are often more transparent, requiring more organisms to reach the experimental target optical density. CFU index is employed to normalize binding data to comparable input number of organisms.

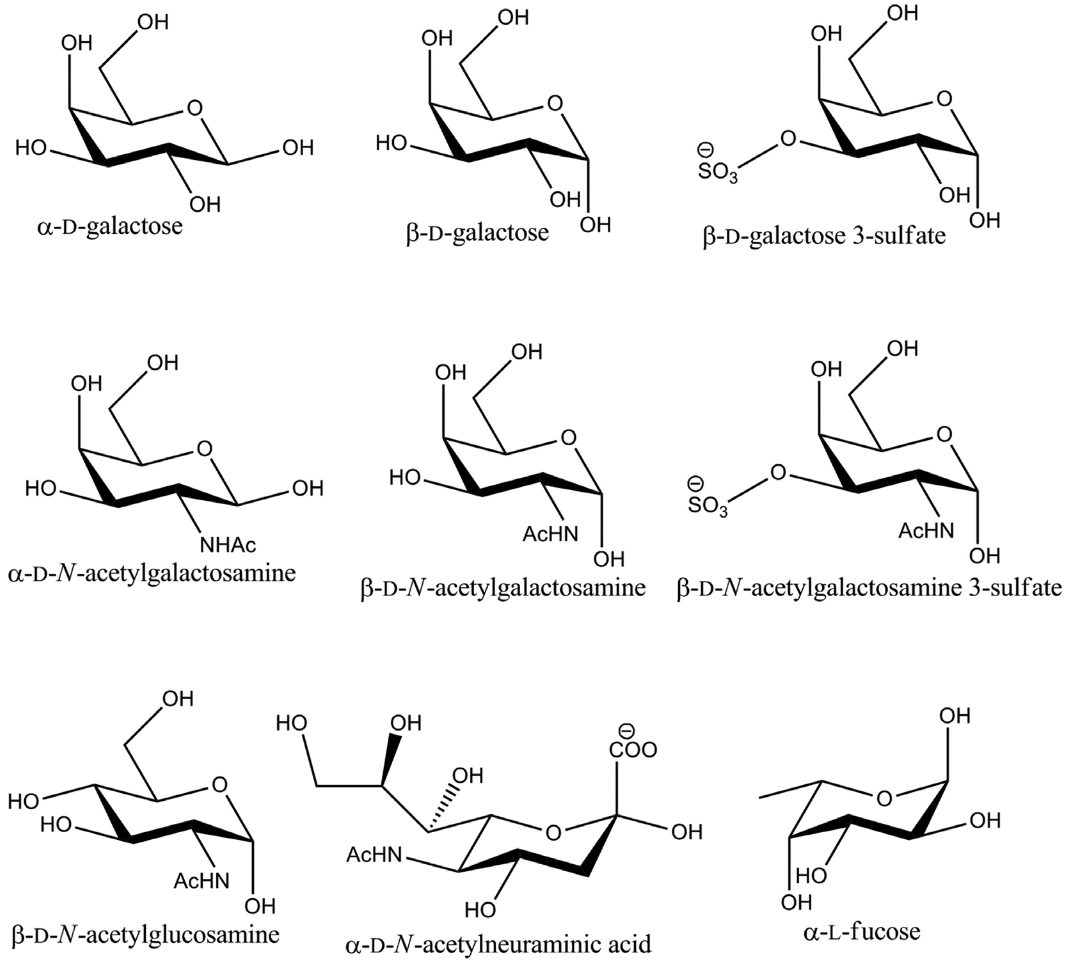

Figure S2.3. Drawings of nine monosaccharides which the bacterial-multivalent fluorescent glycopolymer binding assays address.

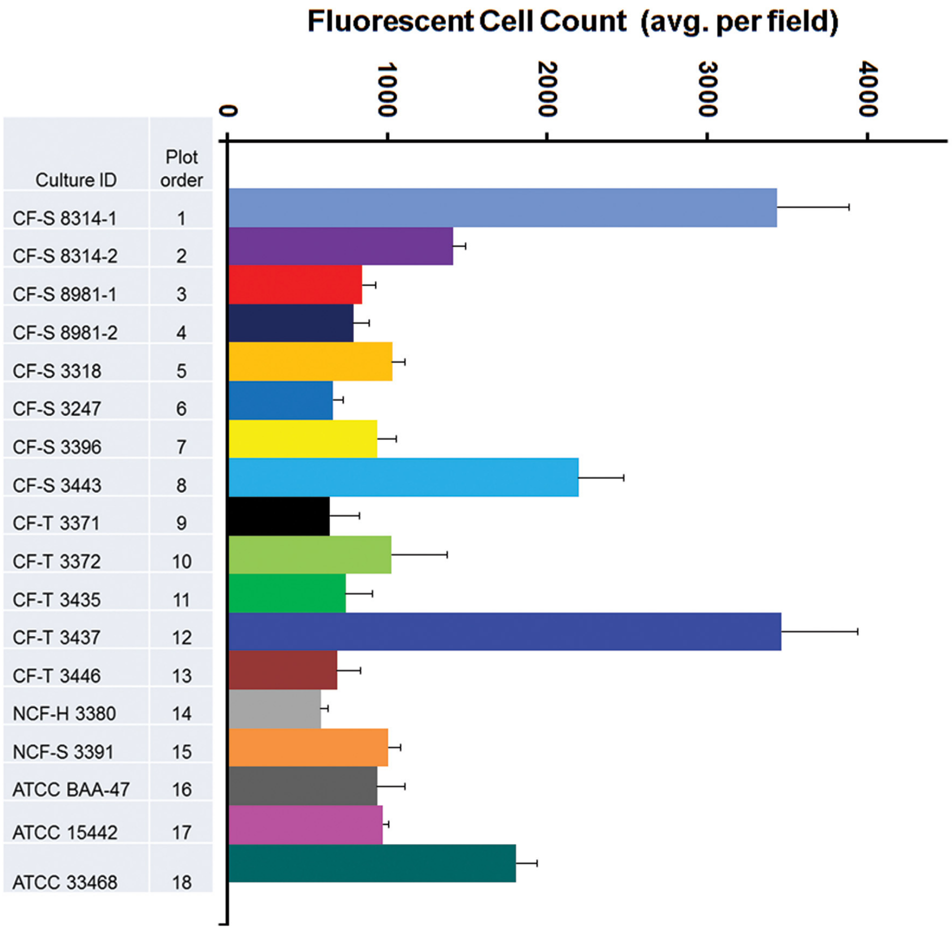

Figure S2.4. Example of heterogeneity of binding among *Pseudomonas aeruginosa* strains with regard to same glycopolymer, α-gal-PAA-Fluor, as evaluated by fluorescence microscopy. Binding assay plot depicts the average number of fluorescent cells per field of view for each specimen in this collection of *Pseudomonas aerginosa*, from clinical and laboratory sources, for assay with the multivalent fluorescent polyacrylamide-based glycopolymer with α-galactose as pendant monosaccharide. Range: lowest to highest ~ 600 – 3500 cells/field; aminoglucitol-PAA-Fluor negative control range (not depicted): 5-54 cells/field with fluorescence.

Materials and Methods S2

Bacteria

Pseudomonas aeruginosa clinical isolates described in Table III in the main text and Table S2.2 in this Supplementary Data, were obtained from cystic fibrosis and non-cystic fibrosis patient- derived specimens analyzed at the Diagnostic Laboratories of the University of Missouri Health Care Hospital and Clinics, Columbia, MO. Laboratory strains were acquired through the American Type Culture Collection (ATCC, Manassas, VA).

Identification and phenotypic analyses

Verification of identity of *Pseudomonas aeruginosa* was performed, and metabolic activity profiles were generated, with the VITEK2 automated ID system (BioMérieux, Durham, NC). Phenotypic observations followed culture on various solid agar media, including tryptic soy, MacConkey, *Pseudomonas* P and F agars, and blood agar (Remel, Lenexa, KS). Mucoid phenotypes of clinical isolates were maintained with minimal subculturing from original specimen frozen stocks, and incubation on MacConkey agar. Functional pili twitching assays was performed with Luria-Bertani agar (LB) using 1.5% agar (O'Toole and Kolter 1998).

Negative staining TEM for structural features presentation

Transmission electron microscopy (TEM) was performed on bacterial cultures following application of cultures in water to carbon-coated copper grids and on-grid staining with organotungsten (nano-W, Nanoprobes, Yaphank NY), as recently described (Chance and Mawhinney 2017). Images were acquired on a JEOL JEM-1400 Transmission Electron Microscope with DigitalMicroscope software (Gitan, Pleasanton, CA). TEM micrographs were adjusted for brightness & contrast using levels in Adobe Photoshop (Adobe, San Jose, CA).

The effects of the growth and handling conditions for the binding experiments on the structural and organizational features of the cultures was also examined. Bacteria to be prepared for TEM were taken from broth cultures with centrifugation prior to suspension in water, and from agar plate cultures suspended in water with no centrifugation step. Results: Upon negative staining and TEM analysis, flagella were found to be present under either growth and handling condition for 89% of the strains. Pili were observed for 78% of the strains, though for some isolates pili were very few in number and were more difficult to observe from centrifuged liquid culture preparations. When present, pili were typically found singly or as groups of several at one end of the bacterial cell, and unlike flagella, were not uniformly distributed among the cells in the culture.

Multivalent fluorescent glycopolymer binding profiles

Binding profiles of *Pseudomonas aeruginosa* cystic fibrosis isolates with multivalent fluorescent glycopolymers bearing pendant monosaccharides were performed by various means as described, and data reported, in the main text. An example of the microscopic data for α-gal-PAA-Fluor is also presented in Figure S2.4 for illustration of the level of heterogeneity of binding observed for a given sugar among the bacteria tested. *Pseudomonas* isolates or strains were identified as specific glycoconjugate "high binders" in fluorescence microscopy binding assay, as seen in Fig. 2B plot and in Fig. S2.4, when an average of greater than 1000 fluorescence- possessing cells per view, as enumerated by automated software detection, were present for the specific pendant monosaccharide fluorescent glycopolymer (PAA-Fluor).

References

O'Toole GA and Kolter R. 1998. Flagellar and twitching motility are necessary for *Pseudomonas aeruginosa* biofilm development. *Mol Microbiol*. 30:295–304.

Chance DL, Mawhinney T. 2017. Using Negative Staining TEM to Study Structure/Function Relationships of Cystic Fibrosis Host-Adapted Opportunistic Pathogen *Pseudomonas aeruginosa*. *Microsc and Microanal* 2017 S1:1354-1355.
