# Supplementary material for: Insights on Pseudomonas aeruginosa Carbohydrate Binding from Profiles of Cystic Fibrosis Isolates Using Multivalent Fluorescent Glycopolymers Bearing Pendant Monosaccharides
Source: Microorganisms. 2024 Apr 16;12(4):801. doi: 10.3390/microorganisms12040801 (PMC11051836; doi:10.3390/microorganisms12040801)
Supplement: Supplementary file 1 [file microorganisms-12-00801-s001.zip › Supplemental File S3.pdf]

Insights on *Pseudomonas aeruginosa* Carbohydrate Binding from Profiles of Cystic Fibrosis Isolates using Multivalent Fluorescent Glycopolymers Bearing Pendant Monosaccharides

Deborah L. Chance, Wei Wang, James K. Waters, Thomas P. Mawhinney
University of Missouri, Columbia, MO, 65211, USA

Supplemental File S3

Additional Binding Results

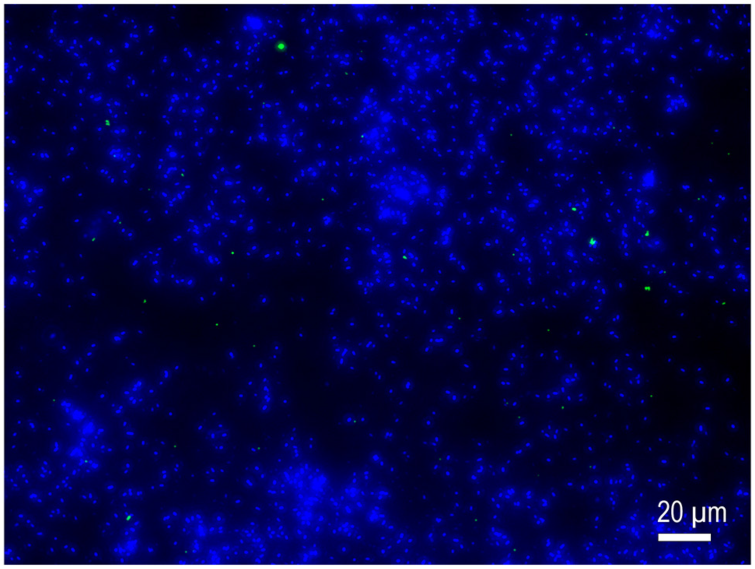

Figure S3.1. Fluorescence micrograph of minimally washed bacterial-fluorescent glycopolymer suspension in binding assay. Minor increase in bacterial-glycopolymer binding was observed for ATCC BAA47 with the α-Gal-PAA-Fluor when the bacteria were not washed extensively following the binding reaction (i.e. ~ 2% rather than ~ 1% in standard assay). In this example, an aliquot of the bacterial-glycopolymer binding reaction, following 2 h incubation, was applied to a glass slide, air-dried, and the unbound materials removed by one rinse with PBS. The specimen was then subjected to fluorescence microscopy.

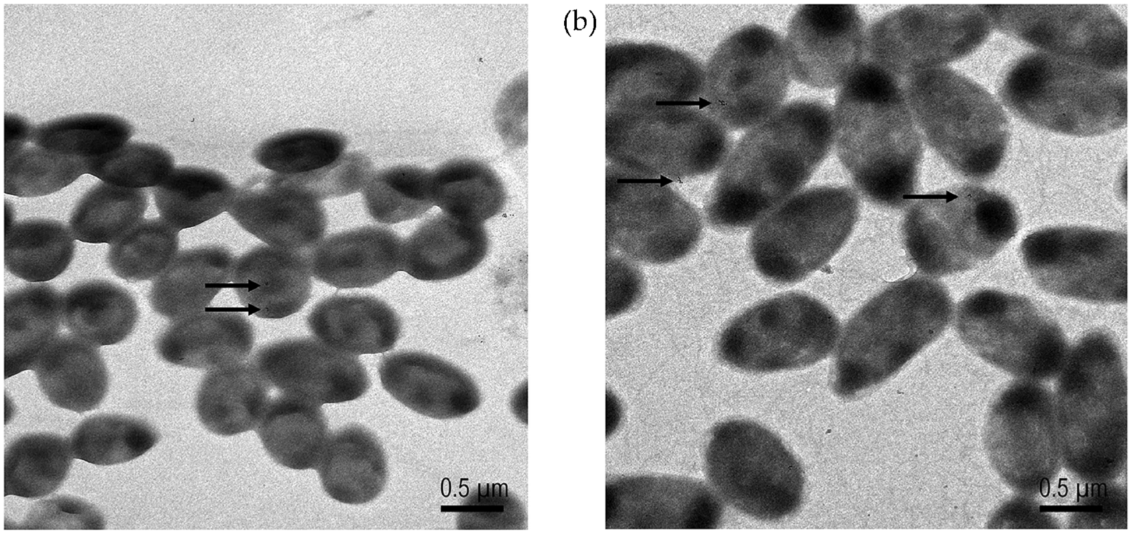

Figure S3.2. Transmission electron micrographs of gold-conjugates of fluorescein-immunolabeled β-Gal-PAA-Fluor minimally bound to *P. aeruginosa* clinical isolate CF-S 8314-1 (a) and laboratory strain ATCC BAA47 (b). Following binding tests, the bacterial suspensions deposited on Formvar carbon-coated nickel grids were incubated with gold conjugated anti-fluorescein isothiocyanate antibody. Only scarce gold-labeling was observed in both strains' tests with the β-galactose glycopolymer (arrowheads). These data are consistent with microscopic and spectroscopic analyses showing relatively small amounts of binding of β-Gal-PAA-Fluor to the various *P. aeruginosa* strains.
