# Supplementary material for: Insights on Pseudomonas aeruginosa Carbohydrate Binding from Profiles of Cystic Fibrosis Isolates Using Multivalent Fluorescent Glycopolymers Bearing Pendant Monosaccharides
Source: Microorganisms. 2024 Apr 16;12(4):801. doi: 10.3390/microorganisms12040801 (PMC11051836; doi:10.3390/microorganisms12040801)
Supplement: Supplementary file 1 [file microorganisms-12-00801-s001.zip › Supplemental File S4.pdf]

Insights on *Pseudomonas aeruginosa* Carbohydrate Binding from Profiles of Cystic Fibrosis Isolates using Multivalent Fluorescent Glycopolymers Bearing Pendant Monosaccharides

Deborah L. Chance, Wei Wang, James K. Waters, Thomas P. Mawhinney
University of Missouri, Columbia, MO, 65211, USA

Supplemental File S4
Lectin Detection

Mass spectrometry (MS) detection of *Pseudomonas aeruginosa* lectins LecA (PA-IL) and LecB (PA-IIL)

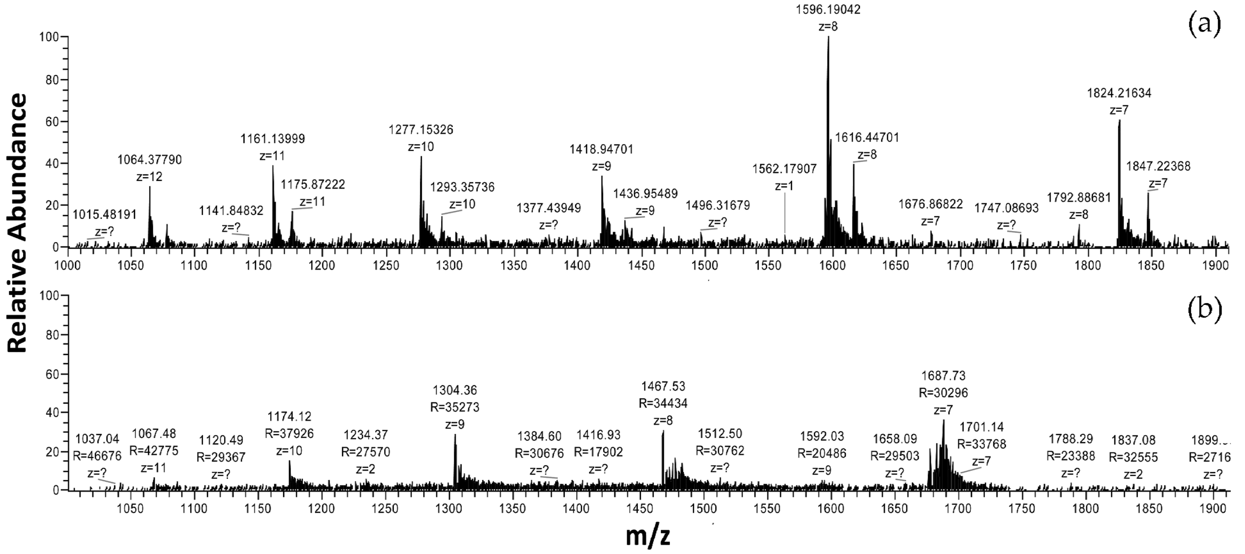

Figure S4. Electrospray ionization (ESI) positive ion mode mass spectra of LecA (a) and LecB (b) isolated from *P. aeruginosa* CF-S 8314-1.

Table S4. Mass spectral data, theoretical and observed, for lectins isolated from *P. aeruginosa* clinical isolate CF-S 8314-1 and authentic standards of *Pseudomonas* lectins LecA (PA-IL) and LecB (PA-IIL)

	Mass (*m/z*)		
Number of charges	Theoretical [a] for LecA standard [b]	Observed from LecA standard [b]	Observed from isolated LecA
7	1823.29	1824.22	1824.22
8	1595.38	1596.19	1596.19
9	1418.11	1418.95	1418.84
10	1276.3	1277.15	1277.15
11	1160.27	1161.14	1161.14
12	1063.58	1064.38	1064.38
**Deconvoluted MW**	12,763 Da	12,761 Da	12,761 Da

	Mass (*m/z*)		
Number of charges	Theoretical [a] for LecB standard [c]	Observed from LecB standard [c]	Observed from isolated LecB
6	1955.33	1956.19	1956.17
7	1676	1676.88	1677.01 & 1687.73
8	1466.5	1467.39	1467.53
9	1303.56	1304.57	1304.36
10	1173.2	1174.12	1174.12
11	1066.55	1067.38	1067.48
**Deconvoluted MW**	11,732 Da	11,732 Da	11,732 Da

[a] Theoretical masses were calculated based on precise molecular masses of purified LecA (PA-IL) and LecB (PA-IIL) from ATCC 33347 as determined by MALDI-TOF mass spectrometry. Reference: Gilboa-Garber N, Katcoff DJ, Garber NC, 2000. Identification and characterization of *Pseudomonas aeruginosa* PA-IIL lectin gene and protein compared to PA-IL. *FEMS Immunol. & Med. Microbiol.* 29:53-57. Deconvoluted molecular weights were derived with the Xtract feature of Xcalibur software (Thermo N.A.,Thermo Fisher Scientific, Waltham, MA).

[b] Commercial source: Sigma-aldrich, St. Louis, Missouri, USA

[c] Commercial source: Elicityl SA, France

Materials and Methods S4

Mass spectral analysis of lectin preparations from the *Pseudomonas aeruginosa* clinical isolate CF-S CF8314-1 was performed on a LTQ Orbitrap XL Mass Spectrometer (Thermo Fisher Scientific, Waltham, MA, *formerly Thermo North America*) equipped with an electrospray ionization (ESI) source. Commercially available proteins *P. aeruginosa* LecA, also known as PA-IL (Sigma, USA) and LecB, also known as PA-IIL (Elicityl, France), were employed as standards and similarly ionized and mass spectra analyzed.

Bacterial culture and lectin isolation procedures are detailed in the main text. Briefly, bacteria were grown in minimal media, the cells pelleted and sonicated, and the soluble materials exposed to and eluted from α-D-galactose- and α-D-mannose- agarose beads (with melibiose and 4-nitrophenyl α-L-fucopyranoside, respectively). The eluted specimens were prepared for mass spectral analysis by passage of 10 μL aliquots through desalting columns into a protein solution of 10 μg/mL lysozyme and 10 μg/mL BSA in 0.1% formic acid.

Specimens were then diluted as needed and directly infused into ESI source via a 10ul loop injection into the flow path established with the direct infusion syringe system. The ESI source solvent flow was set to 3 μL/min with an initial mobile phase of acetonitrile and water (40:60, v/v) containing 0.1 % formic acid, then at 12 min the mobile phase was changed to acetonitrile and water (80:20, v/v) containing 0.1% formic acid, and spray continued for another 12 min.

Conditions for ionization and collecting the positive ion spectra were as following: source voltage, 4.5 kV; capillary temperature, 275 ˚C; capillary voltage, 35 V; and tube lens, 100 V. The accurate mass detector was programmed to perform full scans with spectra recorded in the range of *m/z* 150 to *m/z* 2000, at resolution of 60,000.

Spectra of authentic standards were likewise infused and data recorded with Xcalibur software (Thermo Fisher Scientific, Waltham, MA). Deconvolution of multiply charged species was performed by Xtract feature of the software using the +8 species.

Technical note regarding these hydrophobic proteins (~ 40% by amino acid analysis) collected from affinity beads: We noticed that these lectins, as hydrophobic proteins, were easily lost to walls of tubes, vials, etc and found that incorporation of 10 μg/mL lysozyme (MW ~ 14kDa) and at 10 μg/mL BSA (MW ~ 64 kDa) in aqueous solution with 0.1% formic acid as carrier/blocking solution for desalted specimens or lectin standards in vials reduced sample loss. Running this lysozyme/BSA mix as background solution before sample introduction into the electrospray ionization source, provided better sample signal (than without protein carrier) with ionization and detection of several multiply charged species for each lectin. The +8 species were commonly strong signals for both lectins, and deconvolution of the isotopic envelope around these masses permitted ready determination of the molecular weights.
