# Supplementary material for: Insights on Pseudomonas aeruginosa Carbohydrate Binding from Profiles of Cystic Fibrosis Isolates Using Multivalent Fluorescent Glycopolymers Bearing Pendant Monosaccharides
Source: Microorganisms. 2024 Apr 16;12(4):801. doi: 10.3390/microorganisms12040801 (PMC11051836; doi:10.3390/microorganisms12040801)
Supplement: Supplementary file 1 [file microorganisms-12-00801-s001.zip › Supplemental File S5.pdf]

Insights on *Pseudomonas aeruginosa* Carbohydrate Binding from Profiles of Cystic Fibrosis Isolates using Multivalent Fluorescent Glycopolymers Bearing Pendant Monosaccharides

Deborah L. Chance, Wei Wang, James K. Waters, Thomas P. Mawhinney
University of Missouri, Columbia, MO, 65211, USA

Supplemental File S5

Probing *P. aeruginosa* Characteristics for Correlations with Enhanced Binding

Table S5.1. Features of *P. aeruginosa* in collection sorted by source

Respiratory or Other Source	Strains with Phenotype			Strains with Structural Feature(s) by TEM, n		
	**Source (n)**	**Phenotype**	**Total, n**	**F+, P+**	**F+ , P-**	**F-, P+**
Respiratory	CF Sputum (8)	NonMucoid	3	2	1	0
		Mucoid	3	2	1	0
		Motile	2	2	0	0
	CF Throat (5)	NonMucoid	1	0	1	0
		Mucoid	1	1	0	0
		Motile	3	3	0	0
	NCF Sputum (2)	NonMucoid	0	n.a.	n.a.	n.a.
		Mucoid	1	0	0	1
		Motile	1	0	0	1
Other	Other (3)	Nonmotile	0	n.a.	n.a.	n.a.
		Mucoid	0	n.a.	n.a.	n.a.
		Motile	3	2	1	0

Abbreviations: F+, flagella positive; F-, flagella not observed; P+, pili observed at least occasionally; P-, pili not observed; n, number of strains with the characteristic; n.a., not applicable as no strains in the category to analyse for the feature.

Table S5.2. *P. aeruginosa* "high binder" glycopolymers with features of high binding strains [1,2]

Glycopolymer	% of PA in collection (n)	Source or Feature[3] Frequency % of Higher Binding Strains, (*n*)						
		Respiratory	NonMucoid	Mucoid	Motile	F+, P+	F+ , P-	F-, P+
α-Gal	44% (8)	100% (8)	25% (2)	38% (3)	38% (3)	63% (5)	13% (1)	25% (2)
β-GalNAc	56% (10)	100% (10)	40% (4)	30% (3)	30% (3)	70% (7)	20% (2)	10% (1)
β-Gal3S	33% (6)	83% (5)	33% (2)	17% (1)	50% (3)	83% (5)	0% (0)	17% (1)
α-Neu5Ac	11% (2)	100% (2)	50% (1)	0% (0)	50% (1)	100% (2)	0% (0)	0% (0)

[1] Glycopolymers in this study revealed as "high binders" for 1 or more *P. aeruginosa* in this collection included the polyacrylamide-based multivalent fluoresceinated glycopolymers with pendant monosaccharides of galactose (Gal), N-acetylgalactosamine (GalNAc), galactose -3-O-sulfate (Gal3S), or sialic acid (Neu5Ac). [2] "High Binding" status was denoted in binding assays where enumeration of fluorescent glycopolymer-bound *P. aeruginosa* was determined microscopically, and there were an average of > 1000 fluorescent cells per microscopic field analyzed. [3] Abbreviations: F+, flagella positive; F-, flagella not observed; P+, pili observed at least occasionally; P-, pili not observed; n, number of strains with the characteristic.

Table S5.3. *P. aeruginosa* high binding glycopolymers data from Table 3 sorted by colony phenotype

Isolate or Strain Source	Culture ID (#)		Colony Phenotype	Structural Features Confirmed	Glycopolymer Binding Observed Microscopically as Fluorescent Cells/Field, and Spectrometrically as Fluorescence Intensity (AU) > 1000 cells (avg)	> 6000 AU
**CF Sputum**	CF-S 8314-1	1	nonmucoid	flagella, pili	α-Gal, β-GalNAc, β-Gal3S, α-Neu5Ac	α-Gal, β-GalNAc, β-Gal3S, α-Neu5Ac
**CF Sputum**	CF-S 8981-1	3	nonmucoid	flagella	β-GalNAc	α-Gal, β-GalNAc, β-Gal3S
**CF Sputum**	CF-S 3443	8	nonmucoid, (SCV)	flagella, pili	α-Gal, β-GalNAc, β-Gal3S	α-Gal, β-GalNAc, β-Gal3S, α-Neu5Ac
**CF Throat**	CF-T 3371	9	nonmucoid	flagella	β-GalNAc	α-Gal, β-GalNAc, β-Gal3S
**CF Sputum**	CF-S 8314-2	2	mucoid	flagella	α-Gal	α-Gal, β-GalNAc, β-Gal3S, α-Neu5Ac
**CF Sputum**	CF-S 8981-2	4	mucoid	flagella, pili	-	α-Gal, β-GalNAc, β-Gal3S, α-Neu5Ac
**CF Sputum**	CF-S 3396	7	mucoid	flagella, pili	β-GalNAc, β-Gal3S	α-Gal, β-GalNAc, β-Gal3S
**CF Throat**	CF-T 3372	10	mucoid	flagella, pili	α-Gal, β-GalNAc	α-Gal, β-GalNAc, β-Gal3S, α-Neu5Ac
**Non-CF Sputum**	ATCC 33468	18	mucoid	pili	α-Gal, β-GalNAc	α-Gal, β-GalNAc, β-Gal3S
**CF Sputum**	CF-S 3318	5	motile	flagella, pili	α-Gal	α-Gal, β-GalNAc, β-Gal3S
**CF Sputum**	CF-S 3247	6	motile	flagella, pili	-	α-Gal, β-GalNAc, β-Gal3S
**Non-CF Sputum**	NCF-S 3391	15	motile	pili	α-Gal, β-Gal3S	α-Gal, β-GalNAc, β-Gal3S, α-Neu5Ac
**CF Throat**	CF-T 3435	11	motile	flagella, pili	β-GalNAc	α-Gal, β-GalNAc, β-Gal3S
**CF Throat**	CF-T 3437	12	motile	flagella, pili	α-Gal, β-GalNAc, β-Gal3S, α-Neu5Ac	α-Gal, β-GalNAc, β-Gal3S, α-Neu5Ac
**CF Throat**	CF-T 3446	13	motile	flagella, pili	β-GalNAc	α-Gal, β-GalNAc, β-Gal3S
**Other**	NCF-H 3380	14	motile	flagella, pili	β-Gal3S	α-Gal, β-GalNAc, β-Gal3S
**Other**	ATCC BAA-47	16	motile	flagella	-	α-Gal, β-GalNAc, β-Gal3S
**Other**	ATCC 15442	17	motile	flagella, pili	-	α-Gal, β-GalNAc, β-Gal3S
