# Supplementary material for: Insights on Pseudomonas aeruginosa Carbohydrate Binding from Profiles of Cystic Fibrosis Isolates Using Multivalent Fluorescent Glycopolymers Bearing Pendant Monosaccharides
Source: Microorganisms. 2024 Apr 16;12(4):801. doi: 10.3390/microorganisms12040801 (PMC11051836; doi:10.3390/microorganisms12040801)
Supplement: Supplementary file 1 [file microorganisms-12-00801-s001.zip › Supplemental File S6.pdf]

Insights on *Pseudomonas aeruginosa* Carbohydrate Binding from Profiles of Cystic Fibrosis Isolates using Multivalent Fluorescent Glycopolymers Bearing Pendant Monosaccharides

Deborah L. Chance, Wei Wang, James K. Waters, Thomas P. Mawhinney
University of Missouri, Columbia, MO, 65211, USA

Supplemental File S6

Additional Details and Discussions on the Characteristics of CF Airway and *P. aeruginosa* which may Affect *P. aeruginosa* Survival and Success of Adjunctive Anti-Adhesive Therapeutics

Contents

S6.1. Complex and Variable Airway Milieu in Cystic Fibrosis (CF) Patients
S6.2. Character of the CF Airway May Impact Bacterial Colonization
S6.3. *Pseudomonas* Biofilm Exopolysacharide and Adhesin Interactions may Provide Targets for Adjunctive Therapeutics
S6.4. Overview of Findings and Contributions of *P. aeruginosa* Carbohydrate Binding Profiles Data toward Interventional Agents
S6.5. Significant Additional Factors which may Influence *P. aeruginosa* Carbohydrate Binding *in vivo*
S6.6. Bacterial Subpopulations may be Particularly Important for Persistence of CF Chronic Infections
S6.7. Specifics of Some of the Many Opportunities for *P. aeruginosa* Carbohydrate Interactions in the CF Airway
S6.8. Addressing Specific Types of Carbohydrate Related Intra-Biofilm Interactions for Therapeutic Interventions
S6.9. Drug Delivery Challenges and Opportunities in the CF airway Mucus Environment
S6.10. To Conclude these Additional Details and Discussions on the Characteristics of CF Airway and *P. aeruginosa* which may Affect *P. aeruginosa* Survival and Success of Adjunctive Anti-Adhesive Therapeutics
Technical Note
References - #'d as in Main Text
References - #s in Supplemental File S6 only
References - Additional Relevant Resources

S6.1. Complex and Variable Airway Milieu in Cystic Fibrosis (CF) Patients

Toward development of effective, novel CF *P. aeruginosa* anti-infectives, it is important to realize that *Pseudomonas* colonization is set in the complex airway milieu that results in part from host-specific genetics [4,5,10,15,s1,s2] and in part from the presence and activities of normal respiratory flora and an array of opportunistic pathogens [1,8,11,19,26,48,s3,s4,s5]. Mutations in the DNA encoding the cystic fibrosis transmembrane conductance regulator (CFTR) in the host exocrine systems lead to insufficient quantity and/or function of this protein at the cell surface [2,4-6,s1]. The severity of disease symptoms differs widely in the CF population, often reflecting the specific mutations present and the corresponding level of functional CFTR [4-6,s1]. Respiratory tract consequences of CFTR-deficits include mucous secretions with: inappropriate levels of pH, salt, and water; altered amounts and structures of high molecular weight mucous glycoproteins; and significant amounts of host immune cell-derived DNA, cell debris, lipids, and cations [2,6,10,43,49,s2]. Impaired mucociliary clearance, increased inflammation,

chronic obstruction and chronic infection are hallmarks of CF pulmonary disease [1,5,15,45,48]. Recently, CF-specific therapeutics that address the basic genetic defect at the cellular level, called CFTR modulators, have become available to individuals with mild to moderate CF disease [s1,4,5]. Many of these patients are experiencing markedly improved airway function and quality of life [s1,4-6]. Despite this therapeutic progress, lung infection with *Pseudomonas* and other pathogens remains a critical issue to address for patients with long-term symptomatic disease and/or severe lung damage, and for individuals for whom these drugs are not effective, not tolerated, or not available [2,4,6,27,48].

S6.2. Character of the CF Airway May Impact Bacterial Colonization

A caveat and context to keep in mind for anti-biofilm and anti-adhesion therapeutics development, is how the character of the CF airway may impact bacterial colonization. Typically, bacterial colonization of a site begins with attachment to a surface then proceeds to the development of a biofilm community [7,12,26,27,42,64,s6,s7]. In the CF respiratory tract insufficient mucociliary clearance may itself begin the aggregation and colonization process as bacteria are trapped in the viscous, dehydrated, often stationary, epithelial mucus gel [6,9,45,49,65,66,67,s8]. *P. aeruginosa* in this highly charged matrix may be temporarily protected from immune components and therapeutics [s8], and be permitted time to adapt to the new environment to further protect themselves, and/or to migrate to the underlying host cell-bound glycoconjugates (mucins, glycosphingolipids, proteoglycans) and invade the epithelium [21,24,36,48,68]. Further complicating potential anti-biofilm therapeutics is the nearly diagnostic presence in chronic CF infections of mucoid *P. aeruginosa* phenotype with abundant elaboration of the polysaccharide alginic acid (an anionic polymer composed of repeating units of acetylated β-1,4-linked D-mannuronic and L-guluronic acids) [7,19,27,s6,s7]. Alginic acid may impact bacterial adherence to mucin oligosaccharides [1,26,s9], increase the viscosity of airway secretions, affect drug diffusion, impair the already-compromised mucociliary clearance, and protect encased organisms (whether producers or not) from dehydration, antibiotics, phagocytosis and killing by host immune cells [1,26,48,s7,s10,s11,s12,s13].

S6.3. *Pseudomonas* Biofilm Exopolysacharide and Adhesin Interactions may Provide Targets for Adjunctive Therapeutics

With the biofilm central for *Pseudomonas* survival in so many environments [6,7,20,27,53, s6,s7,s10,s14] investigations focused on the interactions within the biofilm matrix have also shed light to suggest potential new targets for CF anti-adhesion and anti-aggregation therapeutics [27,89,130,s14]. For example, LecA and LecB were found to associate with two bacterial-derived exopolysaccharides (EPS) Pel and Psl to help build and maintain the biofilm integrity [22,89]. *P. aeruginosa* Pel was characterized as a cationic polymer composed of *N*-acetylglucosamine (GlcNAc) and *N*-acetylgalactosamine (GalNAc) [24,89,136]. In CF, activity of the bacterial enzyme PelA was shown to alter the degree of Pel acetylation and thereby its positive charge and ability to limit diffusion of charged antibiotics [24,136]. Psl was described as a neutral polymer rich in mannose, glucose, and rhamnose [24,27,89,136]. Using *P. aeruginosa* from various sources, investigations found that for strains with either Pel or Psl as the dominant EPS, the fimbrial adhesin CdrA was also significant in bacterial aggregation and biofilm development [136]. *Pseudomonas* aggregates with Pel and/or Psl were recently demonstrated in CF sputa via immunohistochemical visualizations, showing that their existence *in vivo* is clinically relevant [24], and that both aggregate size and matrix EPS composition varied among CF patients [24]. Additionally, *in vitro* analyses revealed that both partially deacetylated Pel and Psl could associate with extracellular DNA (eDNA) and shield the DNA from therapeutic DNase [24,s15]. These data, and earlier data from others, suggested that aggregated and/or biofilm-growing *Pseudomonas in vivo* may have the protection of a DNA coat or web [s15] as yet another physical means of tolerance to positively charged antibiotics such as tobramycin (as is often seen in CF) [24,27,s6].

S6.4. Overview of Findings and Contributions of *P. aeruginosa* Carbohydrate Binding Profiles Data toward Interventional Agents

In this study we evaluated the glycopolymer binding profile obtained for each isolate or strain relative to its source, phenotypic characteristics, and structural features (i.e. flagella, pili) to assess for informative patterns and predictive trends. α-D-galactose (αGal), β-D-*N*-acetylgalactosamine (βGalNAc), and β-D-galactose-3-sulfate (βGal3S) glycopolymers associated with all *P. aeruginosa* specimens, and this positive binding was accounted for by a small subpopulation within the cultures. Three CF isolates of varied phenotypes showed enhanced associations with αGal, βGalNAc, and βGal3S glycopolymers. Eleven other clinical strains showed this higher binding characteristic to one or two of these glycopolymers. While no phenotype or structural feature guaranteed enhanced binding to any of these glycopolymers, all CF throat isolates and all nonmucoid CF sputum isolates showed high binding to the βGalNAc glycopolymer. These insights, taken in the context of the complex CF airway milieu discussed above and in the main text, are anticipated to inform future anti-infective therapeutic strategies addressing host-pathogen interactions, bacterial aggregation, biofilm matrix development and disruption, and novel agent development for directed-targeting of antibiotics to CF airway *P. aeruginosa*.

S6.5. Significant Additional Factors which may Influence *P. aeruginosa* Carbohydrate Binding *in vivo*

The *P. aeruginosa* variability and fluidity in expression or lack of expression of virulence features and structures further complicates the development of targeted agents for interfering with airway colonization [10,11,15,20,24,26,29,36,58,63,88,90,116,117,131,136,s16]. Apparent microbial microevolution may reflect both reversible phenotypic switches and genetic alterations [13,15,16,19,32,34,135, s17,s18,s19,]. Such fluctuation may occur within a patient and within an original bacterial clone [11,16,32,135]. This flexibility and genetic diversity may in turn affect carbohydrate binding. Virulence structures like flagella, pili, lectins, extracellular polysaccharides (i.e. alginate, Psl, Pel), and other cell-associated and secreted proteins (i.e. CdrA), may separately or coordinately contribute to both *Pseudomonas*-host interactions and to bacterial community assembly [64]. Better understanding of the bacterial carbohydrate interactions that variably partake in colonization and in formation, stabilization, and dispersal of the protected aggregates and complex biofilm architectures will be important to enhancing anti-infective therapeutics.

The *P. aeruginosa* use of redundant and/or complementary mechanisms for attachment, colonization and survival [12,44,131,s6,s11,s20] should be respected in the design of new interventional agents. The presence of mixed phenotype variants of *P. aeruginosa* within a sputum sample is common in CF [9,10,16]. Localized co-existence of non-mucoid and mucoid PA *in vivo* may permit each to benefit from the others' adaptations for surviving the CF host and bacterial competitors [16,19,27,s13,s18, s20,]. Variants, within a biofilm for example, may divide the labors, share the public goods, and jointly evade the host immune effectors [1,20,64,131,142,s13,s21]. Involvement in mixed species biofilms in CF may further benefit *P. aeruginosa* [11,27,s4,s18,s21,s22]. The CF airway microbiome is often patient specific and its diversity is found to fluctuate and adjust over an individual's life [6,8,11,30,s5,48,s4,s21]. Shifts away from the normal microbiome may not only contribute to antibiotic tolerance, but also within polymicrobial communities bring about interspecies interactions that influence *P. aeruginosa* expression of carbohydrate binding features and thereby alter the effectiveness of adhesin-targeted therapies [s3,s21]. These realizations may improve the understanding and treatment of *P. aeruginosa* colonization and persistence in CF.

The conditions of the individual host may further affect the processes of colonization and adaptation of *P. aeruginosa* [1,9,20,31,34,82,83,133,s2,s3, s23]. Host condition is suggested to trigger the divergent evolutionary paths of *Pseudomonas* variants [16,19,34]. In addition to the altered chemistries of CF, the local inflammatory and infectious microenvironments, and the variably impaired mucociliary clearance, host status effectors also include pre-existing and progressing airway pathologies [20,23,34,83,133,s2,s3,s23]. The specific and variable characteristics of the complex carbohydrates present in CF respiratory tracts are mechanistically important to bacterial binding and/or clearance from the

airway [9,60,65,67,78,79,81,82]. Distinctions and differences are expected in mucosal glycoconjugates reflecting the host genetic background, the functional impacts of the specific CFTR mutations, the history of exacerbations, the degree of inflammation, the current infections, and the ongoing therapies [9,34,67,82,83,133,s2,s3,s23,s24]. Active viral or bacterial infections, for example, may also alter what host glycans *P. aeruginosa* adhesins may access. Neuraminidases from influenza viruses and/or bacteria may enhance opportunities for *P. aeruginosa* binding by removing sialic acids from epithelial and mucosal glycoconjugates to increase the density of cell surface asialo-glycosphingolids GM1 and/or GM2 and expose galactose on membrane-bound MUC1 mucin [9,79,85,145,s25]. *P. aeruginosa* sialidase itself is suggested to be important in biofilm formation [s25]. Sub-inhibitory concentrations of antibiotics may also elicit *P. aeruginosa* changes that affect mucin binding [88,137,138] and production of virulence factors, such as the cytotoxic phenazine pyocyanin [139]. Pyocyanin at non-lethal levels may increase airway epithelial cell production of specific types of sialyl-Lewis antigens, such as sialyl-LeX shown important to bacterial binding [67]. As might be seen with lytic virus infection or following other injurious conditions, wounded and regenerating epithelial margins undergoing remodeling are also anticipated to show increased amounts of asialo-glycosphinogolipids and other exposed glycocalyx components for *Pseudomonas* binding [60,68,148]. Increased cytotoxicity observed *in vitro* for the CF therapeutic agent colistimethate [48] when coincident with pyocyanin [140] suggests that inadvertent damage to the CF epithelium during antimicrobial treatment may also set the remodeling stage and provide even more sites for binding, aggregation, and biofilm establishment.

The well-known, well-respected, adhesive and protective biofilm provides much *Pseudomonas* heterogeneity that may affect host interactions and effectiveness of targeted anti-infective agents [6,7,14,21,23,26,27,40,42,45,130,136,s6,s7,s10,s17,s19,s26]. Proteins involved in biofilm formation and interactions, such as CdrA, LecA and LecB, show genetic variations [117,136,147]. The dominant exopolysaccharide (EPS) elaborated in *Pseudomonas* biofilms varies, as does the amount [10,18,130,136, s11]. Mucoid isolates typically produce an abundance of the acidic polysaccharide alginate 14,27,136], while other phenotypes elaborate the neutral matrix polysaccharide Psl, and/or the cationic pellicle EPS Pel [10,14,27,36, s11]. The airway biofilm structures and complexities, as seen upon analysis of sputa, also include variable and potentially large amounts of bacterial and neutrophil-derived extracellular DNA (eDNA) and other host elements [7,21,43,45,s2,s6]. Heterogeneous chemical gradients within bacterial biofilms foster microenvironments such that local adaptations of *Pseudomonas* cells are not equivalent across sister cells in other areas of the matrix [7,15,21,27,42,64,134,s10,s27,s26]. Varied distribution of therapeutic agents within the biofilm, as well as within the airway, may further modulate virulence [21,42,132,139,s26]. Sub-lethal levels of antimicrobials within the biofilm can lead to the generation of non-replicating antibiotic tolerant persister subpopulations and may include a phenotypic shift to the small colony variant morphology [7,18,20,21,s10,s17]. Such regional biological flexibility is expected to contribute to *Pseudomonas* survival.

With all these environmental influences and *P. aeruginosa*'s adept adaptive abilities the rational design of agents to universally limit this opportunistic pathogen's negative effects on its host seems quite the daunting challenge. However, results of this carbohydrate binding profile study of a heterogeneous collection of *P. aeruginosa* may shed light on common ground from which to build broadly effective adjuvants to current therapies.

S6.6. Bacterial Subpopulations may be Particularly Important for Persistence of CF Chronic Infections

The concept of subpopulations providing the desired function for the whole population is a familiar theme for microorganisms [1,64,132,142,143,s19,s26]. Examples in CF include portions of infecting populations persisting as metabolically inactive cells tolerant to antibiotic challenges (such as small colony variants), nonmucoid organisms living among mucoid variants protected from dehydration, antibiotics and host recognition, and metabolically deficient cells surviving as social cheaters benefitting from the activities of the larger community [1,6,8,14,21,64,132,142,s10,s18,s19,s25,s26, s27]. For opportunistic pathogens like *P. aeruginosa*, whether division of labor is intentional by species design, is driven by adaptation to oxygen level, sub-inhibitory antibiotic(s) or other chemical gradient(s) or

signal(s), or is generated stochastically (randomly), the heterogeneity of character among cells undoubtedly contributes to species survival and chronic colonization of the vulnerable host [1,8,11,21,42,64,132,142,s10,s18,s19,s25,s26,s28].

S6.7. Specifics of Some of the Many Opportunities for *P. aeruginosa* Carbohydrate Interactions in the CF Airway

In the CF airway, *P. aeruginosa* likely participate in numerous complex carbohydrate interactions at epithelial surfaces [148], within respiratory mucus, and in colonizing bacterial aggregates and biofilm community matrices.

For the host, the terminal and penultimate carbohydrate residues and their linkages impose important definitive characteristics which may influence bacterial association, including blood group specificity [9,49,74,s18,s24]. Structures common to respiratory mucin oligosaccharides are the terminal, and often repeating internal, disaccharide units of galactose linked to *N*-acetyl-glucosamine as βGal(1-3)βGlcNAc or βGal(1-4)βGlcNAc, with or without additional sialic acid, fucose or sulfate entities [9,49,72,74,83,84,149,s24]. Terminal structures common to host cell gangliosides include galactose and *N*-acetyl-galactosamine in linkages of βGal(1-3)βGalNAc (as in asialo-GM1) and GalNAcß1-4(NeuAcα2-3)Galß1-4Glcß-Cer (as in GM2), with the series of glycosphingolipids carrying varying numbers of sialic acids [77,s9,s29]. As noted above, host conditions may affect what complex carbohydrates are elaborated and how they are modified [9,34,67,82,83,133,s3,s23,s24]. Where disease, damage, infection, and/or inflammation occur, the relative amounts and locations of sialic acids, fucose, and sulfate esters are anticipated to vary [9,49,74,100,s24].

The pendant monosaccharide structures positive in this study for whole cell binding of *P. aeruginosa* may be found in nature as follows: the α-Gal structure can be found at the termini of carbohydrate residues of blood group B active oligosaccharides and globoseries glycosphingolipid Gb3 on plasma membranes; the ß-GalNAc at termini of gangliosides like GM2; and the ß-Gal3S on CF tracheobronchial mucins oligosaccharides [47,49,147,149,s29,s30]. Of note, in branched CF respiratory mucin oligosaccharides, it is also possible to have the blood group B antigen at the terminus of one branch and a ß-Gal3S residue at the terminus of another branch [49], positioning two binding epitopes in potentially close proximity.

For anti-adhesion therapeutics, the first thought may be to address this host attachment. In CF however, the concentration of organisms per location in the human host may be more related to stagnant mucus trapping and protective bacterial cell-bacterial cell interactions with aggregation and biofilm community structure development than to the numbers of available host cell receptors for the bacteria. Each type of interaction may provide sites for anti-adhesion related anti-infective interventions. As noted above, it should also be respected in therapeutics design that, as discussed above, the varied permutations of the condition of the host, the adaptations of the colonizing microbes, and the treatments being received are likely to keep the infectious landscape ever-changing [8,20,43,133,s24,s31].

S6.8. Addressing Specific Types of Carbohydrate Related Intra-Biofilm Interactions for Therapeutic Interventions

It is increasingly appreciated that establishment of chronic *P. aeruginosa* colonization in the CF host likely includes persistence in a protective biofilm. Prevention and destabilization of *Pseudomonas* biofilms *in vivo* via interventions addressing simple sugars interactions with cell surface and biofilm bound adhesins is an enticing avenue of investigation.

While this study did not address biofilms directly, the binding results appear translatable to this arena. As highlighted above: extracellular matrices of *P. aeruginosa* vary between patients in the type and amounts of EPS [24]; LecB may bind mannose side chains in Psl [89]; CdrA may associate with *N*-acetyl-galactosamine in Pel biofilms [24,136]; and glycomimetic inhibitors may interfere with bacterial outer membrane bound [90,92] or biofilm located lectins interactions to disperse intact *Pseudomonas* biofilms

[104,106]. Combining these findings and many others with the insights of the current whole cell glycopolymer binding study suggests that combinations of inhibitors designed to affect (a) LecA binding to galactose and a lesser degree *N*-acetylgalactosamine [47,96], (b) LecB affinity for fucose and mannose, and/or (c) fibrillar protein CdrA association with *N*-acetylgalactosamine, may jointly affect biofilm integrity *in vivo* for a variety of individuals colonized with *P. aeruginosa*.

S6.9. Drug Delivery Challenges and Opportunities in the CF airway Mucus Environment

The highly charged and dense natures of both the CF airway mucus environment and the *P. aeruginosa* biofilm emphasize the challenges of limited diffusion and reduced penetration of charged therapeutics en route to their bacterial targets [6-8,21,26,27,40,42,43,45,48,52,130]. Designing new adjunctive agents and their delivery solutions, especially for inhaled preparations, to remain as neutral and as small as possible will likely aid in their successful distribution and deposition [40]. The smaller sized carbohydrate-based therapeutics would also be less likely to elicit a host immune response to the agent. Further investigation is required to assess whether small relatively neutral molecules possessing galactose and/or *N*-acetyl-galactosamine in the appropriate buffer conditions may penetrate and diffuse through the airway mucus and the variable and potentially highly ordered complex biofilm matrices and channels, to compete with carbohydrate interactions at the bacterial cell membranes, at the host cell surfaces, and within the biofilms themselves.

Co-administration of aerosols of carbohydrate-based agents to disrupt bacterial interactions with proven antibiotics continues to represent a promising anti-*Pseudomonal* therapeutic strategy [37,46,52]. In an *in vitro* model of combination inhalation therapy, a nebulized ciprofloxacin-mannitol formulation demonstrated improved antibiotic effectiveness against a *Pseudomonas* biofilm [37]. This synergy and that suggested by early clinical reports of benefit of inhaled simple sugars to antibiotic treatments [100,101] imply that simultaneous application of antibiotic with a carbohydrate-based biofilm destabilizing agent is an actionable goal for adjunctive therapeutics [37,113]. This cooperative benefit may be realized regardless of the mechanism of action of biofilm destabilization, i.e. via interfering with lectin binding, by water influx as expected with therapeutic osmotic agents such as dry powder mannitol, via enzymatic disruption of the exopolysaccharide matrix or the eDNA within the biofilm, and etc [7,42,46,51,s12].

S6.10. To Conclude these Additional Details and Discussions on the Characteristics of CF Airway and *P. aeruginosa* which may Affect *P. aeruginosa* Survival and Success of Adjunctive Anti-Adhesive Therapeutics

These insights and data, taken in the context of the complex CF airway milieu, are anticipated to inform future anti-infective therapeutic strategies addressing host-pathogen interactions, bacterial aggregation, biofilm matrix development and disruption, and novel agent development for directed-targeting of antibiotics to CF airway *P. aeruginosa*.

Technical Note:

This bacterial carbohydrate binding series of experiments relied heavily on synthetic water soluble polyacrylamide-based fluorescent glycopolymers representing the pendant monosaccharides anticipated at termini of mucosal oligosaccharides either decorating airway mucins or epithelial cells.

Historically, synthetic glycopolymers/particles as experimental tools have taken many forms and been produced in many ways [105,108,s21,s23]. Functionalized glycopolymers have had widespread use in detecting pathogens and cancers, and more recently in research on targeting therapeutics [22,54,105,s28,s32,s33]. Fluorescence-tagged glycopolymers specifically have been proven versatile aides in investigations of host-pathogen interactions and bacterial lifestyles [22,65,75,81,s33].

Previous studies in our laboratories have shown the well-defined predictably uniform glycopolymers produced by controlled polymerization reactions can provide anomeric specificity important by understanding bacterial lectin binding [119].

References - #'d as in Main Text

1. Malhotra, S.; Hayes D., Jr.; Wozniak D.J. Cystic fibrosis and *Pseudomonas aeruginosa*: the host-microbe interface. *Clin. Microbiol. Rev.* **2019**, *32(3)*. DOI: 10.1128/CMR.00138-18.
2. Morrison, C.B.; Markovetz M.R.; Ehre C. Mucus, mucins, and cystic fibrosis. *Pediatr. Pulmonol.* **2019**, *54 Suppl*, S84-S96. DOI: 10.1002/ppul.24530.
3. Nichols, D.P.; Durmowicz A.G.; Field A.; Flume P.A.; VanDevanter D.R.; Mayer-Hamblett N. developing inhaled antibiotics in cystic fibrosis: current challenges and opportunities. *Ann. Am. Thorac. Soc.* **2019**, *16(5)*, 534-539. DOI: 10.1513/AnnalsATS.201812-863OT.
4. Somayaji, R.; Nichols D.P.; Bell S.C. Cystic fibrosis - Ten promising therapeutic approaches in the current era of care. *Expert. Opin. Investig. Drugs* **2020**, *29(10)*, 1107-1124. DOI: 10.1080/13543784.2020.1805733.
5. Girón Moreno, R.M.; García-Clemente M.; Diab-Cáceres L.; Martínez-Vergara A.; Martínez-García M.Á.; Gómez-Punter R.M. Treatment of pulmonary disease of cystic fibrosis: a comprehensive review. *Antibiotics* **2021**, *10(5)*. DOI: 10.3390/antibiotics10050486.
6. Manos, J. Current and emerging therapies to combat cystic fibrosis lung infections. *Microorganisms* **2021**, *9(9)*. DOI: 10.3390/microorganisms9091874.
7. Martin, I.; Waters V.; Grasemann H. Approaches to targeting bacterial biofilms in cystic fibrosis airways. *Int. J. Mol. Sci* **2021**, *22(4)*. DOI: 10.3390/ijms22042155.
8. Reece, E.; Bettio P.H.A.; Renwick J. Polymicrobial interactions in the cystic fibrosis airway microbiome impact the antimicrobial susceptibility of *Pseudomonas aeruginosa*. *Antibiotics (Basel)* **2021**, *10(7)*. DOI: 10.3390/antibiotics10070827.
9. Venkatakrishnan, V.; Packer N.H.; Thaysen-Andersen M. Host mucin glycosylation plays a role in bacterial adhesion in lungs of individuals with cystic fibrosis. *Expert. Rev. Respir. Med.* **2013**, *7(5)*, 553-576. DOI: 10.1586/17476348.2013.837752.
10. Jurado-Martin, I.; Sainz-Mejias M.; McClean S. *Pseudomonas aeruginosa*: an audacious pathogen with an adaptable arsenal of virulence factors. *Int. J. Mol. Sci* **2021**, *22(6)*. DOI: 10.3390/ijms22063128.
11. O'Brien, S.; Fothergill J.L. The role of multispecies social interactions in shaping *Pseudomonas aeruginosa* pathogenicity in the cystic fibrosis lung. *FEMS Microbiol. Lett.* **2017**, *364(15)*. DOI: 10.1093/femsle/fnx128.
12. Wagner, S.; Sommer R.; Hinsberger S.; Lu C.; Hartmann R.W.; Empting M., et al. Novel strategies for the treatment of *Pseudomonas aeruginosa* infections. *J. Med. Chem.* **2016**, *59(13)*, 5929-5969. DOI: 10.1021/acs.jmedchem.5b01698.
13. Nguyen, D.; Singh P.K. Evolving stealth: genetic adaptation of *Pseudomonas aeruginosa* during cystic fibrosis infections. *Proc. Natl. Acad. Sci. U. S. A* **2006**, *103(22)*, 8305-8306. DOI: 10.1073/pnas.0602526103.
14. Starkey, M.; Hickman J.H.; Ma L.; Zhang N.; De L.S.; Hinz A., et al. *Pseudomonas aeruginosa* rugose small-colony variants have adaptations that likely promote persistence in the cystic fibrosis lung. *J. Bacteriol.* **2009**, *191(11)*, 3492-3503. DOI: 10.1128/JB.00119-09.
15. Folkesson, A.; Jelsbak L.; Yang L.; Johansen H.K.; Ciofu O.; Hoiby N., et al. Adaptation of *Pseudomonas aeruginosa* to the cystic fibrosis airway: an evolutionary perspective. *Nat. Rev. Microbiol.* **2012**, *10(12)*, 841-851. DOI: 10.1038/nrmicro2907.
16. Workentine, M.L.; Sibley C.D.; Glezerson B.; Purighalla S.; Norgaard-Gron J.C.; Parkins M.D., et al. Phenotypic heterogeneity of *Pseudomonas aeruginosa* populations in a cystic fibrosis patient. *PLoS ONE* **2013**, *8(4)*, e60225. DOI: 10.1371/journal.pone.0060225.
17. Jorth, P.; Staudinger B.J.; Wu X.; Hisert K.B.; Hayden H.; Garudathri J., et al. Regional isolation drives bacterial diversification within cystic fibrosis lungs. *Cell Host. Microbe* **2015**, *18(3)*, 307-319. DOI: 10.1016/j.chom.2015.07.006.
18. Malone, J.G. Role of small colony variants in persistence of *Pseudomonas aeruginosa* infections in cystic fibrosis lungs. *Infect. Drug Resist.* **2015**, *8*, 237-247. DOI: 10.2147/IDR.S68214.
19. Winstanley, C.; O'Brien S.; Brockhurst M.A. *Pseudomonas aeruginosa* Evolutionary adaptation and diversification in cystic fibrosis chronic lung infections. *Trends Microbiol.* **2016**, *24(5)*, 327-337. DOI: 10.1016/j.tim.2016.01.008.
20. Rossi, E.; La R.R.; Bartell J.A.; Marvig R.L.; Haagensen J.A.J.; Sommer L.M., et al. *Pseudomonas aeruginosa* adaptation and evolution in patients with cystic fibrosis. *Nat. Rev. Microbiol.* **2021**, *19(5)*, 331-342. DOI: 10.1038/s41579-020-00477-5.
21. Ciofu, O.; Tolker-Nielsen T. Tolerance and resistance of *Pseudomonas aeruginosa* biofilms to antimicrobial agents-how *P. aeruginosa* can escape antibiotics. *Front Microbiol.* **2019**, *10*, 913. DOI: 10.3389/fmicb.2019.00913.
22. Limqueco, E.; Passos Da S.D.; Reichhardt C.; Su F.Y.; Das D.; Chen J., et al. Mannose conjugated polymer targeting *P. aeruginosa* biofilms. *ACS Infect. Dis.* **2020**, *6(11)*, 2866-2871. DOI: 10.1021/acsinfecdis.0c00407.
23. Trostrup, H.; Laulund A.S.; Moser C. Insights into host-pathogen interactions in biofilm-infected wounds reveal possibilities for new treatment strategies. *Antibiotics* **2020**, *9(7)*. DOI: 10.3390/antibiotics9070396.

24. Jennings, L.K.; Dreifus J.E.; Reichhardt C.; Storek K.M.; Secor P.R.; Wozniak D.J., et al. *Pseudomonas aeruginosa* aggregates in cystic fibrosis sputum produce exopolysaccharides that likely impede current therapies. *Cell Rep.* **2021**, *34(8)*, 108782. DOI: 10.1016/j.celrep.2021.108782.
25. Metelkina, O.; Huck B.; O'Connor J.S.; Koch M.; Manz A.; Lehr C.M., et al. Targeting extracellular lectins of *Pseudomonas aeruginosa* with glycomimetic liposomes. *J. Mater. Chem. B* **2022**, *10(4)*, 537-548. DOI: 10.1039/d1tb02086b.
26. Tuon, F.F.; Dantas L.R.; Suss P.H.; Tasca Ribeiro V.S. Pathogenesis of the *Pseudomonas aeruginosa* Biofilm: A Review. *Pathogens* **2022**, *11(3)*. DOI: 10.3390/pathogens11030300.
27. Chung, J.; Eisha S.; Park S.; Morris A.J.; Martin I. How three self-secreted biofilm exopolysaccharides of *Pseudomonas aeruginosa*, Psl, Pel, and alginate, can each be exploited for antibiotic adjuvant effects in cystic fibrosis lung infection. *Int. J. Mol. Sci* **2023**, *24(10)*. DOI: 10.3390/ijms24108709.
28. Mayer-Hamblett, N.; Rosenfeld M.; Gibson R.L.; Ramsey B.W.; Kulasekara H.D.; Retsch-Bogart G.Z., et al. *Pseudomonas aeruginosa in vitro* phenotypes distinguish cystic fibrosis infection stages and outcomes. *Am. J. Respir. Crit Care Med.* **2014**, *190(3)*, 289-297. DOI: 10.1164/rccm.201404-0681OC.
29. Kamath, K.S.; Pascovici D.; Penesyan A.; Goel A.; Venkatakrishnan V.; Paulsen I.T., et al. *Pseudomonas aeruginosa* cell membrane protein expression from phenotypically diverse cystic fibrosis isolates demonstrates host-specific adaptations. *J. Proteome. Res.* **2016**, *15(7)*, 2152-2163. DOI: 10.1021/acs.jproteome.6b00058.
30. Bacci, G.; Mengoni A.; Fiscarelli E.; Segata N.; Taccetti G.; Dolce D., et al. A different microbiome gene repertoire in the airways of cystic fibrosis patients with severe lung disease. *Int. J. Mol. Sci* **2017**, *18(8)*. DOI: 10.3390/ijms18081654.
31. Fernandez-Barat, L.; Ciofu O.; Kragh K.N.; Pressler T.; Johansen U.; Motos A., et al. Phenotypic shift in *Pseudomonas aeruginosa* populations from cystic fibrosis lungs after 2-week anti-*Pseudomonal* treatment. *J. Cyst. Fibros.* **2017**, *16(2)*, 222-229. DOI: 10.1016/j.jcf.2016.08.005.
32. Hansen, S.K.; Rau M.H.; Johansen H.K.; Ciofu O.; Jelsbak L.; Yang L., et al. Evolution and diversification of *Pseudomonas aeruginosa* in the paranasal sinuses of cystic fibrosis children have implications for chronic lung infection. *ISME J.* **2012**, *6(1)*, 31-45. DOI: 10.1038/ismej.2011.83.
33. Clark, S.T.; Diaz Caballero J.; Cheang M.; Coburn B.; Wang P.W.; Donaldson S.L., et al. Phenotypic diversity within a *Pseudomonas aeruginosa* population infecting an adult with cystic fibrosis. *Sci. Rep.* **2015**, *5*, 10932. DOI: 10.1038%2Fsrep10932.
34. Klockgether, J.; Cramer N.; Fischer S.; Wiehlmann L.; Tummler B. Long-term microevolution of *Pseudomonas aeruginosa* differs between mildly and severely affected cystic fibrosis lungs. *Am. J. Respir. Cell Mol. Biol.* **2018**, *59(2)*, 246-256. DOI: 10.1165/rcmb.2017-0356OC.
35. Fleming, D.; Rumbaugh K.P. Approaches to dispersing medical biofilms. *Microorganisms.* **2017**, *5(2)*. DOI: 10.3390/microorganisms5020015.
36. Curran, C.S.; Bolig T.; Torabi-Parizi P. Mechanisms and targeted therapies for *Pseudomonas aeruginosa* lung infection. *Am. J. Respir. Crit Care Med.* **2018**, *197(6)*, 708-727. DOI: 10.1164/rccm.201705-1043SO.
37. Loo, C.Y.; Lee W.H.; Lauretani G.; Scalia S.; Cipolla D.; Traini D., et al. Sweetening inhaled antibiotic treatment for eradication of chronic respiratory biofilm infection. *Pharmaceut. Res.* **2018**, *35(3)*, 50. DOI: 10.1007/s11095-018-2350-4.
38. Rezzoagli, C.; Archetti M.; Mignot I.; Baumgartner M.; Kummerli R. Combining antibiotics with antivirulence compounds can have synergistic effects and reverse selection for antibiotic resistance in *Pseudomonas aeruginosa*. *PLoS. Biol.* **2020**, *18(8)*, e3000805. DOI: 10.1371/journal.pbio.3000805.
39. Cystic Fibrosis Foundation Website, Drug Development Pipeline - Clinical Trials Tool. Available online: https://apps.cff.org/trials/pipeline (Accessed 19 February 2024).
40. Choi, V.; Rohn J.L.; Stoodley P.; Carugo D.; Stride E. Drug delivery strategies for antibiofilm therapy. *Nat. Rev. Microbiol.* **2023**, *21(9)*, 555-572. DOI: 10.1038/s41579-023-00905-2.
41. Mowat, E.; Paterson S.; Fothergill J.L.; Wright E.A.; Ledson M.J.; Walshaw M.J., et al. *Pseudomonas aeruginosa* population diversity and turnover in cystic fibrosis chronic infections. *Am. J. Respir. Crit Care Med.* **2011**, *183(12)*, 1674-1679. DOI: 10.1164/rccm.201009-1430OC.
42. Jiang, Y.; Geng M.; Bai L. Targeting biofilms therapy: current research strategies and development hurdles. *Microorganisms.* **2020**, *8(8)*. DOI: 10.3390/microorganisms8081222.
43. Chance, D.L.; Mawhinney T.P. Observations of, and insights into, cystic fibrosis mucus heterogeneity in the pre-modulator era: sputum characteristics, DNA and glycoprotein content, and solubilization time. *J. Respiration* **2021**, *1(1)*, 8-29. DOI: 10.3390/jor1010002.

44. Patil, P.D.; Zheng H.; Burns F.N.; Ibanez A.C.S.; Jin Y.; Luk Y.Y. Chimeric ligands of pili and lectin a inhibit tolerance, persistence, and virulence factors of *Pseudomonas aeruginosa* over a wide range of phenotypes. *ACS Infect. Dis.* **2022**. DOI: 10.1021/acsinfecdis.2c00201.
45. Rouillard, K.R.; Markovetz M.R.; Kissner W.J.; Boone W.L.; Plott L.M.; Hill D.B. Altering the viscoelastic properties of mucus-grown *Pseudomonas aeruginosa* biofilms affects antibiotic susceptibility. *Biofilm* **2023**, *5*, 100104. DOI: 10.1016/j.bioflm.2023.100104.
46. Bucior, I.; Abbott J.; Song Y.; Matthay M.A.; Engel J.N. Sugar administration is an effective adjunctive therapy in the treatment of *Pseudomonas aeruginosa* pneumonia. *Am. J. Physiol. Lung Cell Mol. Physiol.* **2013**, *305(5)*, L352-L363. DOI: 10.1152/ajplung.00387.2012.
47. Grishin, A.V.; Krivozubov M.S.; Karyagina A.S.; Gintsburg A.L. *Pseudomonas aeruginosa* lectins as targets for novel antibacterials. *Acta Naturae.* **2015**, *7(2)*, 29-41. DOI: 10.32607/20758251-2015-7-2-29-41.
48. Taccetti, G.; Francalanci M.; Pizzamiglio G.; Messore B.; Carnovale V.; Cimino G., et al. Cystic fibrosis: recent insights into inhaled antibiotic treatment and future perspectives. *Antibiotics (Basel)* **2021**, *10(3)*. DOI: 10.3390/antibiotics10030338.
49. Mawhinney T.P.; Chance D.L.; Waters J.K.; Mossine V.V.; He S.; Cassity N.A. Characterization of blood group antigen-containing oligosaccharides isolated from human respiratory mucous glycoproteins. In: Recent Developments in Carbohydrate Research, Volume 1; Pandalai SG, editor; Transworld Research Network. Kerala, India, **2003**; Volume 1, pp. p. 1-25.
50. Ehre, C.; Rushton Z.L.; Wang B.; Hothem L.N.; Morrison C.B.; Fontana N.C., et al. An improved inhaled mucolytic to treat airway muco-obstructive diseases. *Am. J. Respir. Crit Care Med.* **2019**, *199(2)*, 171-180. DOI: 10.1164/rccm.201802-0245OC.
51. Flume, P.A.; Amelina E.; Daines C.L.; Charlton B.; Leadbetter J.; Guasconi A., et al. Efficacy and safety of inhaled dry-powder mannitol in adults with cystic fibrosis: an international, randomized controlled study. *J. Cyst. Fibros.* **2021**, *20(6)*, 1003-1009. DOI: 10.1016/j.jcf.2021.02.011.
52. Michaud, G.; Visini R.; Bergmann M.; Salerno G.; Bosco R.; Gillon E., et al. Overcoming antibiotic resistance in *Pseudomonas aeruginosa* biofilms using glycopeptide dendrimers. *Chem. Sci.* **2016**, *7(1)*, 166-182. DOI: 10.1039/C5SC03635F.
53. Musken, M.; Pawar V.; Schwebs T.; Bahre H.; Felgner S.; Weiss S., et al. Breaking the vicious cycle of antibiotic killing and regrowth of biofilm-residing *Pseudomonas aeruginosa*. *Antimicrob. Agents Chemother.* **2018**, *62(12)*. DOI: 10.1128/AAC.01635-18.
54. Boffoli, D.; Bellato F.; Avancini G.; Gurnani P.; Yilmaz G.; Romero M., et al. Tobramycin-loaded complexes to prevent and disrupt *Pseudomonas aeruginosa* biofilms. *Drug Deliv. Transl. Res.* **2021**. DOI: 10.1007/s13346-021-01085-3.
55. Hauck, D.; Joachim I.; Frommeyer B.; Varrot A.; Philipp B.; Moller H.M., et al. Discovery of two classes of potent glycomimetic inhibitors of *Pseudomonas aeruginosa* LecB with distinct binding modes. *ACS Chem. Biol.* **2013**, *8(8)*, 1775-1784. DOI: 10.1021/cb400371r.
56. Maura, D.; Ballok A.E.; Rahme L.G. Considerations and caveats in anti-virulence drug development. *Curr. Opin. Microbiol.* **2016**, *33*, 41-46. DOI: 10.1016/j.mib.2016.06.001.
57. Zahorska, E.; Rosato F.; Stober K.; Kuhaudomlarp S.; Meiers J.; Hauck D., et al. Neutralizing the impact of the virulence factor LecA from *Pseudomonas aeruginosa* on human cells with new glycomimetic inhibitors. *Angew. Chem. Int. Ed Engl.* **2023**, *62(7)*, e202215535. DOI: 10.1002/anie.202215535.
58. Winzer, K.; Falconer C.; Garber N.C.; Diggle S.P.; Camara M.; Williams P. The *Pseudomonas aeruginosa* lectins PA-IL and PA-IIL are controlled by quorum sensing and by RpoS. *J. Bacteriol.* **2000**, *182(22)*, 6401-6411. DOI: 10.1128/jb.182.22.6401-6411.2000.
59. Singh, K.; Kulkarni S.S. Small carbohydrate derivatives as potent antibiofilm agents. *J. Med. Chem.* **2022**, *65(13)*, 8525-8549. DOI: 10.1021/acs.jmedchem.1c01039.
60. Imberty, A.; Wimmerova, M.; Mitchell E.P.; Gilboa-Garber N. Structures of the lectins from *Pseudomonas aeruginosa*: insights into the molecular basis for host glycan recognition. *Microbes & Infect.* **2004**, *6(2)*, 221-228. DOI: 10.1016/j.micinf.2003.10.016.
61. Sommer, R.; Rox K.; Wagner S.; Hauck D.; Henrikus S.S.; Newsad S., et al. Anti-biofilm agents against *Pseudomonas aeruginosa*: a structure-activity relationship study of C-glycosidic LecB inhibitors. *J. Med. Chem.* **2019**, *62(20)*, 9201-9216. DOI: 10.1021/acs.jmedchem.9b01120.
62. Meiers, J.; Zahorska E.; Rohrig T.; Hauck D.; Wagner S.; Titz A. Directing drugs to bugs: antibiotic-carbohydrate conjugates targeting biofilm-associated lectins of *Pseudomonas aeruginosa*. *J. Med. Chem.* **2020**, *63(20)*, 11707-11724. DOI: 10.1021/acs.jmedchem.0c00856.

63. Meiers, J.; Rox K.; Titz A. Lectin-targeted prodrugs activated by *Pseudomonas aeruginosa* for self-destructive antibiotic release. *J. Med. Chem.* **2022**, *65(20)*, 13988-14014. DOI: 10.1021/acs.jmedchem.2c01214.
64. Aguilar C.; Eichwald C.; Eberl L. Multicellularity in bacteria: from division of labor to biofilm formation. in: evolutionary transitions to multicellular life., Springer. Dordrecht, Netherlands, **2015**.
65. Scharfman, A.; Degroote S.; Beau J.; Lamblin G.; Roussel P.; Mazurier J. *Pseudomonas aeruginosa* binds to neoglycoconjugates bearing mucin carbohydrate determinants and predominantly to sialyl-Lewis x conjugates. *Glycobiol.* **1999**, *9*, 757-764. DOI: 10.1093/glycob/9.8.757.
66. Ramphal, R.; Arora S.K. Recognition of mucin components by *Pseudomonas aeruginosa*. *Glycoconj. J.* **2001**, *2002/10/19*, 709-713. DOI: 10.1023/a:1020823406840.
67. Jeffries, J.L.; Jia J.; Choi W.; Choe S.; Miao J.; Xu Y., et al. *Pseudomonas aeruginosa* pyocyanin modulates mucin glycosylation with sialyl-Lewis(x) to increase binding to airway epithelial cells. *Mucosal. Immunol.* **2016**, *9(4)*, 1039-1050. DOI: 10.1038/mi.2015.119.
68. Bucior, I.; Pielage J.F.; Engel J.N. *Pseudomonas aeruginosa* pili and flagella mediate distinct binding and signaling events at the apical and basolateral surface of airway epithelium. *PLoS. Pathog.* **2012**, *8(4)*, e1002616. DOI: 10.1371/journal.ppat.1002616.
69. Lillehoj, E.P.; Kim B.T.; Kim K.C. Identification of *Pseudomonas aeruginosa* flagellin as an adhesin for Muc1 mucin. *Am. J. Physiol. Lung Cell Mol. Physiol.* **2002**, *282(4)*, L751-L756. DOI: 10.1152/ajplung.00383.2001.
70. Chemani, C.; Imberty A.; de B.S.; Pierre M.; wimmerova M.; Guery B.P., et al. Role of LecA and LecB lectins in *Pseudomonas aeruginos*a-induced lung injury and effect of carbohydrate ligands. *Infect. Immun.* **2009**, *77(5)*, 2065-2075. DOI: 10.1128/IAI.01204-08.
71. Sheth, H.B.; Lee K.K.; Wong W.Y.; Srivastava G.; Hindsgaul O.; Hodges R.S., et al. The pili of *Pseudomonas aeruginosa* strains PAK and PAO bind specifically to the carbohydrate sequence βGalNAc(1-4)βGal found in glycosphingolipids asialo-GM1 and asialo-GM2. *Mol. Microbiol.* **1994**, *11(4)*, 715-723. DOI: 10.1111/j.1365-2958.1994.tb00349.x.
72. Ramphal, R.; Carnoy C.; Fievre S.; Michalski J.C.; Houdret N.; Lamblin G., et al. *Pseudomonas aeruginosa* recognizes carbohydrate chains containing type 1 (Gal β1-3GlcNAc) or type 2 (Gal β1-4GlcNAc) disaccharide units. *Infect. Immun.* **1991**, *59(2)*, 700-704. DOI: 10.1128/iai.59.2.700-704.1991.
73. Hahn, H.P. The type-4 pilus is the major virulence-associated adhesin of *Pseudomonas aeruginos*a--a review. *Gene* **1997**, *192(1)*, 99-108. DOI: 10.1016/s0378-1119(97)00116-9.
74. Lamblin, G.; Degroote S.; Perini J.M.; Delmotte P.; Scharfman A.; Davril M., et al. Human airway mucin glycosylation: a combinatory of carbohydrate determinants which vary in cystic fibrosis. *Glycoconj. J.* **2001**, *18(9)*, 661-684. DOI: 10.1023/a:1020867221861.
75. Scharfman, A.; Arora S.K.; Delmotte P.; Van B.E.; Mazurier J.; Ramphal R., et al. Recognition of Lewis x derivatives present on mucins by flagellar components of *Pseudomonas aeruginosa*. *Infect. Immun.* **2001**, *69(9)*, 5243-5248. DOI: 10.1128/IAI.69.9.5243-5248.2001.
76. Mewe, M.; Tielker D.; Schonberg R.; Schachner M.; Jaeger K.E.; Schumacher U. *Pseudomonas aeruginosa* lectins I and II and their interaction with human airway cilia. *J. Laryngol. Otol.* **2005**, *119(8)*, 595-599. DOI: 10.1258/0022215054516313.
77. Krivan, H.C.; Roberts D.D.; Ginsburg V. Many pulmonary pathogenic bacteria bind specifically to the carbohydrate sequence GalNAc *beta* 1-4Gal found in some glycolipids. *Proc. Natl. Acad. Sci. U. S. A* **1988**, *85(16)*, 6157-6161. DOI: 10.1073/pnas.85.16.6157.
78. Imundo, L.; Barasch J.; Prince A.; Al-Awqati Q. Cystic fibrosis epithelial cells have a receptor for pathogenic bacteria on their apical surface. *Proc. Natl. Acad. Sci. U. S. A* **1995**, *92(7)*, 3019-3023. DOI: 10.1073/pnas.92.7.3019.
79. Saiman, L.; Prince A. *Pseudomonas aeruginosa* pili bind to asialoGM1 which is increased on the surface of cystic fibrosis epithelial cells. *J. Clin. Invest.* **1993**, *92(4)*, 1875-1880. DOI: 10.1172/JCI116779.
80. Arora, S.K.; Ritchings B.W.; Almira E.C.; Lory S.; Ramphal R. The *Pseudomonas aeruginosa* flagellar cap protein, FliD, is responsible for mucin adhesion. *Infect. Immun.* **1998**, *66*, 1000-1007. DOI: 10.1128/iai.66.3.1000-1007.1998.
81. Scharfman, A.; Delmotte P.; Beau J.; Lamblin G.; Roussel P.; Mazurier J. Sialyl-Le(x) and sulfo-sialyl-Le(x) determinants are receptors for *P. aeruginosa*. *Glycoconj. J.* **2000**, *17(10)*, 735-740. DOI: 10.1023/a:1011091112884.
82. Davril, M.; Degroote S.; Humbert P.; Galabert C.; Dumur V.; Lafitte J.J., et al. The sialylation of bronchial mucins secreted by patients suffering from cystic fibrosis or from chronic bronchitis is related to the severity of airway infection. *Glycobiol.* **1999**, *9(3)*, 311-321. DOI: 10.1093/glycob/9.3.311.
83. Degroote, S.; Maes E.; Humbert P.; Delmotte P.; Lamblin G.; Roussel P. Sulfated oligosaccharides isolated from the respiratory mucins of a secretor patient suffering from chronic bronchitis. *Biochimie* **2003**, *85(3-4)*, 369-379. DOI: 10.1016/s0300-9084(03)00022-1.

84. Xia, B.; Royall J.A.; Damera G.; Sachdev G.P.; Cummings R.D. Altered *O*-glycosylation and sulfation of airway mucins associated with cystic fibrosis. *Glycobiol.* **2005**, *15(8)*, 747-775. DOI: 10.1093/glycob/cwi061.
85. Zanin, M.; Baviskar P.; Webster R.; Webby R. The interaction between respiratory pathogens and mucus. *Cell Host. Microbe* **2016**, *19(2)*, 159-168. DOI: 10.1016/j.chom.2016.01.001.
86. Adam, E.C.; Mitchell B.S.; Schumacher D.U.; Grant G.; Schumacher U. *Pseudomonas aeruginosa* II lectin stops human ciliary beating: therapeutic implications of fucose. *Am. J. Respir. Crit Care Med.* **1997**, *155(6)*, 2102-2104. DOI: 10.1164/ajrccm.155.6.9196121.
87. Adam, E.C.; Schumacher D.U.; Schumacher U. Cilia from a cystic fibrosis patient react to the ciliotoxic *Pseudomonas aeruginosa* II lectin in a similar manner to normal control cilia--a case report. *J. Laryngol. Otol.* **1997**, *111(8)*, 760-762. DOI: 10.1017/s0022215100138551.
88. Garber, N.C.; Zinger-Yosovich K.D.; Sudakevitz D.; Axelrad I.; Gilboa-Garber N. Regulation of lectin production by the human pathogens *Pseudomonas aeruginosa* and *Chromobacterium violaceum*: effects of choline, trehalose, and ethanol. *Adv. Exp. Med. Biol.* **2011**, *705*, 229-256. DOI: 10.1007/978-1-4419-7877-6_11.
89. Passos da Silva, D.; Matwichuk M.L.; Townsend D.O.; Reichhardt C.; Lamba D.; Wozniak D.J., et al. The *Pseudomonas aeruginosa* lectin LecB binds to the exopolysaccharide Psl and stabilizes the biofilm matrix. *Nature Commun.* **2019**, *10(1)*, 2183. DOI: 10.1038/s41467-019-10201-4.
90. Tielker, D.; Hacker S.; Loris R.; Strathmann M.; Wingender J.; Wilhelm S., et al. *Pseudomonas aeruginosa* lectin LecB is located in the outer membrane and is involved in biofilm formation. *Microbiol. (Reading)* **2005**, *151(Pt 5)*, 1313-1323. DOI: 10.1099/mic.0.27701-0.
91. Wu, A.M.; Wu J.H.; Singh T.; Liu J.H.; Tsai M.S.; Gilboa-Garber N. Interactions of the fucose-specific *Pseudomonas aeruginosa* lectin, PA-IIL, with mammalian glycoconjugates bearing polyvalent Lewis(a) and ABH blood group glycotopes. *Biochimie* **2006**, *88(10)*, 1479-1492. DOI: 10.1016/j.biochi.2006.05.004.
92. Funken, H.; Bartels K.M.; Wilhelm S.; Brocker M.; Bott M.; Bains M., et al. Specific association of lectin LecB with the surface of *Pseudomonas aeruginosa*: role of outer membrane protein OprF. *PLoS ONE* **2012**, *7*, e46857. DOI: 10.1371/journal.pone.0046857.
93. Gilboa-Garber, N. *Pseudomonas aeruginosa* lectins. *Methods Enzymol.* **1982**, *83*, 378-385.
94. Chen, C.P.; Song S.C.; Gilboa-Garber N.; Chang K.S.; Wu A.M. Studies on the binding site of the galactose-specific agglutinin PA-IL from *Pseudomonas aeruginosa*. *Glycobiol.* **1998**, *8(1)*, 7-16. DOI: 10.1093/glycob/8.1.7.
95. Gilboa-Garber, N.; Katcoff D.J.; Garber N.C. Identification and characterization of *Pseudomonas aeruginosa* PA-IIL lectin gene and protein compared to PA-IL. *FEMS Immunol. Med. Microbiol.* **2000**, *29(1)*, 53-57. DOI: 10.1111/j.1574-695X.2000.tb01505.x.
96. Cioci, G.; Mitchell E.P.; Gautier C.; Wimmerova M.; Sudakevitz D.; Perez S., et al. Structural basis of calcium and galactose recognition by the lectin PA-IL of *Pseudomonas aeruginosa*. *FEBS Lett.* **2003**, *555(2)*, 297-301. DOI: 10.1016/s0014-5793(03)01249-3.
97. Mitchell, E.; Houles C.; Sudakevitz D.; wimmerova M.; Gautier C.; Perez S., et al. Structural basis for oligosaccharide-mediated adhesion of *Pseudomonas aeruginosa* in the lungs of cystic fibrosis patients. *Nat. Struct. Biol.* **2002**, *9(12)*, 918-921. DOI: 10.1038/nsb865.
98. Boukerb, A.M.; Rousset A.; Galanos N.; Mear J.B.; Thepaut M.; Grandjean T., et al. Antiadhesive properties of glycoclusters against *Pseudomonas aeruginosa* lung infection. *J. Med. Chem.* **2014**, *57(24)*, 10275-10289. DOI: 10.1021/jm500038p.
99. Gustke, H.; Kleene R.; Loers G.; Nehmann N.; Jaehne M.; Bartels K.M., et al. Inhibition of the bacterial lectins of *Pseudomonas aeruginosa* with monosaccharides and peptides. *Eur. J. Clin. Microbiol. Infect. Dis.* **2012**, *31(2)*, 207-215. DOI: 10.1007/s10096-011-1295-x.
100. Von Bismarck, P.; Schneppenheim R.; Schumacher U. Successful treatment of *Pseudomonas aeruginosa* respiratory tract infection with a sugar solution-a case report on a lectin based therapeutic principle. *Klinische. Padiatrie.* **2001**, *213(05)*, 285-287. DOI: 10.1055/s-2001-17220.
101. Hauber, H.P.; Schulz M.; Pforte A.; Mack D.; Zabel P.; Schumacher U. Inhalation with fucose and galactose for treatment of *Pseudomonas aeruginosa* in cystic fibrosis patients. *Int. J. Med. Sci.* **2008**, *5*, 371-376. DOI: 10.7150/ijms.5.371.
102. Cassin, E.K.; Tseng B.S. Pushing beyond the envelope: the potential roles of OprF in *Pseudomonas aeruginosa* biofilm formation and pathogenicity. *J. Bacteriol.* **2019**, *201(18)*. DOI: 10.1128/JB.00050-19.
103. Kolomiets, E.; Swiderska M.A.; Kadam R.U.; Johansson E.M.; Jaeger K.E.; Darbre T., et al. Glycopeptide dendrimers with high affinity for the fucose-binding lectin LecB from *Pseudomonas aeruginosa*. *Chem. Med. Chem.* **2009**, *4(4)*, 562-569. DOI: 10.1002/cmdc.200800380.

104. Johansson, E.M.; Crusz S.A.; Kolomiets E.; Buts L.; Kadam R.U.; Cacciarini M., et al. Inhibition and dispersion of *Pseudomonas aeruginosa* biofilms by glycopeptide dendrimers targeting the fucose-specific lectin LecB. *Chem. Biol.* **2008**, *15(12)*, 1249-1257. DOI: 10.1016/j.chembiol.2008.10.009.
105. Bernardi, A.; Jimenez-Barbero J.; Casnati A.; De C.C.; Darbre T.; Fieschi F., et al. Multivalent glycoconjugates as anti-pathogenic agents. *Chem. Soc. Rev.* **2013**, *42(11)*, 4709-4727. DOI: 10.1039/c2cs35408j.
106. Reymond, J.L.; Bergmann M.; Darbre T. Glycopeptide dendrimers as *Pseudomonas aeruginosa* biofilm inhibitors. *Chem. Soc. Rev.* **2013**, *42(11)*, 4814-4822. DOI: 10.1039/c3cs35504g.
107. Kadam, R.U.; Bergmann M.; Hurley M.; Garg D.; Cacciarini M.; Swiderska M.A., et al. A glycopeptide dendrimer inhibitor of the galactose-specific lectin LecA and of *Pseudomonas aeruginosa* biofilms. *Angew. Chem. Int. Ed Engl.* **2011**, *50(45)*, 10631-10635. DOI: 10.1002/anie.201104342.
108. Cecioni, S.; Imberty A.; Vidal S. Glycomimetics versus multivalent glycoconjugates for the design of high affinity lectin ligands. *Chem. Rev.* **2015**, *115(1)*, 525-561. DOI: 10.1021/cr500303t.
109. Goyard, D.; Thomas B.; Gillon E.; Imberty A.; Renaudet O. Heteroglycoclusters with dual nanomolar affinities for the lectins LecA and LecB from *Pseudomonas aeruginosa*. *Front Chem.* **2019**, *7*, 666. DOI: 10.3389/fchem.2019.00666.
110. Malinovska, L.; Thai L.S.; Herczeg M.; Vaskova M.; Houser J.; Fujdiarova E., et al. Synthesis of β-D-galactopyranoside-presenting glycoclusters, investigation of their interactions with *Pseudomonas aeruginosa* Lectin A (PA-IL) and evaluation of their anti-adhesion potential. *Biomolecules* **2019**, *9(11)*. DOI: 10.3390/biom9110686.
111. Leusmann, S.; Menova P.; Shanin E.; Titz A.; Rademacher C. Glycomimetics for the inhibition and modulation of lectins. *Chem. Soc. Rev.* **2023**, *52(11)*, 3663-3740. DOI: 10.1039/d2cs00954d.
112. Rodrigue, J.; Ganne G.; Blanchard B.; Saucier C.; Giguere D.; Shiao T.C., et al. Aromatic thioglycoside inhibitors against the virulence factor LecA from *Pseudomonas aeruginosa*. *Org. & Biomolec. Chem.* **2013**, *11*, 6906-6918. DOI: 10.1039/c3ob41422a.
113. Zhao, Y.; Yu C.; Yu Y.; Wei X.; Duan X.; Dai X., et al. Bioinspired heteromultivalent ligand-decorated nanotherapeutic for enhanced photothermal and photodynamic therapy of antibiotic-resistant bacterial pneumonia. *ACS Appl. Mater. Interfaces.* **2019**, *11(43)*, 39648-39661. DOI: 10.1021/acsami.9b15118.
114. Sommer, R.; Wagner S.; Rox K.; Varrot A.; Hauck D.; Wamhoff E.C., et al. Glycomimetic, Orally bioavailable LecB inhibitors block biofilm formation of *Pseudomonas aeruginosa*. *J. Am. Chem. Soc.* **2018**, *140(7)*, 2537-2545. DOI: 10.1021/jacs.7b11133.
115. Bartels, K.M.; Funken H.; Knapp A.; Brocker M.; Bott M.; Wilhelm S., et al. Glycosylation is required for outer membrane localization of the lectin LecB in *Pseudomonas aeruginosa*. *J. Bacteriol.* **2011**, *193(5)*, 1107-1113. DOI: 10.1128/JB.01507-10.
116. Kautto, L.; Nguyen-Khuong T.; Everest-Dass A.; Leong A.; Zhao Z.; Willcox M.D.P., et al. Glycan involvement in the adhesion of *Pseudomonas aeruginosa* to tears. *Exp. Eye Res.* **2016**, *145* , 278-288. DOI: 10.1016/j.exer.2016.01.013.
117. Boukerb, A.M.; Decor A.; Ribun S.; Tabaroni R.; Rousset A.; Commin L., et al. Genomic rearrangements and functional diversification of *lec*a and *lec*b lectin-coding regions impacting the efficacy of glycomimetics directed against *Pseudomonas aeruginosa*. *Front Microbiol.* **2016**, *7*, 811. DOI: 10.3389/fmicb.2016.00811.
118. Sommer, R.; Wagner S.; Varrot A.; Nycholat C.M.; Khaledi A.; Haussler S., et al. The virulence factor LecB varies in clinical isolates: consequences for ligand binding and drug discovery. *Chem. Sci.* **2016**, *7(8)*, 4990-5001. DOI: 10.1039/c6sc00696e.
119. Wang, W.; Chance D.L.; Mossine V.V.; Mawhinney T.P. RAFT-based tri-component fluorescent glycopolymers: synthesis, characterization and application in lectin-mediated bacterial binding study. *Glycoconj. J.* **2014**, *31(2)*, 133-143. DOI: 10.1007/s10719-013-9508-4.
120. Reynolds, D.D.; Evans W.L. β- D-glucose-1,2,3,4-tetraacetate. *Org. synth.* **1942**, *22*, 56-58. DOI: 10.15227/orgsyn.022.0056.
121. Uchiyama, T.; Hindsgaul O. Per-O-trimethylsilyl-a-l-fucopyranosyl iodide: a novel glycosylating agent for terminal α-L-fucosylation. *Synlett.* **1996**, *1996*, 499-501. DOI: 10.1055/s-1996-5491.
122. Holloway, B.W. Genetic recombination in *Pseudomonas aeruginosa*. *J. Gen. Microbiol.* **1955**, *13(3)*, 572-581. DOI: 10.1099/00221287-13-3-572.
123. Hampton, K.D.; Wasilauskas B.L. Isolation of oxidase-negative *Pseudomonas aeruginosa* from sputum culture. *J. Clin. Microbiol.* **1979**, *9(5)*, 632-634. DOI: 10.1128/jcm.9.5.632-634.1979.
124. Chance, D.L.; Mawhinney T.P. Carbohydrate sulfation effects on growth of *Pseudomonas aeruginosa*. *Microbiol.* **2000**, *146 (Pt 7)*, 1717-1725. DOI: 10.1099/00221287-146-7-1717.
125. Klockgether, J.; Munder A.; Neugebauer J.; Davenport C.F.; Stanke F.; Larbig K.D., et al. Genome diversity of *Pseudomonas aeruginosa* PAO1 laboratory strains. *J. Bacteriol.* **2010**, *192(4)*, 1113-1121. DOI: 10.1128/JB.01515-09.
126. Sambrook J, Fritsch EF, Maniatis T. Molecular cloning: a laboratory manual. 2nd ed. Cold Spring Harbor, N.Y.: Cold Spring Harbor Laboratory; **1989**.

127. O'Toole, G.A.; Kolter R. Flagellar and twitching motility are necessary for *Pseudomonas aeruginosa* biofilm development. *Mol. Microbiol.* **1998**, *30(2)*, 295-304. DOI: 10.1046/j.1365-2958.1998.01062.x.
128. Chance, D.L.; Mawhinney T.P. Using negative staining TEM to study structure/function relationships of cystic fibrosis host-adapted opportunistic pathogen *Pseudomonas aeruginosa*. *Microscopy & Microanal.* **2017**, *2017/08/04(S1)*, 1354-1355. DOI: 10.1017/S1431927617007437.
129. Chance, D.; Wang W.; Mawhinney T. Probing cystic fibrosis *Pseudomonas aeruginosa* binding data for features that contribute to enhanced associations with α-Gal and β-GalNAc pendant monosaccharides of fluorescent glycopolymers. *J. Cyst. Fibros.* **2021**, *20*, S244. DOI: 10.1016/S1569-1993(21)01940-8.
130. Morris, A.J.; Jackson L.; Cw Y.Y.; Reichhardt C.; Beaudoin T.; Uwumarenogie S., et al. The role of Psl in the failure to eradicate *Pseudomonas aeruginosa* biofilms in children with cystic fibrosis. *NPJ. Biofilms. Microbiomes.* **2021**, *7(1)*, 63. DOI: 10.1038/s41522-021-00234-3.
131. Deligianni, E.; Pattison S.; Berrar D.; Ternan N.G.; Haylock R.W.; Moore J.E., et al. *Pseudomonas aeruginosa* cystic fibrosis isolates of similar RAPD genotype exhibit diversity in biofilm forming ability *in vitro*. *BMC. Microbiol.* **2010**, *10*, 38. DOI: 10.1186/1471-2180-10-38.
132. Nolan, C.; Behrends V. Sub-inhibitory antibiotic exposure and virulence in *Pseudomonas aeruginosa*. *Antibiotics* **2021**, *10(11)*, 1393. DOI: 10.3390/antibiotics10111393.
133. Bjarnsholt, T.; Whiteley M.; Rumbaugh K.P.; Stewart P.S.; Jensen P.O.; Frimodt-Moller N. The importance of understanding the infectious microenvironment. *Lancet Infect. Dis.* **2022**, *22(3)*, e88-e92. DOI: 10.1016/S1473-3099(21)00122-5.
134. Cullen, L.; Weiser R.; Olszak T.; Maldonado R.F.; Moreira A.S.; Slachmuylders L., et al. Phenotypic characterization of an international *Pseudomonas aeruginosa* reference panel: strains of cystic fibrosis (CF) origin show less *in vivo* virulence than non-CF strains. *Microbiol.* **2015**, *161(10)*, 1961-1977. DOI: 10.1099/mic.0.000155.
135. Smith, E.E.; Buckley D.G.; Wu Z.; Saenphimmachak C.; Hoffman L.R.; D'Argenio D.A., et al. Genetic adaptation by *Pseudomonas aeruginosa* to the airways of cystic fibrosis patients. *Proc. Natl. Acad. Sci. U. S. A* **2006**, *103(22)*, 8487-8492. DOI: 10.1073/pnas.0602138103.
136. Reichhardt, C.; Jacobs H.M.; Matwichuk M.; Wong C.; Wozniak D.J.; Parsek M.R. The versatile *Pseudomonas aeruginosa* biofilm matrix protein CdrA promotes aggregation through different extracellular exopolysaccharide interactions. *J. Bacteriol.* **2020**, *202(19)*. DOI: 10.1128/JB.00216-20.
137. Vishwanath, S.; Guay C.M.; Ramphal R. Effects of subminimal inhibitory concentrations of antibiotics on the adherence of *Pseudomonas aeruginosa* to tracheobronchial mucin. *J. Antimicrob. Chemother.* **1987**, *19(5)*, 579-583. DOI: 10.1093/jac/19.5.579.
138. Carfartan, G.; Gerardin P.; Turck D.; Husson M.O. Effect of subinhibitory concentrations of azithromycin on adherence of *Pseudomonas aeruginosa* to bronchial mucins collected from cystic fibrosis patients. *J. Antimicrob. Chemother.* **2004**, *53(4)*, 686-688. DOI: 10.1093/jac/dkh133.
139. Mojsoska, B.; Ghoul M.; Perron G.G.; Jenssen H.; Alatraktchi F.A. Changes in toxin production of environmental *Pseudomonas aeruginosa* isolates exposed to sub-inhibitory concentrations of three common antibiotics. *PLoS. One.* **2021**, *16(3)*, e0248014. DOI: 10.1371/journal.pone.0248014.
140. Mossine, V.V.; Chance D.L.; Waters J.K.; Mawhinney T.P. Interaction of bacterial phenazines with colistimethate in bronchial epithelial cells. *Antimicrob. Agents Chemother.* **2018**, *62(8)*, e02349-17. DOI: 10.1128/AAC.02349-17.
141. Chance, D.; Wang W.; Mawhinney T. Targeting cystic fibrosis *Pseudomonas aeruginosa* with galactose related glycomimics: α- and ß-anomeric configurations, *N*-acetylation, and sulfation in whole-cell binding. *J. Cyst. Fibros.* **2023**, *22(S3)*, S2. DOI: 10.1016/S1569-1993(23)00937-2.
142. Lowery, N.V.; McNally L.; Ratcliff W.C.; Brown S.P. Division of labor, bet hedging, and the evolution of mixed biofilm investment strategies. *mBio.* **2017**, *8(4)*. DOI: 10.1128/mBio.00672-17.
143. El Meouche, I.; Siu Y.; Dunlop M.J. Stochastic expression of a multiple antibiotic resistance activator confers transient resistance in single cells. *Sci. Rep.* **2016**, *6*, 19538. DOI: 10.1038/srep19538.
144. Papa, R.; Imperlini E.; Trecca M.; Paris I.; Vrenna G.; Artini M., et al. Virulence of *Pseudomonas aeruginosa* in cystic fibrosis: relationships between normoxia and anoxia lifestyle. *Antibiotics (Basel)* **2023**, *13(1)*. DOI: 10.3390/antibiotics13010001.
145. Zopf, D.; Roth S. Oligosaccharide anti-infective agents. *Lancet* **1996**, *347(9007)*, 1017-1021. DOI: 10.1016/s0140-6736(96)90150-6.
146. Loris, R.; Tielker D.; Jaeger K.E.; Wyns L. Structural basis of carbohydrate recognition by the lectin LecB from *Pseudomonas aeruginosa*. *J. Mol. Biol.* **2003**, *331(4)*, 861-870. DOI: 10.1016/s0022-2836(03)00754-x.
147. Heggelund, J.E.; Varrot A.; Imberty A.; Krengel U. Histo-blood group antigens as mediators of infections. *Curr. Opin. Struct. Biol.* **2017**, *44*, 190-200. DOI: 10.1016/j.sbi.2017.04.001.

148. de Bentzmann, S.; Roger P.; Dupuit F.; Bajolet-Laudinat O.; Fuchey C.; Plotkowski M.C., et al. Asialo GM1 is a receptor for *Pseudomonas aeruginosa* adherence to regenerating respiratory epithelial cells. *Infect. Immun.* **1996**, *64(5)*, 1582-1588. DOI: 10.1128/iai.64.5.1582-1588.1996.
149. Chance, D.L.; Mawhinney T.P. Disulfated oligosaccharides derived from tracheobronchial mucous glycoproteins of a patient suffering from cystic fibrosis. *Carbohydr. Res.* **1996**, *295*, 157-177. DOI: 10.1016/S0008-6215(96)90136-6.

References - #s in Supplemental File S6 only

S1. De Boeck K. Cystic fibrosis in the year 2020: a disease with a new face. *Acta Paediatr*. **2020**, 00, 1-7. DOI: 10.1111/apa.15155.
S2. Khan, M.A.; Ali Z.S.; Sweezey N.; Grasemann H.; Palaniyar N. Progression of cystic fibrosis lung disease from childhood to adulthood: neutrophils, neutrophil extracellular trap (NET) formation, and NET degradation. *Genes (Basel)* **2019**, *10(3)*, 183. DOI: 10.3390/genes10030183.
S.3. Filkins, L.M.; O'Toole G.A. Cystic fibrosis lung infections: polymicrobial, complex, and hard to treat. *PLoS. Pathog.* **2015**, *11(12)*, e1005258. DOI: 10.1371/journal.ppat.1005258.
S4. Bacci, G.; Taccetti G.; Dolce D.; Armanini F.; Segata N.; Di C.F., et al. Untargeted metagenomic investigation of the airway microbiome of cystic fibrosis patients with moderate-severe lung disease. *Microorganisms* **2020**, *8(7)*. DOI: 10.3390/microorganisms8071003.
S5. Linnane, B.; Walsh A.M.; Walsh C.J.; Crispie F.; O'Sullivan O.; Cotter P.D., et al. The lung microbiome in young children with cystic fibrosis: a prospective cohort study. *Microorganisms* **2021**, *9(3)*. DOI: 10.3390/microorganisms9030492.
S6. Moradali, M.F.; Ghods S.; Rehm B.H. *Pseudomonas aeruginosa* Lifestyle: a paradigm for adaptation, survival, and persistence. *Front Cell Infect. Microbiol.* **2017**, *7*, 39. DOI: 10.3389/fcimb.2017.00039.
S7. Maurice, N.M.; Bedi B.; Sadikot R.T. *Pseudomonas aeruginosa* biofilms: host response and clinical implications in lung infections. *Am. J. Respir. Cell Mol. Biol.* **2018**, *58(4)*, 428-439. DOI: 10.1165/rcmb.2017-0321TR.
S8. Landry, R.M.; An D.; Hupp J.T.; Singh P.K.; Parsek M.R. Mucin-*Pseudomonas aeruginosa* interactions promote biofilm formation and antibiotic resistance. *Mol. Microbiol.* **2006**, *59(1)*, 142-151. DOI: 10.1111/j.1365-2958.2005.04941.x.
S9. Xia, B.; Sachdev G.P.; Cummings R.D. *Pseudomonas aeruginosa* mucoid strain 8830 binds glycans containing the sialyl-Lewis x epitope. *Glycoconj. J.* **2007**, *24(1)*, 87-95. DOI: 10.1007/s10719-006-9015-y.
S10. Ciofu, O.; Moser C.; Jensen P.O.; Hoiby N. Tolerance and resistance of microbial biofilms. *Nat. Rev. Microbiol.* **2022**, *20(10)*, 621-635. DOI: 10.1038/s41579-022-00682-4.
S11. Franklin, M.J.; Nivens D.E.; Weadge J.T.; Howell P.L. Biosynthesis of the *Pseudomonas aeruginosa* extracellular polysaccharides, alginate, Pel, and Psl. *Front Microbiol.* **2011**, *2*, 167. DOI: 10.3389/fmicb.2011.00167.
S12. Daboor, S.M.; Rohde J.R.; Cheng Z. Disruption of the extracellular polymeric network of *Pseudomonas aeruginosa* biofilms by alginate lyase enhances pathogen eradication by antibiotics. *J. Cyst. Fibros.* **2020**. DOI: 10.1016/j.jcf.2020.04.006.
S13. Malhotra, S.; Limoli D.H.; English A.E.; Parsek M.R.; Wozniak D.J. Mixed communities of mucoid and nonmucoid *Pseudomonas aeruginosa* exhibit enhanced resistance to host antimicrobials. *mBio* **2018**, *9(2)*. DOI: 10.1128/mBio.00275-18.
S14. Gunn, J.S.; Bakaletz L.O.; Wozniak D.J. What's on the outside matters: the role of the extracellular polymeric substance of gram-negative biofilms in evading host immunity and as a target for therapeutic intervention. *J. Biol. Chem.* **2016**, *291(24)*, 12538-12546. DOI: 10.1074/jbc.R115.707547.
S15. Wang, S.; Liu X.; Liu H.; Zhang L.; Guo Y.; Yu S., et al. The exopolysaccharide Psl-eDNA interaction enables the formation of a biofilm skeleton in *Pseudomonas aeruginosa*. *Environ. Microbiol. Rep* **2015**, *7(2)*, 330-340. DOI: 10.1111/1758-2229.12252.
S16. Dickey, S.W.; Cheung G.Y.C.; Otto M. Different drugs for bad bugs: antivirulence strategies in the age of antibiotic resistance. *Nat. Rev. Drug Discov.* **2017** , *16(7)*, 457-471. DOI: 10.1038/nrd.2017.23.
S17. Drenkard, E.; Ausubel F.M. *Pseudomonas* biofilm formation and antibiotic resistance are linked to phenotypic variation. *Nature* **2002**, *2002/04/19*, 740-743. DOI: 10.1038/416740a.
S18. Mould, D.L.; Hogan D.A. Intraspecies heterogeneity in microbial interactions. *Curr. Opin. Microbiol.* **2021**, *62*, 14-20. DOI: 10.1016/j.mib.2021.04.003.
S19. Boles, B.R.; Thoendel M.; Singh P.K. Self-generated diversity produces "insurance effects" in biofilm communities. *Proc. Natl. Acad. Sci. U. S. A* **2004**, *101(47)*, 16630-16635. DOI: 10.1073/pnas.0407460101.

S20. Smith, W.D.; Bardin E.; Cameron L.; Edmondson C.L.; Farrant K.V.; Martin I., et al. Current and future therapies for *Pseudomonas aeruginosa* infection in patients with cystic fibrosis. *FEMS Microbiol. Lett.* **2017**, *364(14)*. DOI: 10.1093/femsle/fnx121.
S21. Welp, A.L.; Bomberger J.M. Bacterial community interactions during chronic respiratory disease. *Front Cell Infect. Microbiol.* **2020**, *10*, 213. DOI: 10.3389/fcimb.2020.00213.
S22. Camus, L.; Briaud P.; Vandenesch F.; Moreau K. How bacterial adaptation to cystic fibrosis environment shapes interactions between *Pseudomonas aeruginosa* and *Staphylococcus aureus*. *Front Microbiol.* **2021**, *12*, 617784. DOI: 10.3389/fmicb.2021.617784.
S23. Venkatakrishnan, V.; Thaysen-Andersen M.; Chen S.C.A.; Nevalainen H.; Packer N.H. Cystic fibrosis and bacterial colonization define the sputum N-glycosylation phenotype. *Glycobiol.* **2015**, *25(1)*, 88-100. DOI: 10.1093/glycob/cwu092.
S24. Schulz, B.L.; Sloane A.J.; Robinson L.J.; Prasad S.S.; Lindner R.A.; Robinson M., et al. Glycosylation of sputum mucins is altered in cystic fibrosis patients. *Glycobiol.* **2007**, *17*, 698-712. DOI: 10.1093/glycob/cwm036.
S25. Soong, G.; Muir A.; Gomez M.I.; Waks J.; Reddy B.; Planet P., et al. Bacterial neuraminidase facilitates mucosal infection by participating in biofilm production. *J. Clin. Invest* **2006**, *116(8)*, 2297-2305. DOI: 10.1172/JCI27920.
S26. Stewart, P.S.; Franklin M.J. Physiological heterogeneity in biofilms. *Nature Rev. Microbiol.* **2008**, *2008/02/12*, 199-210. DOI: 10.1038/nrmicro1838.
S27. Behzadi, P.; Barath Z.; Gajdacs M. It's not easy being green: a narrative review on the microbiology, virulence and therapeutic prospects of multidrug-resistant *Pseudomonas aeruginosa*. *Antibiotics (Basel)* **2021**, *10(1)*. DOI: 10.3390/antibiotics10010042.
S28. Bovin, N.V. Polyacrylamide-based glycoconjugates as tools in glycobiology. *Glycoconj. J.* **1998**, *15(5)*, 431-446. DOI: 10.1023/a:1006963717646.
S29. Lipidmaps.org. Available online: https://www.lipidmaps.org/ (Accessed 19 February 2024).
S30. Worstell, N.C.; Singla A.; Saenkham P.; Galbadage T.; Sule P.; Lee D., et al. Hetero-Multivalency of *Pseudomonas aeruginosa* lectin LecA binding to model membranes. *Sci. Rep.* **2018**, *8(1)* , 8419. DOI: 10.1038/s41598-018-26643-7.
S31. Harrison, F.; McNally A.; da Silva A.C.; Heeb S.; Diggle S.P. Optimised chronic infection models demonstrate that siderophore 'cheating' in *Pseudomonas aeruginosa* is context specific. *ISME J.* **2017**, *11(11)*, 2492-2509. DOI: 10.1038/ismej.2017.103.
S32. Wagner, S.; Hauck D.; Hoffmann M.; Sommer R.; Joachim I.; Muller R., et al. Covalent lectin inhibition and application in bacterial biofilm imaging. *Angew. Chem. Int. Ed Engl.* **2017**, *56(52)*, 16559-16564. DOI: 10.1002/anie.201709368.
S33. Thomas, B.; Yan K.C.; Hu X.L.; Donnier-Marechal M.; Chen G.R.; He X.P., et al. Fluorescent glycoconjugates and their applications. *Chem. Soc. Rev.* **2020**, *49(2)*, 593-641. DOI: 10.1039/c8cs00118a.

References - Additional Relevant Resources

Chatterjee, M.; Anju C.P.; Biswas L.; Anil K., V; Gopi M.C.; Biswas R. Antibiotic resistance in *Pseudomonas aeruginosa* and alternative therapeutic options. *Int. J. Med. Microbiol.* **2016**, *306(1)*, 48-58. DOI: 10.1016/j.ijmm.2015.11.004.

Carnoy, C.; Ramphal R.; Scharfman A.; Lo-Guidice J.M.; Houdret N.; Klein A., et al. Altered carbohydrate composition of salivary mucins from patients with cystic fibrosis and the adhesion of *Pseudomonas aeruginosa*. *Am. J. Respir. Cell Mol. Biol.* **1993**, *9(3)*, 323-334. DOI: 10.1165/ajrcmb/9.3.323.

Thomas, R.; Brooks T. Common oligosaccharide moieties inhibit the adherence of typical and atypical respiratory pathogens. *J. Med. Microbiol.* **2004**, *53(Pt 9)*, 833-840. DOI: 10.1099/jmm.0.45643-0.

Comolli, J.C.; Waite L.L.; Mostov K.E.; Engel J.N. Pili binding to asialo-GM1 on epithelial cells can mediate cytotoxicity or bacterial internalization by *Pseudomonas aeruginosa*. *Infect. Immun.* **1999**, *67(7)*, 3207-3214. DOI: 10.1128/IAI.67.7.3207-3214.1999.
